# Supplementary material for: Global extent and drivers of mammal population declines in protected areas under illegal hunting pressure
Source: PLoS One. 2020 Aug 21;15(8):e0227163. doi: 10.1371/journal.pone.0227163 (PMC7446782; doi:10.1371/journal.pone.0227163)
Supplement: S1 File — (DOCX) [file pone.0227163.s001.docx]

## **SUPPLEMENTARY MATERIAL**

## **PART I: LIST OF ADDITIONAL SOURCES OF DATA FOR SPECIES BODY MASS OF VARIOUS MAMMAL SPECIES**

1. Cardini, A. & S. Elton. 2008. Variation in guenon skulls (I): species divergence, ecological and genetic differences. *Journal of Human Evolution*, 54: 615-637.
2. Grubb, P., T. Butynski, J. Oates, S. Bearder, T. Disotell, C. Groves, T. Struhsaker. 2003. Assessment of the diversity of African primates. *International Journal of Primatology*, 24: 1301-1357.
3. Kingdon, J. 1997. The Kingdon Field Guide to African Mammals. London: Academic Press.
4. O'Mara, M.T.; Gordon A.D.; Catlett K.K.; Terranova CJ; Schwartz G.T. 2012. Growth and the Development of Sexual Size Dimorphism in Lorises and Galagos. *American Journal of Physical Anthropology* **147**: 11–20
5. Nowak, R. M., editor 1999. *Walker's Mammals of the World.* Vol. 1. 6th edition. Pp. 264–271
6. Smith, A. T., Xie, Y. (eds.) 2008. [*A Guide to the Mammals of China*](http://books.google.com/books?id=ka-9f68nPT4C&pg=PA472&lpg=PA472&source=bl&ots=H7HGZRqSmj&sig=D_H9iLWAUXBKKBf7zolE3kY5INQ&hl=en#v=onepage&q&f=false). Princeton University Press, Princeton Oxforshire. Page 472.
7. Razafindratsima O.H., Jones, T.A. & Dunham, A.E. 2013. Patterns of Movement and Seed Dispersal by Three Lemur Species. *American Journal of Primatology*, 1-13
8. Pollock C. M. & R. E. Shadwick 1994. Relationship between body mass and biomechanical properties of limb tendons in adult mammals . *Am J Physiol Regulatory Integrative Comp Physiol* 266:1016-1021
9. Wilson, D. & D. Reeder. 2005. Mammal Species of the World. A Taxonomic and Geographic Reference (3rd ed). Johns Hopkins University: Johns Hopkins University Press <http://www.bucknell.edu/msw3/browse.asp?id=14200198> [accessed on 16, April, 2015]
10. Ferraz, K. M. P. M. D., Bonach, K., & Verdade, L. M. (2005). Relationship between body mass and body length in capybaras (Hydrochoerus hydrochaeris). *Biota Neotropica*, *5*(1), 197-200.
11. Junge, R. E., Dutton, C. J., Knightly, F., Williams, C. V., Rasambainarivo, F. T., & Louis, E. E. (2008). Comparison of biomedical evaluation for white-fronted brown lemurs (Eulemur fulvus albifrons) from four sites in Madagascar. *Journal of Zoo and Wildlife Medicine*, *39*(4), 567-575.
12. Freudenthal, M., & Martín-Suárez, E. (2013). Estimating body mass of fossil rodents. *Scripta Geologica*, (145).
13. Torregrosa, V., Petrucci, M., Pérez-Claros, J. A., & Palmqvist, P. (2010). Nasal aperture area and body mass in felids: Ecophysiological implications and paleobiological inferences. *Geobios*, *43*(6), 653-661.

1. Gordon, A. D., Johnson, S. E., & Louis, E. E. (2016). Environmental correlates of body mass in true lemurs (Eulemur spp.). *International Journal of Primatology*, *37*(1), 89-108.
2. Mohamed, B. A. A. (2018). *A Study on Some Biological Aspects of Dorcas gazelle (Gazella dorcas) under captive conditions* (Doctoral dissertation, Sudan University of Science and Technology).
3. Algadafi, W. (2019). The conservation ecology of the Dorcas gazelle (Gazella dorcas) in North East Libya.

**PART II: SEARCH TERMS USED IN DIFFERENT LITERATURE DATABASES**

## **Google Scholar**

poaching "protected area" reserve "illegal activities|hunting|activity" -marine mammal national park

## **Web of Science (SCI-EXPANDED indices)**

TS=("illegal activity" OR "illegal activities" OR "illegal hunting" OR poach*) AND TS=("protected area" OR reserve OR “national park OR "biodiversity outcome") AND DOCUMENT TYPES: (Article OR Letter)

## **Scopus**

TITLE-ABS-KEY ( poaching OR "illegal activity" OR "illegal activities" OR "illegal hunting" ) AND TITLE-ABS-KEY ( "protected area" OR reserve OR “National park” OR "biodiversity outcome" ) AND ( LIMIT-TO ( SRCTYPE , "j" ) ) AND ( LIMIT-TO ( SUBJAREA , "AGRI" ) OR LIMIT-TO ( SUBJAREA , "ENVI" ) OR LIMIT-TO ( SUBJAREA , "MULT" ) OR LIMIT-TO ( SUBJAREA , "EART" ) OR EXCLUDE ( SUBJAREA , "BIOC" ) OR EXCLUDE ( SUBJAREA , "ECON" ) OR EXCLUDE ( SUBJAREA , "ENER" ) OR EXCLUDE ( SUBJAREA , "ARTS" ) OR EXCLUDE ( SUBJAREA , "NEUR" ) OR EXCLUDE ( SUBJAREA , "MEDI" ) OR EXCLUDE ( SUBJAREA , "ENGI" ) OR EXCLUDE ( SUBJAREA , "VETE" ) OR EXCLUDE ( SUBJAREA , "DECI" ) OR EXCLUDE (SUBJAREA , "MATH" ) OR EXCLUDE ( SUBJAREA , "PHAR" ) OR EXCLUDE ( SUBJAREA , "PHYS" ) OR EXCLUDE ( SUBJAREA , "SOCI" ) ) AND ( LIMIT-TO ( DOCTYPE , "ar" ) OR LIMIT-TO ( DOCTYPE , "no" ) OR LIMIT-TO ( DOCTYPE , "le" ) )

**PART III: LIST OF 81 PAPERS REVIEWED**

1. Mitchell, D., Uganda Elephants near Extinction. Environmental Conservation, 1980. **7**(3): p. 212-212.

2. Borner, M., Black Rhino Disaster in Tanzania. Oryx, 1981. **16**(01): p. 59-66.

3. Western, D. and L. Vigne, The deteriorating status of African rhinos. Oryx, 1985. **19**(04): p. 215-220.

4. Douglas-Hamilton, I., African elephants: population trends and their causes. Oryx, 1987. **21**(01): p. 11-24.

5. Leader-Williams, N., S.D. Albon, and P.S.M. Berry, Illegal exploitation of black rhinoceros and elephant populations - patterns of decline, law-enforcement and patrol effort in Luangwa valley, Zambia. Journal of Applied Ecology, 1990. **27**(3): p. 1055-1087.

6. Alers, M.P.T., et al., Preliminary assessment of the status of the forest elephant in Zaire. African Journal of Ecology, 1992. **30**(4): p. 279-291.

7. Barnes, R.F.W., et al., Elephants and ivory poaching in the forests of equatorial Africa. Oryx, 1993. **27**(1): p. 27-34.

8. Michelmore, F., et al., A MODEL ILLUSTRATING THE CHANGES IN FOREST ELEPHANT NUMBERS CAUSED BY POACHING. African Journal of Ecology, 1994. **32**(2): p. 89-99.

9. Arcese, P., J. Hando, and K. Campbell, Historical and present-day anti-poaching efforts in Serengeti. In Serengeti II: dynamics, management, and conservation of an ecosystem. Chicago and London, University of Chicago Press. pp. 506-533. 1995.

10. Njiforti, H.L., Preferences and present demand for bushmeat in north Cameroon: Some implications for wildlife conservation. Environmental Conservation, 1996. **23**(2): p. 149-155.

11. Jachmann, H. and M. Billiouw, Elephant Poaching and Law Enforcement in the Central Luangwa Valley, Zambia. Journal of Applied Ecology, 1997. **34**(1): p. 233-244.

12. Plumptre, A.J., et al., The effects of the Rwandan civil war on poaching of ungulates in the Parc National des Volcans. Oryx, 1997. **31**(4): p. 265-273.

13. Wright, S.J., et al., Poachers Alter Mammal Abundance, Seed Dispersal, and Seed Predation in a Neotropical Forest. Conservation Biology, 2000. **14**(1): p. 227-239.

14. Ngandjui, G. and C.P. Blanc, Effects of hunting on mammalian (Mammalia) populations in the western sector of the Dja reserve (southern Cameroon). Game and Wildlife Science, 2000. **17**(2): p. 93-113.

15. Carpaneto, G.M. and A. Fusari, Subsistence hunting and bushmeat exploitation in central-western Tanzania. Biodiversity and Conservation, 2000. **9**(11): p. 1571-1585.

16. Badhwa, A., Tiger at the crossroads. Indian Forester, 2002. **128**(10): p. 1106-1112.

17. Loibooki, M., et al., Bushmeat hunting by communities adjacent to the Serengeti National Park, Tanzania: the importance of livestock ownership and alternative sources of protein and income. Environ. Conserv., 2002. **29**: p. 391.

18. Yamagiwa, J., Bushmeat Poaching and the Conservation Crisis in Kahuzi-Biega National Park, Democratic Republic of the Congo. Journal of Sustainable Forestry, 2003. **16**(3-4): p. 111-130.

19. García, G. and S.M. Goodman, Hunting of protected animals in the Parc National d'Ankarafantsika, north-western Madagascar. Oryx, 2003. **37**(01): p. 115-118.

20. Karamanlidis, A.A., et al., Monitoring human activity in an area dedicated to the protection of the endangered Mediterranean monk seal. Coastal Management, 2004. **32**(3): p. 293-306.

21. Rao, M., et al., Hunting patterns in tropical forests adjoining the Hkakaborazi National Park, north Myanmar. Oryx, 2005. **39**(03): p. 292-300.

22. Nielsen, M.R., Importance, cause and effect of bushmeat hunting in the Udzungwa Mountains, Tanzania: Implications for community based wildlife management. Biological Conservation, 2006. **128**(4): p. 509-516.

23. De Merode, E. and G. Cowlishaw, Species protection, the changing informal economy, and the politics of access to the bushmeat trade in the Democratic Republic of Congo. Conservation Biology, 2006. **20**(4): p. 1262-1271.

24. Hilborn, R., et al., Effective enforcement in a conservation area. Science, 2006. **314**(5803): p. 1266-1266.

25. Wato, Y.A., G.M. Wahungu, and M.M. Okello, Correlates of wildlife snaring patterns in Tsavo West National Park, Kenya. Biological Conservation, 2006. **132**(4): p. 500-509.

26. Blake, S., et al., Forest elephant crisis in the Congo Basin. Plos Biology, 2007. **5**(4): p. 945-953.

27. Setsaas, T.H., et al., How does human exploitation affect impala populations in protected and partially protected areas? - A case study from the Serengeti Ecosystem, Tanzania. Biological Conservation, 2007. **136**(4): p. 563-570.

28. Willcox, A.S. and D.M. Nambu, Wildlife hunting practices and bushmeat dynamics of the Banyangi and Mbo people of Southwestern Cameroon. Biological Conservation, 2007. **134**(2): p. 251-261.

29. Jachmann, H., Illegal wildlife use and protected area management in Ghana. Biological Conservation, 2008. **141**(7): p. 1906-1918.

30. Dunham, K.M., Detection of anthropogenic mortality in elephant Loxodonta africana populations: a long-term case study from the Sebungwe region of Zimbabwe. Oryx, 2008. **42**(1): p. 36-48.

31. Martin, E., C. Martin, and L. Vigne, Recent political disturbances in Nepal threaten rhinos: lessons to be learned. Pachyderm, 2008(45): p. 98-107.

32. Lee, T.M., N.S. Sodhi, and D.M. Prawiradilaga, Determinants of local people's attitude toward conservation and the consequential effects on illegal resource harvesting in the protected areas of Sulawesi (Indonesia). Environmental Conservation, 2009. **36**(2): p. 157-170.

33. Hayward, M.W., Bushmeat hunting in Dwesa and Cwebe Nature Reserves, Eastern Cape, South Africa. South African Journal of Wildlife Research, 2009. **39**(1): p. 70-84.

34. Owens, M.J. and D. Owens, Early age reproduction in female savanna elephants (Loxodonta africana) after severe poaching. African Journal of Ecology, 2009. **47**(2): p. 214-222.

35. Topp-Jørgensen, E., et al., Mammalian density in response to different levels of bushmeat hunting in the Udzungwa Mountains, Tanzania. Tropical Conservation Science, 2009. **2**(1): p. 70-87.

36. Brugiere, D. and B. Magassouba, Pattern and sustainability of the bushmeat trade in the Haut Niger National Park, Republic of Guinea. African Journal of Ecology, 2009. **47**(4): p. 630-639.

37. Waltert, M., B. Meyer, and C. Kiffner, Habitat availability, hunting or poaching: what affects distribution and density of large mammals in western Tanzanian woodlands? African Journal of Ecology, 2009. **47**(4): p. 737-746.

38. Rao, M., et al., Hunting, Livelihoods and Declining Wildlife in the Hponkanrazi Wildlife Sanctuary, North Myanmar. Environmental Management, 2010. **46**(2): p. 143-153.

39. Averbeck, C., et al., Hunting differentially affects mixed-sex and bachelor-herds in a gregarious ungulate, the impala (Aepyceros melampus: Bovidae). African Journal of Ecology, 2010. **48**(1): p. 255-264.

40. Bouche, P., et al., Has the final countdown to wildlife extinction in Northern Central African Republic begun? African Journal of Ecology, 2010. **48**(4): p. 994-1003.

41. Grey-Ross, R., C.T. Downs, and K. Kirkman, An assessment of illegal hunting on farmland in KwaZulu-Natal, South Africa: implications for oribi (Ourebia ourebi) conservation. South African Journal of Wildlife Research, 2010. **40**(1): p. 43-52.

42. Knapp, E.J., et al., A tale of three villages: Choosing an effective method for assessing poaching levels in western Serengeti, Tanzania. ORYX, 2010. **44**(2): p. 178-184.

43. Metzger, K.L., et al., Evaluating the protection of wildlife in parks: the case of African buffalo in Serengeti. Biodiversity and Conservation, 2010. **19**(12): p. 3431-3444.

44. Poilecot, P., Poaching and the elephant population in zakouma national park, Chad. Bois Et Forets Des Tropiques, 2010(303): p. 93-102.

45. Remis, M.J. and J.B. Kpanou, Primate and ungulate abundance in response to multi-use zoning and human extractive activities in a Central African Reserve. African Journal of Ecology, 2011. **49**(1): p. 70-80.

46. Kyale, D.M., S. Ngene, and J. Maingi, Biophysical and human factors determine the distribution of poached elephants in Tsavo East National Park, Kenya. Pachyderm, 2011(49): p. 48-60.

47. Gandiwa, E., Preliminary assessment of illegal hunting by communities adjacent to the northern Gonarezhou National Park, Zimbabwe. Tropical Conservation Science, 2011. **4**(4): p. 445-467.

48. Rao, M., et al., Hunting for a living: Wildlife trade, rural livelihoods and declining wildlife in the Hkakaborazi National Park, North Myanmar. Environmental Management, 2011. **48**(1): p. 158-167.

49. Linder, J.M. and J.F. Oates, Differential impact of bushmeat hunting on monkey species and implications for primate conservation in Korup National Park, Cameroon. Biological Conservation, 2011. **144**(2): p. 738-745.

50. Liu, F., et al., Human-wildlife conflicts influence attitudes but not necessarily behaviors: Factors driving the poaching of bears in China. Biological Conservation, 2011. **144**(1): p. 538-547.

51. Beyers, R.L., et al., Resource Wars and Conflict Ivory: The Impact of Civil Conflict on Elephants in the Democratic Republic of Congo - The Case of the Okapi Reserve. PLoS ONE, 2011. **6**(11).

52. Bouche, P., et al., Game over! Wildlife collapse in northern Central African Republic. Environmental Monitoring and Assessment, 2012. **184**(11): p. 7001-7011.

53. Martin, A., T. Caro, and M.B. Mulder, Bushmeat consumption in western Tanzania: A comparative analysis from the same ecosystem. Tropical Conservation Science, 2012. **5**(3): p. 352-364.

54. Jenks, K.E., J. Howard, and P. Leimgruber, Do Ranger Stations Deter Poaching Activity in National Parks in Thailand? Biotropica, 2012. **44**(6): p. 826-833.

55. Razafimanahaka, J.H., et al., Novel approach for quantifying illegal bushmeat consumption reveals high consumption of protected species in Madagascar. Oryx, 2012. **46**(4): p. 584-592.

56. Maingi, J.K., et al., Spatiotemporal patterns of elephant poaching in south-eastern Kenya. Wildlife Research, 2012. **39**(3): p. 234-249.

57. Kahler, J.S., G.J. Roloff, and M.L. Gore, Poaching Risks in Community-Based Natural Resource Management. Conservation Biology, 2013. **27**(1): p. 177-186.

58. Gandiwa, E., et al., Illegal hunting and law enforcement during a period of economic decline in Zimbabwe: A case study of northern Gonarezhou National Park and adjacent areas. Journal for Nature Conservation, 2013. **21**(3): p. 133-142.

59. Becker, M., et al., Evaluating wire-snare poaching trends and the impacts of by-catch on elephants and large carnivores. Biological Conservation, 2013. **158**: p. 26-36.

60. Martin, A. and T. Caro, Illegal hunting in the Katavi-Rukwa ecosystem. African Journal of Ecology, 2013. **51**(1): p. 172-175.

61. Watson, F., et al., Spatial patterns of wire-snare poaching: Implications for community conservation in buffer zones around National Parks. Biological Conservation, 2013. **168**: p. 1-9.

62. Mohsanin, S., et al., Assessing the threat of human consumption of tiger prey in the Bangladesh Sundarbans. Animal Conservation, 2013. **16**(1): p. 69-76.

63. Martin, E., C. Martin, and L. Vigne, Successful reduction in rhino poaching in Nepal. Pachyderm, 2013(54): p. 66-73.

64. Plumptre, A.J., et al., Efficiently targeting resources to deter illegal activities in protected areas. Journal of Applied Ecology, 2014. **51**(3): p. 714-725.

65. Booth, V.R. and K.M. Dunham, Elephant poaching in Niassa Reserve, Mozambique: population impact revealed by combined survey trends for live elephants and carcasses. Oryx, 2016. **50**(1): p. 94-103.

66. Ferreira, S.M., M. Pfab, and M. Knight, Management strategies to curb rhino poaching: Alternative options using a cost-benefit approach. South African Journal of Science, 2014. **110**(5-6).

67. Kimanzi, J.K., et al., Spatial distribution of snares in Ruma National Park, Kenya, with implications for management of the roan antelope Hippotragus equinus langheldi and other wildlife. Oryx, 2014. **FirstView**: p. 1-8.

68. Steinmetz, R., et al., Can community outreach alleviate poaching pressure and recover wildlife in South-East Asian protected areas? Journal of Applied Ecology, 2014. **51**(6): p. 1469-1478.

69. Wilfred, P. and A. MacColl, The pattern of poaching signs in Ugalla Game Reserve, western Tanzania. African Journal of Ecology, 2014. **52**(4): p. 543-551.

70. Vinks, M.A., et al., Testing the effects of anthropogenic pressures on a diverse African herbivore community. Ecosphere, 2020. **11**(3): p. e03067.

71. Romero‐Muñoz, A., et al., Habitat loss and overhunting synergistically drive the extirpation of jaguars from the Gran Chaco. Diversity and Distributions, 2019. **25**(2): p. 176-190.

72. Hema, E.M., et al., Population dynamics of medium and large mammals in a West African gallery forest area and the potential effects of poaching. Journal of Threatened Taxa, 2017. **9**(5): p. 10151-10157.

73. Fischer, F. and K.E. Linsenmair, Decreases in ungulate population densities. Examples from the Comoé National Park, Ivory Coast. Biological Conservation, 2001. **101**(2): p. 131-135.

74. Loginov, O. and I. Loginova, The snow leopard: the elusive ghost, but not yet lost, cat. A review of its conservation status in Siberia and Central Asia. International Journal of Environmental Studies, 2017. **74**(5): p. 903-914.

75. Mugume, S., et al., How do human activities influence the status and distribution of terrestrial mammals in forest reserves? Journal of mammalogy, 2015. **96**(5): p. 998-1004.

76. Irshad, N., et al., Distribution, abundance and diet of the Indian Pangolin (Manis crassicaudata). Animal Biology, 2015. **65**(1): p. 57-71.

77. Ghoddousi, A., et al., The decline of ungulate populations in Iranian protected areas calls for urgent action against poaching. Oryx, 2019. **53**(1): p. 151-158.

78. Castilho, L.C., et al., Hunting of mammal species in protected areas of the southern Bahian Atlantic Forest, Brazil. Oryx, 2019. **53**(4): p. 687-697.

79. Borgerson, C., The effects of illegal hunting and habitat on two sympatric endangered primates. International Journal of Primatology, 2015. **36**(1): p. 74-93.

80. Baamrane, M.A.A., et al., Demographic decline of the last surviving Moroccan dorcas gazelles Gazella dorcas massaesyla in M'Sabih Talaa Reserve, Morocco. Oryx, 2013. **47**(4): p. 578-583.

81. Kimanzi, J.K., R.A. Sanderson, and S.P. Rushton, Habitat suitability modelling and implications for management of roan antelopes in Kenya. African Journal of Ecology, 2014. **52**(1): p. 111-121.

**PART IV: LIST OF SPECIES ILLEGALLY HUNTED FROM 155 PROTECTED AREAS ACROSS 48 COUNTRIES IN FOUR CONTINENTS**

| **Species** | **Taxon** | **IUCN PA type** | **No.of Assessment** | **Cited threat** | **Continent** | **Pub. Year** |
| --- | --- | --- | --- | --- | --- | --- |
| Acinonyx jubatus | mammal | II, VI | 4 | poaching | Africa | 1995, 2011, 2013 |
| Aepyceros melampus | mammal | II, IV, VI | 14 | poaching | Africa | 1995, 06-7, 9-13, 20 |
| Ailurus fulgens | mammal | II, IV | 2 | poaching | Asia | 2010-11 |
| Alcelaphus buselaphus | mammal | Ia, II, IV, VI, V | 18 | poaching | Africa | 1995, 2000-1, 06, 9-10, 12-3, 17, 20 |
| Alouatta guariba guariba | mammal | Ia,II,III | 3 | poaching | South America | 2017 |
| Alouatta palliata | mammal | III | 1 | poaching | America | 2000 |
| Amblysomus hottentotus | mammal | IV | 2 | poaching and grazing | Africa | 2009 |
| Animal sp | mammal | II | 1 | poaching | Africa | 2002 |
| Arctictis binturong | mammal | II | 2 | poaching | Asia | 2005, 2012 |
| Arctocebus calabarensis | mammal | IV | 1 | poaching | Africa | 2007 |
| Arctonyx collaris | mammal | II | 2 | poaching | Asia | 2005 |
| Atelerix albiventris | mammal | II, IV | 2 | poaching | Africa | 1996, 2000 |
| Atherurus africanus | mammal | II, IV, V | 5 | poaching and legal hunting | Africa | 2000, 2006-7, 2009 |
| Atherurus macrourus | mammal | II | 1 | poaching | Asia | 2005 |
| Atilax paludinosus | mammal | IV | 3 | poaching | Africa | 2007, 2009 |
| Avahi occidentalis | mammal | II | 1 | poaching | Africa | 2003 |
| Axis axis | mammal | V | 1 | poaching | Asia | 2013 |
| Bat spp. | mammal | IV | 10 | poaching | Africa | 2007, 2009 |
| Bdeogale nigripes | mammal | IV | 1 | poaching | Africa | 2007 |
| Bos gaurus | mammal | II | 2 | poaching | Asia | 2012, 2014 |
| Brachyteles hypoxanthus | mammal | II, III | 3 | poaching | South America | 2017 |
| Bradypus torquatus | mammal | II, III | 3 | poaching | South America | 2017 |
| Budorcas taxicolor | mammal | II, IV | 2 | poaching | Asia | 2010-11 |
| Bunopithecus hoolock | mammal | II, IV | 2 | poaching | Asia | 2005, 2010 |
| Cabassous unicinctus | mammal | Ia | 3 | poaching | South America | 2017 |
| Callicebus melanochir | mammal | Ia, II, III | 3 | poaching | South America | 2017 |
| Callistomys pictus | mammal | Ia, II, III | 3 | poaching | South America | 2017 |
| Callithrix kuhlii | mammal | Ia | 3 | poaching | South America | 2017 |
| Canis adustus | mammal | IV | 1 | poaching | Africa | 2000 |
| Canis aureus | mammal | II | 1 | poaching | Asia | 2012 |
| Canis spp. | mammal | IV | 2 | poaching and grazing | Africa | 2009 |
| Capra aegagrus | mammal | II | 1 | poaching | Asia | 2017 |
| Capricornis sumatraensis | mammal | II, IV | 4 | poaching | Asia | 2005, 2010-12 |
| Catopuma temminckii | mammal | II, IV | 4 | poaching | Asia | 2005, 2010-12 |
| Cebus capucinus | mammal | III | 1 | poaching | America | 2000 |
| Cephalophus callipygus | mammal | II | 1 | poaching and logging | Africa | 2011 |
| Cephalophus dorsalis | mammal | II, Iv, V | 6 | poaching | Africa | 2000, 2006-7, 2011 |
| Cephalophus leucogaster | mammal | II | 1 | poaching and logging | Africa | 2011 |
| Cephalophus maxwelli | mammal | II | 1 | poaching | Africa | 2001 |
| Cephalophus natalensis | mammal | IV | 4 | poaching | Africa | 2006, 2015 |
| Cephalophus nigrifrons | mammal | II | 2 | poaching | Africa | 1997, 2011 |
| Cephalophus ogilbyi | mammal | II, IV | 3 | poaching | Africa | 2006-7 |
| Cephalophus rufilatus | mammal | Ia, II, V | 8 | poaching | Africa | 1996, 2001, 06, 9, 12, 17 |
| Cephalophus silvicultor | mammal | Ia, ii, IV | 6 | poaching | Africa | 2007, 2009, 2011-2 |
| Cephalophus spadix | mammal | IV | 1 | poaching | Africa | 2006 |
| Cephalophus spp. | mammal | Ib, IV, V, VI | 4 | poaching | Africa | 2000, 2009 |
| Ceratotherium simum | mammal | IV | 3 | poaching and grazing | Africa | 2009, 2011 |
| Cercocebus agilis | mammal | II | 1 | poaching | Africa | 2006 |
| Cercocebus torquatus | mammal | II | 4 | poaching | Africa | 2006-7, 2011 |
| Cercopithecus ascanius | mammal | II | 1 | poaching | Africa | 2006 |
| Cercopithecus erythrotis | mammal | II | 4 | poaching | Africa | 2006-7, 2011 |
| Cercopithecus mitis | mammal | Ib, IV, VI | 6 | poaching | Africa | 2006, 2009 |
| Cercopithecus mona | mammal | II, IV | 5 | poaching | Africa | 2006-7, 2011 |
| Cercopithecus neglectus | mammal | II | 1 | poaching | Africa | 2006 |
| Cercopithecus nictitans | mammal | II | 4 | poaching | Africa | 2006-7, 2011 |
| Cercopithecus pogonias | mammal | V | 3 | poaching and legal hunting | Africa | 2000, 2007, 2011 |
| Cerdocyon thous | mammal | Ia | 3 | poaching | South America | 2017 |
| Cervus elaphus | mammal | II | 1 | poaching | Asia | 2017 |
| Chlorocebus aethiops | mammal | Ia, II, IV | 10 | poaching | Africa | 2000, 06, 9, 12, 17 |
| Chlorocebus tantalus | mammal | V | 1 | poaching | Africa | 2017 |
| Civettictis civetta | mammal | II | 5 | poaching | Africa | 1996, 2000, 2006-7 |
| Colobus guereza | mammal | II | 4 | poaching | Africa | 2006, 2012 |
| Colobus polykomos | mammal | II | 1 | poaching | Africa | 2009 |
| Connochaetes taurinus | mammal | II | 7 | poaching | Africa | 1995, 2009-11, 13, 20 |
| Cricetomys emini | mammal | II, IV | 3 | poaching | Africa | 2006-7 |
| Cricetomys gambianus | mammal | II, IV | 3 | poaching | Africa | 2006, 2009 |
| Crocuta crocuta | mammal | II | 3 | poaching | Africa | 1996, 2011, 2013 |
| Crossarchus obscurus | mammal | IV | 1 | poaching | Africa | 2007 |
| Cryptoprocta ferox | mammal | II, VI | 3 | poaching | Africa | 2003, 2012 |
| Cuniculus paca | mammal | II, III | 3 | poaching | South America | 2017 |
| Cuon alpinus | mammal | II | 2 | poaching | Asia | 2005, 2012 |
| Damaliscus korrigum | mammal | II, VI | 2 | poaching | Africa | 1995, 2009 |
| Damaliscus lunatus | mammal | II, VI | 2 | poaching and water scarcity | Africa | 2012-13 |
| Dasyprocta leporina | mammal | II, III | 3 | poaching | South America | 2017 |
| Dasyprocta punctata | mammal | III | 1 | poaching | America | 2000 |
| Dasypus novemcintus | mammal | Ia | 3 | poaching | South America | 2017 |
| Dendrohyrax dorsalis | mammal | IV | 1 | poaching | Africa | 2007 |
| Diceros bicornis | mammal | II, IV, V, VI | 28 | poaching | Africa | 1981, 85,90,95-6,10-11,14 |
| Didelphis aurita | mammal | Ia, II, III | 3 | poaching | South America | 2017 |
| Didelphis marsupialis | mammal | III | 1 | poaching | America | 2000 |
| Dremomys lokriah | mammal | II | 1 | poaching | Asia | 2005 |
| Elephas maximus | mammal | II | 1 | poaching | Asia | 2012 |
| Equus burchellii | mammal | II | 2 | poaching | Africa | 1995, 2006 |
| Equus quagga | mammal | II, IV, VI | 10 | poaching and grazing | Africa | 2009, 2011-3, 2020 |
| Erethizon dorsatum | mammal | IV | 2 | poaching | Africa | 2015 |
| Erythrocebus patas | mammal | Ia, II, V | 6 | poaching | Africa | 2006, 09, 12, 17 |
| Eudorcas rufifrons | mammal | II | 1 | poaching | Africa | 1996 |
| Eudorcas thomsonii | mammal | II | 2 | poaching | Africa | 1995, 2006 |
| Eulemur albifrons | mammal | II | 1 | poaching | Africa | 2015 |
| Eulemur fulvus | mammal | II | 1 | poaching | Africa | 2003 |
| Eulemur rufifrons | mammal | II, VI | 2 | poaching | Africa | 2012 |
| Felis chaus | mammal | II | 1 | poaching | Asia | 2005 |
| Funisciurus pyrropus | mammal | IV | 1 | poaching | Africa | 2007 |
| Galago spp. | mammal | IV | 1 | poaching | Africa | 2007 |
| Galagoides spp. | mammal | IV | 1 | poaching | Africa | 2006 |
| Gazella dorcas massaesyla | mammal | VI | 1 | poaching and habitat loss | Africa | 2013 |
| Genetta genetta | mammal | II | 1 | poaching | Africa | 2013 |
| Genetta sp. | mammal | II, IV | 5 | poaching | Africa | 2000, 2006-7 |
| Genetta tigrina | mammal | IV | 2 | poaching and grazing | Africa | 2009 |
| Giraffa camelopardalis | mammal | Ia, II, VI | 15 | poaching | Africa | 1995-6, 2006, 2009-13 |
| Gorilla gorilla | mammal | II | 4 | poaching | Africa | 1996-7, 2003, 2011 |
| Helarctos malayanus | mammal | II, IV | 4 | poaching | Asia | 2005, 2010-12 |
| Helogale parvula | mammal | IV | 1 | poaching | Africa | 2000 |
| Herpestes urva | mammal | II | 1 | poaching | Asia | 2012 |
| Heterohyrax brucei | mammal | IV | 1 | poaching | Africa | 2000 |
| Hipotragus equinus | mammal | II, IV | 2 | poaching | Africa | 2013-4 |
| Hippopotamus amphibius | mammal | Ia, II, IV, VI, V | 11 | poaching | Africa | 1995-6, 2000, 06, 9-10, 12-3 |
| Hippotragus equinus | mammal | Ia, II, V, VI | 11 | poaching | Africa | 2000-1, 09-10, 12-3 |
| Hippotragus niger | mammal | II, IV, VI | 6 | poaching | Africa | 2000, 09, 11-3 |
| Hydrochoerus hydrochaeris | mammal | Ia | 3 | poaching | South America | 2017 |
| Hyemoschus aquaticus | mammal | II, IV | 3 | poaching | Africa | 2006-7 |
| Hylochoerus meinertzhageni | mammal | Ia, II, IV | 5 | poaching | Africa | 2006,12, 15 |
| Hylopetes spadiceus | mammal | II, IV | 2 | poaching | Asia | 2010-11 |
| Hyracoidea spp. | mammal | IV | 2 | poaching | Africa | 2006 |
| Hystrix africaeaustralis | mammal | IV | 3 | poaching | Africa | 2000, 02, 09 |
| Hystrix brachyura | mammal | II, IV | 5 | poaching | Asia | 2005, 11,12,14 |
| Hystrix cristata | mammal | II | 4 | poaching | Africa | 1996, 06, 9, 13 |
| Ichneumia albicauda | mammal | IV | 1 | poaching | Africa | 2000 |
| Ictonyx striatus | mammal | IV | 1 | poaching | Africa | 2000 |
| Kobus ellipsiprymnus | mammal | Ia, II, IV, VI, V | 16 | poaching | Africa | 2000-1,06,9-13,17, 20 |
| Kobus kob | mammal | Ia, II, V | 10 | poaching and floods | Africa | 1996, 2001, 06, 9-10, 12, 17 |
| Kobus vardonii | mammal | II, VI | 4 | poaching | Africa | 2009, 12-13, 20 |
| Leontopithecus chrysomelas | mammal | Ia, II, III | 3 | poaching | South America | 2017 |
| Lepilemur edwardsi | mammal | II | 1 | poaching | Africa | 2003 |
| Lepus saxatilis | mammal | II | 1 | poaching | Africa | 2009 |
| Lepus sp. | mammal | IV | 1 | poaching | Africa | 2000 |
| Lepus victoriae | mammal | II | 1 | poaching | Africa | 1996 |
| Litocranius walleri | mammal | II | 1 | poaching | Africa | 2006 |
| Lophocebus albigena | mammal | II | 1 | poaching and logging | Africa | 2011 |
| Loxodonta africana | mammal | Ia,b, II, IV, V, VI | 101 | poaching | Africa | 1980, 87,90,92-7,03, 06-14 |
| Lutra lutra | mammal | II, IV | 2 | poaching | Asia | 2010-11 |
| Lycaon pictus | mammal | II, VI | 3 | poaching and legal hunting | Africa | 2011, 13 |
| Macaca arctoides | mammal | II, IV | 3 | poaching | Asia | 2005, 10-11 |
| Macaca assamensis | mammal | II, IV | 2 | poaching | Asia | 2010-11 |
| Macaca fascicularis | mammal | II | 1 | poaching | Africa | 1996 |
| Macaca mulatta | mammal | II | 1 | poaching | Asia | 2005 |
| Macaca nemestrina | mammal | II | 1 | poaching | Asia | 2012 |
| Madoqua kirkii | mammal | II, IV | 2 | poaching | Africa | 2000, 06 |
| Mammal spp | mammal | Ia, II, IV, VI | 24 | poaching | Africa | 2007-8,13-14 |
| Mandrillus leucophaeus | mammal | II, IV | 4 | poaching | Africa | 2006-7, 11 |
| Manis crassicaudata | mammal | IV, V | 4 | poaching | Africa | 2015 |
| Manis gigantea | mammal | II | 3 | poaching | Africa | 1996, 09, 15 |
| Manis javanica | mammal | II, IV | 4 | poaching | Asia | 2005, 10-12 |
| Manis temminckii | mammal | IV | 1 | poaching | Africa | 2000 |
| Manis tetradactyla | mammal | IV | 1 | poaching | Africa | 2007 |
| Manis tricuspis | mammal | II, IV | 3 | poaching | Africa | 2006-7 |
| Martes flavigula | mammal | II, IV | 3 | poaching | Asia | 2005, 10-11 |
| Mazama americana | mammal | III | 1 | poaching | America | 2000 |
| Mazama gouazoubira | mammal | II, III | 1 | poaching | South America | 2017 |
| Mellivora capensis | mammal | II, IV | 2 | poaching | Africa | 1995, 2000 |
| Monachus monachus | mammal | II | 1 | poaching | Europe | 2004 |
| Moschus sp | mammal | II, IV | 2 | poaching | Asia | 2010-11 |
| Mungos mungo | mammal | IV | 1 | poaching | Africa | 2000 |
| Muntiacus feae | mammal | II | 1 | poaching | Asia | 2014 |
| Muntiacus muntjak | mammal | II, IV | 4 | poaching | Asia | 2005, 10, 12, 14 |
| Muntiacus putaoensis | mammal | II | 1 | poaching | Asia | 2005 |
| Muntjac sp | mammal | II | 1 | poaching | Asia | 2011 |
| Mustela strigidorsa | mammal | IV | 1 | poaching | Asia | 2010 |
| Mustelidae spp. | mammal | IV | 1 | poaching | Africa | 2006 |
| Naemorhedus baileyi | mammal | II | 1 | poaching | Asia | 2011 |
| Nandinia binotata | mammal | II, IV | 3 | poaching | Africa | 2006, 07 |
| Nanger granti | mammal | II | 2 | poaching | Africa | 1995, 2006 |
| Nasua narica | mammal | III |  | poaching | America | 2000 |
| Nasua nasua | mammal | Ia | 3 | poaching | South America | 2017 |
| Neofelis nebulosa | mammal | II, IV | 4 | poaching | Asia | 2005, 10-12 |
| Neotragus moschatus | mammal | Ib, II, IV, VI | 5 | poaching | Africa | 2000, 06,9, |
| Odocoileus virginianus | mammal | III | 1 | poaching | America | 2000 |
| Oreotragus oreotragus | mammal | II, IV | 2 | poaching | Africa | 2000, 13 |
| Orycteropus afer | mammal | II, IV, V | 4 | poaching | Africa | 2006, 9, 15 |
| Oryx beisa | mammal | II | 1 | poaching | Africa | 2006 |
| Otolemur crassicaudatus | mammal | IV | 1 | poaching | Africa | 2000 |
| Ourebia ourebi | mammal | Ia, II, V, VI | 9 | poaching | Africa | 2000-1,10,12 |
| Ovis vignei | mammal | II | 1 | poaching | Asia | 2017 |
| Paguma larvata | mammal | II, IV | 3 | poaching | Asia | 2005, 10-11 |
| Pan troglodytes | mammal | II, IV | 7 | poaching | Africa | 1996,06,7,9,11 |
| Panthera leo | mammal | II, IV, VI | 8 | poaching, legal hunting | Africa | 1995-6, 11,13 |
| Panthera onca | mammal | II, V | 4 | poaching | South America | 2018 |
| Panthera pardus | mammal | II, IV, VI | 6 | poaching | Africa | 1996, 2000, 11, 13 |
| Panthera tigris | mammal | IV, VI | 2 | poaching | Asia | 2002, 10 |
| Panthera uncia | mammal | Ia, II, IV, V | 10 | poaching | Asia, Europe | 2017 |
| Papio anubis | mammal | Ia, II, IV, V | 9 | poaching, disease | Africa | 1996,06,10,12,15,17 |
| Papio hamadryas | mammal | II, IV | 2 | poaching | Africa | 2000, 2009 |
| Papio ursinus | mammal | II | 1 | poaching | Africa | 2013 |
| Paradoxurus hermaphroditus | mammal | II | 1 | poaching | Asia | 2012 |
| Pardofelis marmorata | mammal | II | 1 | poaching | Asia | 2012 |
| Pecari tajacu | mammal | III | 1 | poaching | America | 2000 |
| Pedetes capensis | mammal | IV | 1 | poaching | Africa | 2000 |
| Phacochoerus aethiopicus | mammal | IV, VI | 2 | poaching, legal hunting | Africa | 2000, 2011 |
| Phacochoerus africanus | mammal | Ia, II, V, VI | 16 | poaching, disease, water scarcity | Africa | 1995-6, 2001, 06, 9-10,12-13, 17, 20 |
| Philatomba maxwelli | mammal | II | 1 | poaching | Africa | 2009 |
| Philantomba monticola | mammal | Ia, II, IV | 13 | poaching, illegal grazing | Africa | 2006-7, 09, 11-12, 15 |
| Piliocolobus preussi | mammal | II | 2 | poaching | Africa | 2006 |
| Poelagus marjorita | mammal | II | 1 | poaching | Africa | 2006 |
| Potamochoerus larvatus | mammal | Ib, II, IV,VI | 13 | poaching | Africa | 2000, 03, 06,09,11, 15 |
| Potos flavus | mammal | II, III | 2 | poaching | South America | 2017 |
| Priodontes maximus | mammal | II, III |  | poaching | South America | 2017 |
| Prionailurus bengalensis | mammal | II | 2 | poaching | Asia | 2005, 12 |
| Prionodon linsang | mammal | II, IV | 2 | poaching | Asia | 2010-11 |
| Prionodon pardicolor | mammal | II | 1 | poaching | Asia | 2005 |
| Procolobus pennantii | mammal | II | 1 | poaching and habitat loss | Africa | 2011 |
| Proechimys semispinosus | mammal | III | 1 | poaching | America | 2000 |
| Propithecus diadema | mammal | II, VI | 2 | poaching | Africa | 2012 |
| Propithecus verreauxi | mammal | II, VI | 2 | poaching | Africa | 2012 |
| Propithecus verreauxi coquereli | mammal | II | 1 | poaching | Africa | 2003 |
| Protoxerini spp. | mammal | IV | 1 | poaching | Africa | 2006 |
| Protoxerus stangeri | mammal | IV | 2 | poaching | Africa | 2000, 17 |
| Puma concolor | mammal | II, III | 3 | poaching | South America | 2017 |
| Raphicerus campestris | mammal | II | 1 | poaching | Africa | 2013 |
| Raphicerus sharpei | mammal | IV | 1 | poaching | Africa | 2000 |
| Rat spp. | mammal | IV | 1 | poaching | Africa | 2007 |
| Redunca redunca | mammal | Ia, II, IV, VI, V | 14 | Poaching and predation by hyena | Africa | 1985, 95, 2000, 06, 09-13, 17 |
| Rhinoceros unicornis | mammal | II, IV | 6 | poaching | Asia | 2008, 13 |
| Rhynchocyon spp. | mammal | IV | 1 | poaching | Africa | 2006 |
| Rondent spp. | mammal | Ia, II | 9 | poaching | Asia | 2009 |
| Rusa unicolor | mammal | II, IV | 5 | poaching | Asia | 2005, 10-12, 14 |
| Saguinus geoffroyi | mammal | III | 1 | poaching | America | 2000 |
| Sapajus xanthosternos | mammal | II, III | 1 | poaching | South America | 2017 |
| Sciurid spp. | mammal | II | 3 | poaching | Africa | 1996, 2006 |
| Sciurus granatensis | mammal | III | 1 | poaching | America | 2000 |
| Sus scrofa | mammal | Ia, II, IV | 15 | poaching | Asia | 2005, 09-12, 14,17 |
| Sylvicapra grimmia | mammal | IV | 15 | poaching | Africa | 2000-1,06,09-10,12-13,17 |
| Syncerus caffer | mammal | Ia,b, II, IV, V, VI | 30 | poaching | Africa | 1995-7, 2000-1, 06,09-13,17 |
| Tamandua mexicana | mammal | III | 1 | poaching | America | 2000 |
| Tamandua tetradactyla | mammal | II, III | 3 | poaching | South America | 2017 |
| Tapirus terrestris | mammal | II, III | 3 | poaching | South America | 2017 |
| Taurotragus derbianus | mammal | Ia, II | 5 | poaching and disease | Africa | 2010, 2012 |
| Taurotragus oryx | mammal | II, IV, VI | 9 | poaching | Africa | 1995, 06, 09, 11-13 |
| Tayassu pecari | mammal | II | 3 | poaching | South America | 2017 |
| Tenrec ecaudatus | mammal | II | 1 | poaching | Africa | 2003 |
| Thryonomys spp. | mammal | IV | 1 | poaching | Africa | 2006 |
| Thryonomys swinderianus | mammal | II | 6 | poaching | Africa | 1996, 06-7,09 |
| Trachypithecus phayrei | mammal | IV | 2 | poaching | Asia | 2010-11 |
| Trachypithecus pileatus | mammal | II | 1 | poaching | Asia | 2005 |
| Tragelaphus angasii | mammal | II | 1 | poaching | Africa | 2013 |
| Tragelaphus eurycerus | mammal | II | 2 | poaching | Africa | 2006,11 |
| Tragelaphus imberbis | mammal | II | 1 | poaching | Africa | 2006 |
| Tragelaphus scriptus | mammal | Ia, II, IV, VI, V | 22 | poaching | Africa | 1995, 97, 2000,01, 06-7,09-13,15,17 |
| Tragelaphus spekii | mammal | II, IV | 2 | poaching | Africa | 2000, 06 |
| Tragelaphus spp | mammal | II | 2 | poaching | Africa | 2006 |
| Tragelaphus strepsiceros | mammal | II, IV, VI | 8 | poaching | Africa | 2000, 09, 11-13 |
| Tragulus kanchil | mammal | II | 1 | poaching | Asia | 2012 |
| Ursus thibetanus | mammal | II, IV, VI | 4 | poaching | Asia | 2010-12 |
| Varecia rubra | mammal | II | 1 | Habitat loss | Africa | 2015 |
| Viverra megaspila | mammal | II | 1 | poaching | Asia | 2012 |
| Viverra zibetha | mammal | II | 1 | poaching | Asia | 2012 |
| Viverrid spp. | mammal | II | 1 | poaching | Africa | 2003 |
