# Supplementary material for: Global extent and drivers of mammal population declines in protected areas under illegal hunting pressure
Source: PLoS One. 2020 Aug 21;15(8):e0227163. doi: 10.1371/journal.pone.0227163 (PMC7446782; doi:10.1371/journal.pone.0227163)
Supplement: Attachment — Submitted filename: PONE-D-19-34271_R1_reviewer2020.pdf [file pone.0227163.s004.pdf]

PLOS ONE

Global extent and drivers of mammal population declines in protected areas under illegal hunting pressure

--Manuscript Draft--

**Manuscript Number:**	PONE-D-19-34271R1
**Article Type:**	Research Article
**Full Title:**	Global extent and drivers of mammal population declines in protected areas under illegal hunting pressure
**Short Title:**	Global review of the impact of poaching on wildlife in protected areas
**Corresponding Author:**	Alfan Abeid Rija, PhD<br>Sokoine University of Agriculture<br>Morogoro, TANZANIA, UNITED REPUBLIC OF
**Keywords:**	human development index; illegal harvest; land use change index; large mammals; population decline; protected areas
**Abstract:**	Illegal hunting is a persistent problem in many protected areas, but an overview of the extent of this problem and its impact on wildlife is lacking. We reviewed 40 years (1980-2020) of research across the globe to examine the spatial distribution of research and socio-ecological factors influencing population decline within protected areas under illegal hunting pressure. From 81 papers reporting 988 species/site combinations, 298 mammal species were reported to have been illegally hunted from 155 protected areas across 48 countries. Research in illegal hunting has increased substantially during the review period but also shows strong protected area and geographic biases towards strictly protected areas and African continent respectively, suggesting persistent poaching pressures on wild mammals in African heavily protected areas. Population declines were most frequent in countries with a low human development index particularly in strict protected areas and for species with a body mass over 100 kg. Overall, these findings provide evidence that illegal hunting is most likely to cause species declines of large-bodied animals in protected areas in resource-poor countries regardless of protected area conservation status (i.e. IUCN category). Given the mounting pressures of illegal hunting, increased investments in people's development and additional conservation efforts such as improving anti-poaching strategies and conservation resources in terms of improving funding and personnel directed at this problem are a growing priority.
**Order of Authors:**	Alfan Abeid Rija, PhD
	Rob Critchlow
	Chris Thomas
	Colin Beale
**Response to Reviewers:**	Details of our responses to each reviewer and the Journal Editor is found in the Response to Reviewers document included in the attachments
**Additional Information:**	
**Question**	**Response**
**Financial Disclosure**<br><br>Enter a financial disclosure statement that describes the sources of funding for the work included in this submission. Review the submission guidelines for detailed requirements. View published research articles from *PLOS ONE* for specific examples.	The main author is a Commonwealth scholar funded by the UK government. The sponsor-Commonwealth Scholarship Commission had no role in the design, data collection and analysis, decision to publish or preparation of the Manuscript.

This statement is required for submission and **will appear in the published article** if the submission is accepted. Please make sure it is accurate.<br><br>**Unfunded studies**<br>Enter: *The author(s) received no specific funding for this work.*<br><br>**Funded studies**<br>Enter a statement with the following details:<br>• Initials of the authors who received each award<br>• Grant numbers awarded to each author<br>• The full name of each funder<br>• URL of each funder website<br>• Did the sponsors or funders play any role in the study design, data collection and analysis, decision to publish, or preparation of the manuscript?<br>• **NO** - Include this sentence at the end of your statement: *The funders had no role in study design, data collection and analysis, decision to publish, or preparation of the manuscript.*<br>• **YES** - Specify the role(s) played.<br><br>* typeset	
**Competing Interests**<br><br>Use the instructions below to enter a competing interest statement for this submission. On behalf of all authors, disclose any competing interests that could be perceived to bias this work—acknowledging all financial support and any other relevant financial or non-financial competing interests.<br><br>This statement **will appear in the published article** if the submission is accepted. Please make sure it is accurate. View published research articles from *PLOS ONE* for specific examples.	The authors have declared that no competing interests exist

**NO authors have competing interests**<br><br>Enter: *The authors have declared that no competing interests exist.*<br><br>**Authors with competing interests**<br><br>Enter competing interest details beginning with this statement:<br><br>*I have read the journal's policy and the authors of this manuscript have the following competing interests: [insert competing interests here]*<br><br>* typeset	
**Ethics Statement**<br><br>Enter an ethics statement for this submission. This statement is required if the study involved:<br><br>• Human participants<br>• Human specimens or tissue<br>• Vertebrate animals or cephalopods<br>• Vertebrate embryos or tissues<br>• Field research<br><br>Write "N/A" if the submission does not require an ethics statement.<br><br>General guidance is provided below. Consult the submission guidelines for detailed instructions. **Make sure that all information entered here is included in the Methods section of the manuscript.**	Not applicable.

Powered by Editorial Manager® and ProduXion Manager® from Aries Systems Corporation

**Format for specific study types**<br><br>**Human Subject Research (involving human participants and/or tissue)**<br>• Give the name of the institutional review board or ethics committee that approved the study<br>• Include the approval number and/or a statement indicating approval of this research<br>• Indicate the form of consent obtained (written/oral) or the reason that consent was not obtained (e.g. the data were analyzed anonymously)<br><br>**Animal Research (involving vertebrate animals, embryos or tissues)**<br>• Provide the name of the Institutional Animal Care and Use Committee (IACUC) or other relevant ethics board that reviewed the study protocol, and indicate whether they approved this research or granted a formal waiver of ethical approval<br>• Include an approval number if one was obtained<br>• If the study involved *non-human primates*, add *additional details* about animal welfare and steps taken to ameliorate suffering<br>• If anesthesia, euthanasia, or any kind of animal sacrifice is part of the study, include briefly which substances and/or methods were applied<br><br>**Field Research**<br><br>Include the following details if this study involves the collection of plant, animal, or other materials from a natural setting:<br>• Field permit number<br>• Name of the institution or relevant body that granted permission	
**Data Availability**<br><br>Authors are required to make all data underlying the findings described fully available, without restriction, and from the time of publication. PLOS allows rare exceptions to address legal and ethical concerns. See the PLOS Data Policy and FAQ for detailed information.	Yes - all data are fully available without restriction

A Data Availability Statement describing where the data can be found is required at submission. Your answers to this question constitute the Data Availability Statement and **will be published in the article**, if accepted.<br><br>**Important:** Stating 'data available on request from the author' is not sufficient. If your data are only available upon request, select 'No' for the first question and explain your exceptional situation in the text box.<br><br>Do the authors confirm that all data underlying the findings described in their manuscript are fully available without restriction?	
**Describe where the data may be found in full sentences. If you are copying our sample text, replace any instances of XXX with the appropriate details.**<br><br>• If the data are **held or will be held in a public repository**, include URLs, accession numbers or DOIs. If this information will only be available after acceptance, indicate this by ticking the box below. For example: *All XXX files are available from the XXX database (accession number(s) XXX, XXX.*).<br>• If the data are all contained **within the manuscript and/or Supporting Information files**, enter the following: *All relevant data are within the manuscript and its Supporting Information files.*<br>• If neither of these applies but you are able to provide **details of access elsewhere**, with or without limitations, please do so. For example:<br><br>*Data cannot be shared publicly because of [XXX]. Data are available from the XXX Institutional Data Access / Ethics Committee (contact via XXX) for researchers who meet the criteria for access to confidential data.*<br><br>*The data underlying the results presented in the study are available from (include the name of the third party*	Data files are available, submitted with this revised Manuscript

and contact information or URL).<br>• This text is appropriate if the data are owned by a third party and authors do not have permission to share the data.<br><br>* typeset	
Additional data availability information:	

Manuscript
Click here to access/download;Manuscript;MANUSCRIPT.docx

Global extent and drivers of mammal population declines in protected areas under illegal hunting pressure

Short title: Global review of the impact of poaching on mammals in protected areas

Alfan A. Rija[1,2]*

[1]Department of Wildlife Management, Sokoine University of Agriculture, P.O. Box 3073, Chuo Kikuu, Morogoro, Tanzania

Email: al.rija10@gmail.com

[2]Rob Critchlow

[2]Department of Biology, University of York, Wentworth Way, YO10 5DD, United Kingdom

Email: Rob.critchlow@york.ac.uk

Chris D. Thomas[2]

[2]Department of Biology, University of York, Wentworth Way, YO10 5DD, United Kingdom

Email: chris.thomas@york.ac.uk

Colin M. Beale[2]

[2]Department of Biology, University of York, Wentworth Way, YO10 5DD, United Kingdom

Email: colin.beale@york.ac.uk

*Correspondence author: Dr Alfan A. Rija
Email: al.rija10@gmail.com
Tel: +255 753933886

Abstract

Illegal hunting is a persistent problem in many protected areas, but an overview of the extent of this problem and its impact on wildlife is lacking. We reviewed 40 years (1980-2020) of research across the globe to examine the spatial distribution of research and socio-ecological factors influencing population decline within protected areas under illegal hunting pressure. From 81 papers reporting 988 species/site combinations, 298 mammal species were reported to have been illegally hunted from 155 protected areas across 48 countries. Research in illegal hunting has increased substantially during the review period but also shows strong protected area and geographic biases towards strictly protected areas and African continent respectively, suggesting persistent poaching pressures on wild mammals in African heavily protected areas. Population declines were most frequent in countries with a low human development index particularly in strict protected areas and for species with a body mass over 100 kg. Overall, these findings provide evidence that illegal hunting is most likely to cause species declines of large-bodied animals in protected areas in resource-poor countries regardless of protected area conservation status (i.e. IUCN category). Given the mounting pressures of illegal hunting, increased investments in people's development and additional conservation efforts such as improving anti-poaching strategies and conservation resources in terms of improving funding and personnel directed at this problem are a growing priority.

Keywords: human development index, illegal harvest, land use change index, large mammals, population decline, protected areas

Introduction

Protected areas are the cornerstone of biodiversity conservation and have increased in number globally (covering at least 15% of global terrestrial land and 7% of ocean [1, 2]; to reach estimated conservation targets of protecting at least 17% of the global land area by 2020 [3, 4]. The effectiveness of protected areas is challenged by increasing pressures from management efficiency such as governance and resource deficiency [5-7], and anthropogenic pressures such as habitat loss and illegal hunting[8-10]. Consequently, many protected areas continue to experience wildlife declines [2, 9], suggesting that enlarging protected area network alone does not necessarily lead to conservation success when other efforts such as improved law enforcement, funding and regulation of land use change pressure outside are not considered [2]. Despite these challenges to protected area conservation, broad scale patterns on illegal hunting in protected areas and the consequences for species conservation remain poorly assessed, yet could help improve the effectiveness of protected areas and improve biodiversity conservation [10].

Anthropogenic threats to wildlife within and outside protected areas have been studied extensively including logging [11-13], hunting [14, 15] and encroachment through cropland conversion and livestock grazing [10, 12, 16]. These threats can have serious conservation and ecological implications. For example, recent assessments of wildlife abundance across tropical forests reported at least 40% decline in mammal distribution range due to hunting [17]. Also, mammal population declines by 80% and 30% have been reported across tropical forests caused by hunting and land use change respectively [9, 11] and potential loss of species interaction networks [18, 19]. Further, bushmeat hunting is a growing conservation problem across the

savanna biomes [20] and is a critical issue threatening several hundred thousand vertebrate species across the globe [18, 21]. Only few of those studies that investigate hunting impact account for protection status of an area affected by hunting and separate between legal and illegal hunting, which may be occurring together [9, 10]. This may potentially confound efforts needed to target illegal hunting pressures particularly in protected areas managed through law enforcement. Thus, research targeting the effectiveness of species conservation in relation to illegal hunting pressure is currently needed as protected areas remain the major stronghold of biodiversity and as important key refuge areas [10, 22].

At a local, individual protected area level, the drivers of illegal hunting are often known. This includes subsistence hunting to ensure food security and offtake for commercial gains [20, 23]. Species traits such as taste and size also influence hunting preference [9, 14, 24] but have scarcely been assessed on a large scale [11]. Increasing access to protected areas due to anthropogenic encroachment and land use change has also resulted in wildlife population declines, threatening protected areas effectiveness [2, 9] .

Mammals contribute the highest proportion to total biomass across forest and savanna landscapes making them highly sought after by illegal hunters [25-27]. Loss of mammals due to illegal hunting pressure has been related to substantial loss of important functional characteristics such as seed dispersal and regeneration and ecological interactions, thus endangering many ecological services that they support [2, 28, 29] and threatening humanity [30]. Therefore, due to their functional importance and the mounting threats on them, mammals become an excellent taxon to investigate their fate to improve conservation strategies in protected areas. Fortunately, the

distribution changes of mammals brought about by hunting are broadly known [17, 31] – but rarely focus on protected areas which should provide a refuge for all biodiversity [10].

Relatively few studies have quantified the relative contribution of individual threats to the overall pattern of population change and decline in Pas [8]. Such an assessment is required to identify strategies to improve PA effectiveness, such as where to target additional resources and which actions are most effective at enforcing existing regulations [32]. Here, we investigated spatial patterns and impact of illegal hunting on wild mammal populations within protected areas using a comprehensive database collated from 40 years of published literature. We built models that integrate both the outcome of illegal hunting pressure on species and the socio-ecological parameters to derive patterns of impacts on broader regional and global scales. Specifically, our aims were to:

I. Assess the extent and scale of research on illegal hunting of mammal populations in protected areas
II. Examine what factors are associated with mammal declines and their variations across species and protected areas and between geographic regions.
III. Identify current limitations in existing literature and propose recommendations for improving protected areas effectiveness in relation to illegal hunting of mammals.

Material and methods

Data collection

Literature searches were carried out in Web of Science, Google Scholar and Scopus between March 2014 and 2015 and in March 2020. Restricting the search period to 1980-2020, the search terms included: ‘illegal activity’ OR ‘illegal activities’ OR ‘illegal hunting’ OR poach*AND ‘protected area’ OR reserve OR ‘biodiversity outcome’. Full details of search terms can be found in the supplementary material. Results were screened based on criteria (i-iv below) to ensure each paper were related to both PAs and illegal mammal hunting:

i. Whether the research was done in a protected area and addressed issues of illegal hunting of mammals.

ii. If the research showed impact on species population within the protected area

iii. Only used primary data papers not meta-analyses or reviews.

iv. For results that returned more than one study covering the same protected area and year of data collection these were examined for relevance and only one that satisfied all criteria (i-iii) was included in the analysis.

From each paper we extracted information on PA location, threat types, study species, reason for illegal hunting, purpose of research and data collection method We recorded population trend (i.e. decline, no decline or unstated) for each PA/species combination and the reasons mentioned for such outcomes if not directly related to illegal hunting(e.g. timber extraction)Each species / site combination and associated variables became one row in the dataset, including the impact scores (1= species decline or 0 = no decline or NA = no reported impact for that species) and any covariate information (see Supplementary material- SM-data for the full database). The method arriving at the reported population trend status was recorded but not analyzed because most studies did not show the data used to arrive at a species outcome (e.g. decline), thus was difficult to tease apart whether the species decline was causal or correlative, a common problem in many meta-analysis studies [33].

Species body size is likely to influence mammal hunting risk [18]. We extracted body mass from the mammal database PanTHERIA (www.pantheria.org) and EltonTraits [34]. The body mass for two species (*Eulemur rufifrons* and *Gazella dorcas massaesyla*) were extracted from published literature. The WCMC IUCN Protected Planet database was used to identify the geographic location (latitude/longitude), and to assess whether legal status influences the outcome of species populations, we extracted the IUCN category of each PA [35].To explore the effect of legal status on species decline we grouped the PAs into two levels of protection: strict PAs (IUCN category I & II) and less strict PAs (categories III-IV). PAs in IUCN categories V-VI were excluded from analysis because they are not specifically designated for biodiversity conservation [36].

To assess whether population change reported was related to broad scale economic or social change, we extracted country-level human development (HDI) and agricultural land use change (ALC) indices from the UNDP and World Bank databases [37, 38]. ALC is an index of measure of the amount of land converted to agriculture and other human activities such as settlement. ALC is associated with habitat loss within protected areas due to encroaching on PAs [2, 12, 16] , and is also related to illegal hunting because farmers encroaching on PAs often engage in illegal hunting of animals [39]. We calculated the ALC over a decade period encompassing the times when research for the reviewed papers were conducted as most papers did not report the exact dates of data collection. We used HDI as a measure of socio-economic change and governmental effectiveness because HDI is a direct indicator of development.

Data analysis

We used each unique combination of species within an individual PA (i.e. species × PA) reported in a paper as a study as an individual data point, with therefore potentially several studies per paper (see data extraction in methods and supplementary materials). Using PA location and publication year, we assessed spatial and temporal patterns of illegal hunting in PAs.

To examine the effect of species traits and socio-economic variables on illegal mammal hunting in PAs, we used generalized linear mixed models (GLMM) with a binomial error term and logit link function using the statistical software R (version 3.6.3). For all models, we excluded records where the population outcome was unknown, and the response variable was whether a species had declined in that PA (1) or not (0). We built an initial global model incorporating six fixed factors: human development index (HDI), decadal agricultural land use change index (ALC), log species body mass , poaching type (commercial, subsistence, or combined), PA protection status (i.e. IUCN categories classified as strict and less strict) and continent (Africa/Asia/Central and South America) . Records from Europe were excluded from all analysis due to a low number of records (n =3). Because different species could relate to the same PA and country as studies from other papers at different times, we accounted for this by including country, paper and PA as random effects in all models and fitted the data using the 'glmer' function implemented in the R-package 'lme4'(24). We used a backwards stepwise removal of non-significant terms (with Chi-test) to evaluate the relative effect of each factor on the population decline. We obtained model confidence intervals around variables showing statistical significance in the minimum adequate model using the Wald-method [40]. Models of all analysis subsets were assessed using Akaike information criterion (AIC) to select the most parsimonious model after the stepwise removal of terms. We calculated a measure of variance explained by the models using the squared

correlation between the response variable and the predicted values. These pseudo R2 values should be interpreted with caution as it only shows the variance explained by the fixed effects in the final model.

The majority of illegal mammal hunting records were from Africa and Asia. We therefore built two additional models using the same structure as the global model, but restricted data to Africa and Asia respectively. Finally, because the savanna elephant Loxodonta africana, had a large percentage of records (19%) in the Africa dataset, reflecting the increasing concerns for elephant poaching and illegal ivory trade [41], we built two further models for Africa: one excluding savanna elephants and another only including savanna elephants.

Results

Extent and scale of research on illegal hunting of mammals in protected areas

Our searches found 2245 papers in total, 81 of which satisfied our selection criteria for inclusion in the review. The reviewed papers were from 48 countries and four continents, Africa, South America, Asia and Europe and covered 155 protected areas (Figure 1).

[INSERT FIGURE ONE HERE]

Figure 1. Spatial distribution of research on illegal hunting of mammals in 155 protected areas from 48 countries over four decades as collated in the literature. Black dots correspond to the centroid of a protected area where research for the reviewed papers was conducted.

There was an increasing trend in research on illegal hunting in protected areas with the number of publications increasing two-fold each decade since 1980. Types of study methods have also increased, particularly studies that have included use of ranger patrol data and interview techniques (Figure 2).

[INSERT FIGURE TWO HERE]

Figure 2. Number of studies per data collection method over 10-year periods. Numbers at top of bars indicate the cumulative number of publications. The majority (81%) of PAs studied were in the strictest IUCN categories (I-II).

Socio-economic and species traits influencing population decline in PAs

In the global model, **s**pecies body mass and PA strictness had a significant effect on the probability of species decline in PA. The probability of a population declines in a protected area increased with body mass and populations in stricter PAs (IUCN categories I-II) had a lower probability of decline compared to less strict PAs (IUCN categories III-IV) (Figure 3, Table 1; model1). When limiting analysis to Africa, populations were more likely to decline in countries with a lower HDI and for species with greater body mass (Figure 4, Table 1; model 2). Accounting for the influence of studies on illegal elephant hunting, results limited to Africa and excluding elephants showed that mammals with greater body mass were at greater risk of population decline in protected areas. (Table 1; model 3). Restricting analysis to African

elephants, showed that probability of population decline decreased with an increase in HDI (Table 1; model 4).

[INSERT FIGURE THREE HERE]

Figure 3. Effect of species body mass and protected area strictness on the probability of decline of mammal species from global records. Shaded area shows 95% confidence intervals. Circles represent raw data points.

[INSERT FIGURE FOUR HERE]

Figure 4. The probability of decline of mammals in Africa's protected areas threatened by poaching pressure showing population decline was strong in protected areas located in countries with low human development index and in species with larger body mass.

Results focusing on illegal mammals hunting in Asia showed that mammal populations in strictest PAs designated to protect biodiversity had a significantly greater probability of decline compared to mammal populations in less strict protected areas (Figure 5, Table 1; model 5).

[INSERT FIGURE FIVE HERE]

Figure 5. Probability of population decline for Asia's only protected areas indicating strong influence of protected area types. Higher decline of mammals was more likely in stricter than less-strict PAs.

Table 1: Summary of GLMMs fixed effects of socio-economic factors and species traits on the probability of mammal decline in PAs. Values in brackets represent 95% confidence intervals.

Models	1	2	3	4	5
	Global	Africa only	Africa- ex.elephant	Africa- elephant only	Asia only
Human development index		-8.97*		-15.80**	
		(-16.59, -1.35)		(-26.33, -5.28)	
Body mass	0.21**	0.34***	0.37***		

	(0.07, 0.34)	(0.16, 0.53)	(0.17, 0.58)		
PA strictness	-1.11*				1.77**
	(-1.95, -0.27)				(0.66, 2.88)
Intercept	1.00	3.53	-0.08	8.28***	-0.35
	(-0.22, 2.21)	(-0.11, 7.17)	(-1.32, 1.16)	(3.36, 13.14)	(-1.09, 0.39)
Observations	523	374	302	72	65
Pseudo R2	0.37	0.47	0.43	0.68	0.16
Significance					*p<0.05; **p<0.01; ***p<0.001

Discussion

Biodiversity in protected areas faces numerous anthropogenic threats [42]. We analyzed data published since 1980 to understand impacts of illegal hunting on species population decline in protected areas. There was strong geographic bias in research on illegal hunting within protected areas dominated by records in Africa and Asia. Globally, we found that illegally hunted mammals in strict PAs (IUCN categories I-II) had a lower probability of population decline compared to less strict PAs (IUCN categories III-IV). In contrast, we found illegally hunted

Asian mammals in strict PAs had a greater probability of population decline than less strict PAs. Human development index had a strong influence on likelihood of mammal declines in Africa, while larger bodied mammals were more likely to show population decline globally and in Africa.

The identification of correlations between human development indices and illegal hunting in this study supports a widely held view, e.g.[43, 44]but one that is often based on limited data [45]: that biodiversity decline is greater in relatively poor regions. Low human development scores could impact illegal hunting in two ways: firstly, poor people may tend to exploit species illegally from PAs because they have limited alternatives [46]. Secondly, poor countries have fewer resources to invest in PA conservation. Underfunding may result in increased illegal hunting, as well as other illegal activities such as agriculture. in PAs due to insufficient law enforcement [47]. Hilborn, and others [48] demonstrated that increased funding budgets for anti-poaching activities in the Serengeti National Park greatly reduced poaching pressures and led to the recovery of the buffalo population. However, increasing conservation funding may not necessarily result in improved conservation particularly when social and political constraints exist. For example, social and political unrest may increase rates of illegal hunting and encroachment in protected areas, reduce wildlife populations and thwart conservation efforts altogether [49, 50]. Our results provide evidence that poverty, in as much as it is measured by the HDI, may have significant negative impacts on species due to accelerated illegal hunting, whether that be because of increased external pressures on PAs or decreased policing and protection within strict PAs. These findings also provide evidence that supports assertions that illegal hunting and habitat loss are the major causes of continental-wide declines in megafauna in PAs across Africa and other tropical regions [2, 51]. Our model that included only African elephants also highlights the importance of low human development index in relation to illegal

ivory trade, and is consistent with recent analyses that this species is threatened with poaching and habitat loss across its habitat range in Africa [52, 53]. Our study highlights the need to consider human development issues more seriously to ensure effective conservation of biodiversity within existing protected areas.

Large bodied species are likely highly susceptible to decline because they have slow growth rates and so overharvesting is likely to cause population decline [54]. Low population growth rates in combination with multiple threats from illegal hunting and diseases are known to have significant impact on population persistence [55, 56]. By contrast, smaller mammals (with higher reproductive and growth rates) showed fewer declines and appeared to sustain harvest, though relatively few small species are the specific targets of poaching in PAs. An alternative explanation to the large mammal decline observed in our data could be that large mammals, due to their relatively higher protein content compared to smaller mammals they are mostly being targeted by illegal hunters for bushmeat sale. For example, a study in the Serengeti where illegal hunting is critical found that poachers prefer to hunt larger animals for their potential higher income returns [24].

The pattern of species declines across the network of protected areas is worrying and suggests that PA policing (including access to appropriate conservation information) and resources need to be improved. PA-specific information is important for understanding how illegal hunting varies spatially and across time and there is a need to be able to predict future trends and thereby possible future management strategies e.g. Critchlow and others [57]. Although at a global scale, mammals were less likely to decline by hunting in strict PAs, the opposite was true for Asian PAs This could be attributable to the habitat loss and poaching occurring inside these protected areas [58, 59], or the wider effects on animals that roam outside protected areas for parts of the year. These results are consistent with previous studies that have reported biodiversity decline

and loss within PAs in these regions e.g. Geldmann and others [60], Harrison [61], Laurance and others [62] and Gallego-Zamorano and others [17]. Our findings suggest that the effectiveness of Asian PAs requires urgent attention to improve biodiversity conservation across in continental Asia.

The geographical bias in the spatial distribution of research observed in these data is likely a consequence of interests among the researchers rather than being driven solely by the levels of illegal hunting in particular PAs or countries. However, the temporal and spatial patterns of research observed in this study provide insight into the extent of the problem of illegal hunting in PAs and therefore suggest that PAs are currently in need of new strategies to minimize impacts of illegal hunting pressure and to improve their conservation effectiveness [63]. To date, research effort has concentrated on quantifying the extent and impact of illegal hunting on focal species; in other words, documenting the problem. Far less information is available on which conservation management strategies (including human development and preventing illegal international trade, as well as within-PA activities) are most effective at reducing illegal hunting pressures. New research should focus on developing and testing new methods for reducing levels and impacts of illegal hunting on wild mammal species in PAs.

Conclusion

Tackling illegal hunting within protected areas remains a high conservation priority. Our results suggest that a combination of strategies may be required to reduce the extent of illegal hunting activities. We found that illegal hunting in poor countries often leads to population declines within PAs, suggesting that poverty alleviation may be an appropriate conservation strategy to reduce illegal hunting pressures [43]. The implication of this for local and national policies is

that more effort needs to be invested to improve the social and economic status of the human populations. This needs to work in tandem with increasing the effectiveness of traditional conservation activities to prevent illegal hunting; which may itself reduce the inclination of people to attempt future illegal hunting activities. Curbing external pressures to the PAs also needs improved efforts to prevent encroachment and other illegal activities such as crop farming within and squeezing of the PA borders. This will be urgent especially for Asia where stricter PAs are at greatest risk of losing their mammal populations.

Acknowledgements

We are grateful to the anonymous reviewers for the comments on earlier drafts of this manuscript. AAR was a commonwealth scholar funded by the U.K. government.

References

1. Tittensor, D.P., et al., *A mid-term analysis of progress toward international biodiversity targets.* Science, 2014. **346**(6206): p. 241-244.
2. Geldmann, J., et al., *A global-level assessment of the effectiveness of protected areas at resisting anthropogenic pressures.* Proceedings of the National Academy of Sciences, 2019. **116**(46): p. 23209-23215.
3. Visconti, P., et al., *Protected area targets post-2020.* Science, 2019. **364**(6437): p. 239-241.
4. CBD, *Convention on Biological Diversity. Aichi Biodiversity Targets. Available at https://www.cbd.int/sp/default.shtml. Accessed May 2015.* 2015.
5. Waldron, A., et al., *Targeting global conservation funding to limit immediate biodiversity declines.* Proceedings of the National Academy of Sciences, 2013. **110**(29): p. 12144-12148.
6. Schleicher, J., et al., *Conservation performance of different conservation governance regimes in the Peruvian Amazon.* Scientific reports, 2017. **7**(1): p. 1-10.
7. Venter, O., et al., *Bias in protected‐ area location and its effects on long‐ term aspirations of biodiversity conventions.* Conservation Biology, 2018. **32**(1): p. 127-134.
8. Geldmann, J., et al., *Effectiveness of terrestrial protected areas in reducing habitat loss and population declines.* Biological Conservation, 2013. **161**: p. 230-238.

9. Benítez-López, A., et al., *The impact of hunting on tropical mammal and bird populations.* Science, 2017. **356**(6334): p. 180-183.
10. Jones, K.R., et al., *One-third of global protected land is under intense human pressure.* Science, 2018. **360**(6390): p. 788-791.
11. Benitez-Lopez, A., et al., *Intact but empty forests? Patterns of hunting-induced mammal defaunation in the tropics.* PLoS biology, 2019. **17**(5).
12. de Lima, D.O., M.L. Lorini, and M.V. Vieira, *Conservation of grasslands and savannas: A meta-analysis on mammalian responses to anthropogenic disturbance.* Journal for Nature Conservation, 2018. **45**: p. 72-78.
13. Brodie, J.F., et al., *Correlation and persistence of hunting and logging impacts on tropical rainforest mammals.* Conservation Biology, 2015. **29**(1): p. 110-121.
14. Ripple, W.J., et al., *Bushmeat hunting and extinction risk to the world's mammals.* Royal Society open science, 2016. **3**(10): p. 160498.
15. Allan, J.R., et al., *Hotspots of human impact on threatened terrestrial vertebrates.* PLoS Biol, 2019. **17**(3): p. e3000158.
16. Maxwell, S.L., et al., *Biodiversity: The ravages of guns, nets and bulldozers.* Nature News, 2016. **536**(7615): p. 143.
17. Gallego- Zamorano, J., et al., *Combined effects of land use and hunting on distributions of tropical mammals.* Conservation Biology, 2020.
18. Ripple, W.J., et al., *Bushmeat hunting and extinction risk to the world's mammals.* Royal Society Open Science, 2016. **3**(10).
19. Edwards, D.P., et al., *Conservation of tropical forests in the anthropocene.* Current Biology, 2019. **29**(19): p. R1008-R1020.
20. van Velden, J., K. Wilson, and D. Biggs, *The evidence for the bushmeat crisis in African savannas: a systematic quantitative literature review.* Biological Conservation, 2018. **221**: p. 345-356.
21. Ripple, W.J., et al., *Are we eating the world's megafauna to extinction?* Conservation Letters, 2019. **12**(3): p. e12627.
22. Watson, J.E.M., et al., *The performance and potential of protected areas.* Nature, 2014. **515**(7525): p. 67-73.
23. Lindsey, P.A., et al., *The bushmeat trade in African savannas: Impacts, drivers, and possible solutions.* Biological Conservation, 2013. **160**: p. 80-96.
24. Ndibalema, V.G. and A.N. Songorwa, *Illegal meat hunting in serengeti: dynamics in consumption and preferences.* African Journal of Ecology, 2008. **46**(3): p. 311-319.
25. Ripple, W.J., et al., *Collapse of the world's largest herbivores.* Science Advances, 2015. **1**(4).
26. Lindsey, P.A., et al., *The bushmeat trade in African savannas: Impacts, drivers, and possible solutions.* Biological Conservation, 2013. **160**(0): p. 80-96.
27. Wilkie, D.S., et al., *The empty forest revisited.* Annals of the New York Academy of Sciences, 2011. **1223**(1): p. 120-128.
28. Abernethy, K.A., et al., *Extent and ecological consequences of hunting in Central African rainforests in the twenty-first century.* Philosophical Transactions of the Royal Society B: Biological Sciences, 2013. **368**(1625).
29. Galetti, M. and R. Dirzo, *Ecological and evolutionary consequences of living in a defaunated world.* Biological Conservation, 2013. **163**: p. 1-6.

30. Nasi, R., A. Taber, and N. Van Vliet, *Empty forests, empty stomachs? Bushmeat and livelihoods in the Congo and Amazon Basins.* International Forestry Review, 2011. **13**(3): p. 355-368.
31. Vinks, M.A., et al., *Testing the effects of anthropogenic pressures on a diverse African herbivore community*. Ecosphere, 2020. **11**(3): p. e03067.
32. Bruner, A.G., et al., *Effectiveness of Parks in Protecting Tropical Biodiversity.* Science, 2001. **291**(5501): p. 125-128.
33. Lortie, C.J., *Formalized synthesis opportunities for ecology: systematic reviews and meta‐ analyses.* Oikos, 2014. **123**(8): p. 897-902.
34. Wilman, H., et al., *EltonTraits 1.0: Species‐ level foraging attributes of the world's birds and mammals: Ecological Archives E095‐ 178.* Ecology, 2014. **95**(7): p. 2027-2027.
35. WCMCPP, *WCMC IUCN Protected Planet Database. Available at http://www.protectedplanet.net/. Accessed between Feb-March 2015.* 2015.
36. Santini, L., S. Saura, and C. Rondinini, *Connectivity of the global network of protected areas.* Diversity and Distributions, 2016. **22**(2): p. 199-211.
37. UNDP, *Human Development Reports 2014. Available at http://hdr.undp.org/en/content/human-development-index-hdi. Accessed in February 2015.* 2015.
38. WB, *The World Bank Datasets. Available at http://data.worldbank.org/indicator/AG.LND.AGRI.ZS. Accessed in March 2015.* 2015.
39. Bennett, E.L. and M.T. Gumal, *The interrelationships of commercial logging, hunting and wildlife in Sarawak.* The cutting edge, conserving wildlife in logged forests. Columbia University Press, New York, New York, USA, 2001: p. 359-374.
40. Bolker, B.M., et al., *Generalized linear mixed models: a practical guide for ecology and evolution.* Trends in Ecology & Evolution, 2009. **24**(3): p. 127-135.
41. Wasser, S.K., et al., *Genetic assignment of large seizures of elephant ivory reveals Africa's major poaching hotspots.* Science, 2015. **349**(6243): p. 84-7.
42. Butchart, S.H.M., et al., *Global Biodiversity: Indicators of Recent Declines.* Science, 2010. **328**(5982): p. 1164-1168.
43. Bennett, E.L., et al., *Hunting for Consensus: Reconciling Bushmeat Harvest, Conservation, and Development Policy in West and Central Africa.* Conservation Biology, 2007. **21**(3): p. 884-887.
44. Adams, W.M., et al., *Biodiversity Conservation and the Eradication of Poverty.* Science, 2004. **306**(5699): p. 1146-1149.
45. Nellemann, C., et al., *The Environmental Crime Crisis – Threats to Sustainable Development from Illegal Exploitation and Trade in Wildlife and Forest Resources. A UNEP Rapid Response Assessment. United Nations Environment Programme and GRID-Arendal, Nairobi and Arendal.* 2014.
46. Brashares, J.S., et al., *Economic and geographic drivers of wildlife consumption in rural Africa.* Proceedings of the National Academy of Sciences, 2011. **108**(34): p. 13931-13936.
47. Keane, A., et al., *The sleeping policeman: understanding issues of enforcement and compliance in conservation.* Animal Conservation, 2008. **11**(2): p. 75-82.
48. Hilborn, R., et al., *Effective enforcement in a conservation area.* Science, 2006. **314**(5803): p. 1266-1266.

49. de Merode, E., et al., *Status of elephant populations in Garamba National Park, Democratic Republic of Congo, late 2005.* Pachyderm, 2007(42): p. 52-57.
50. Bouche, P., et al., *Game over! Wildlife collapse in northern Central African Republic.* Environmental Monitoring and Assessment, 2012. **184**(11): p. 7001-7011.
51. Craigie, I.D., et al., *Large mammal population declines in Africa's protected areas.* Biological Conservation, 2010. **143**(9): p. 2221-2228.
52. Wittemyer, G., et al., *Illegal killing for ivory drives global decline in African elephants.* Proceedings of the National Academy of Sciences of the United States of America, 2014. **111**(36): p. 13117-13121.
53. Maisels, F., et al., *Devastating Decline of Forest Elephants in Central Africa.* PLoS ONE, 2013. **8**(3): p. e59469.
54. Purvis, A., et al., *Predicting Extinction Risk in Declining Species.* Proceedings: Biological Sciences, 2000. **267**(1456): p. 1947-1952.
55. Woodroffe, R. and J.R. Ginsberg, *Edge Effects and the Extinction of Populations Inside Protected Areas.* Science, 1998. **280**(5372): p. 2126-2128.
56. Cardillo, M., et al., *Multiple Causes of High Extinction Risk in Large Mammal Species.* Science, 2005. **309**(5738): p. 1239-1241.
57. Critchlow, R., et al., *Spatiotemporal trends of illegal activities from ranger-collected data in a Ugandan national park.* Conservation Biology, 2015. **29**(5): p. n/a-n/a.
58. Curran, L.M., et al., *Lowland Forest Loss in Protected Areas of Indonesian Borneo.* Science, 2004. **303**(5660): p. 1000-1003.
59. Corlett, R.T., *The Impact of Hunting on the Mammalian Fauna of Tropical Asian Forests.* Biotropica, 2007. **39**(3): p. 292-303.
60. Geldmann, J., et al., *Effectiveness of terrestrial protected areas in reducing habitat loss and population declines.* Biological Conservation, 2013. **161**(0): p. 230-238.
61. Harrison, R.D., *Emptying the Forest: Hunting and the Extirpation of Wildlife from Tropical Nature Reserves.* BioScience, 2011. **61**(11): p. 919-924.
62. Laurance, W.F., et al., *Averting biodiversity collapse in tropical forest protected areas.* Nature, 2012. **489**(7415): p. 290-294.
63. Watson., J.E.M., et al., *Bolder science needed now for protected areas.* Conservation Biology, 2016. **30**(2): p. 243-248.

 Click here to access/download;Figure;LIST OF FIGURES_NEW.docx

LIST OF FIGURES

FIGURE ONE

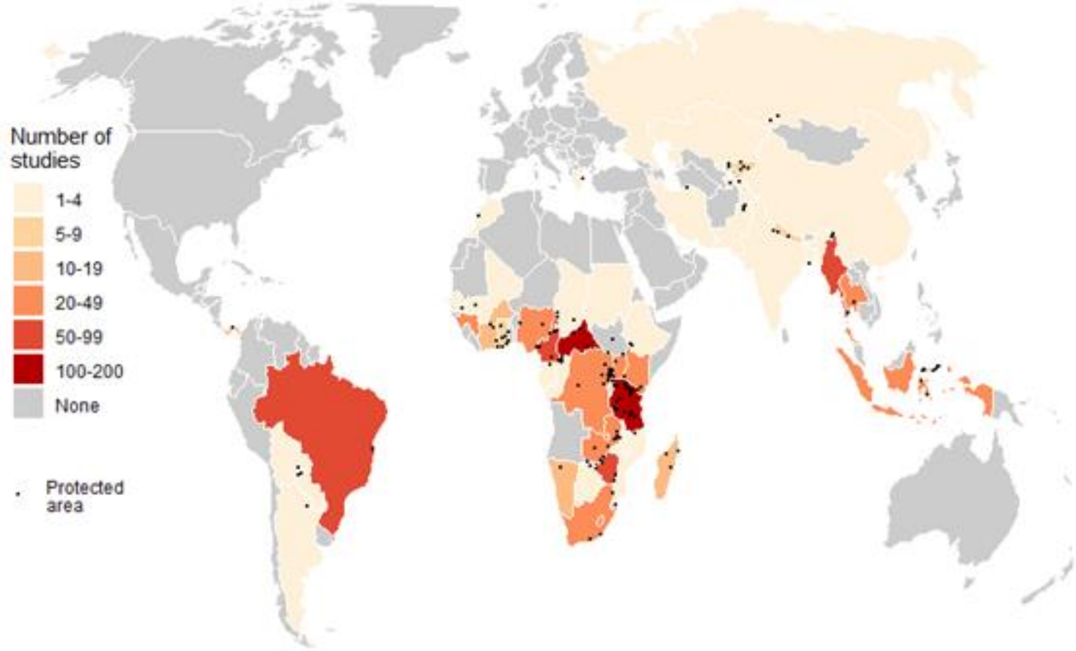

FIGURE TWO

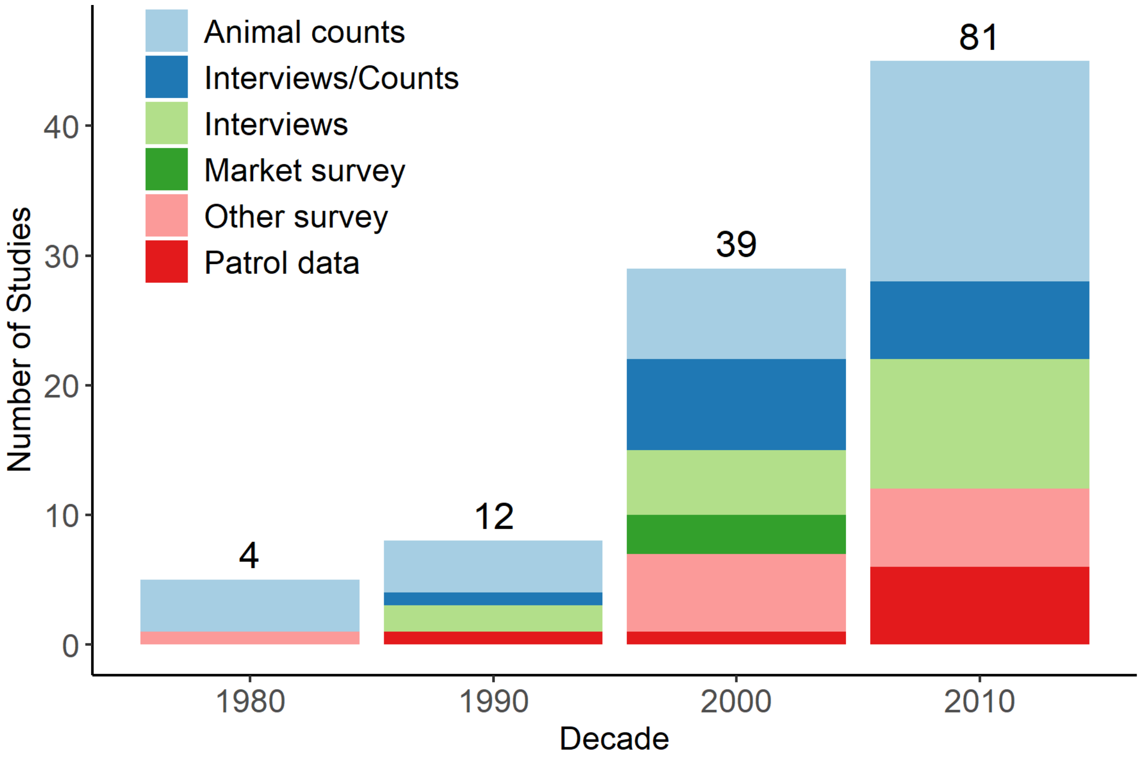

Animal counts
Interviews/Counts
Interviews
Market survey
Other survey
Patrol data
Number of Studies
0
10
20
30
40
4
12
39
81
1980
1990
2000
2010
Decade

FIGURE THREE

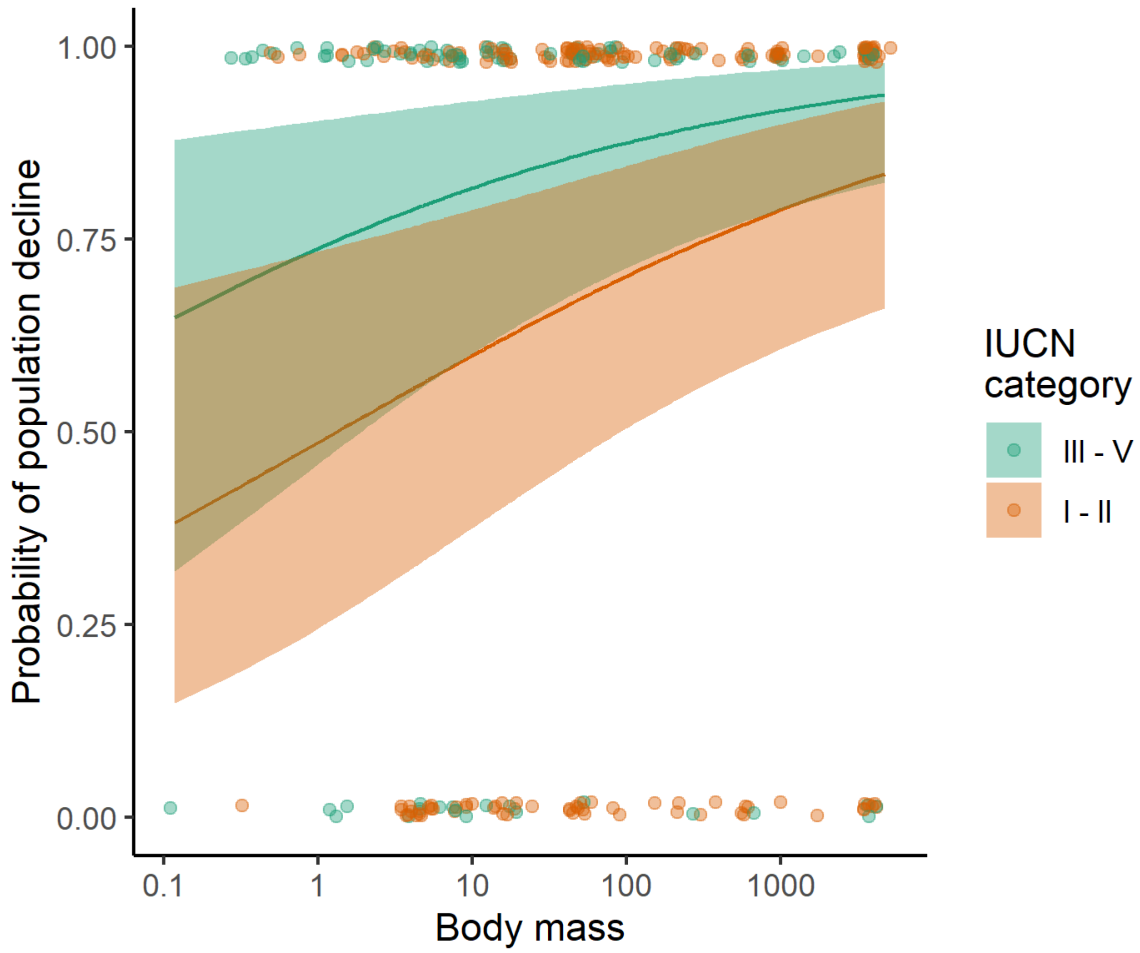

FIGURE FOUR

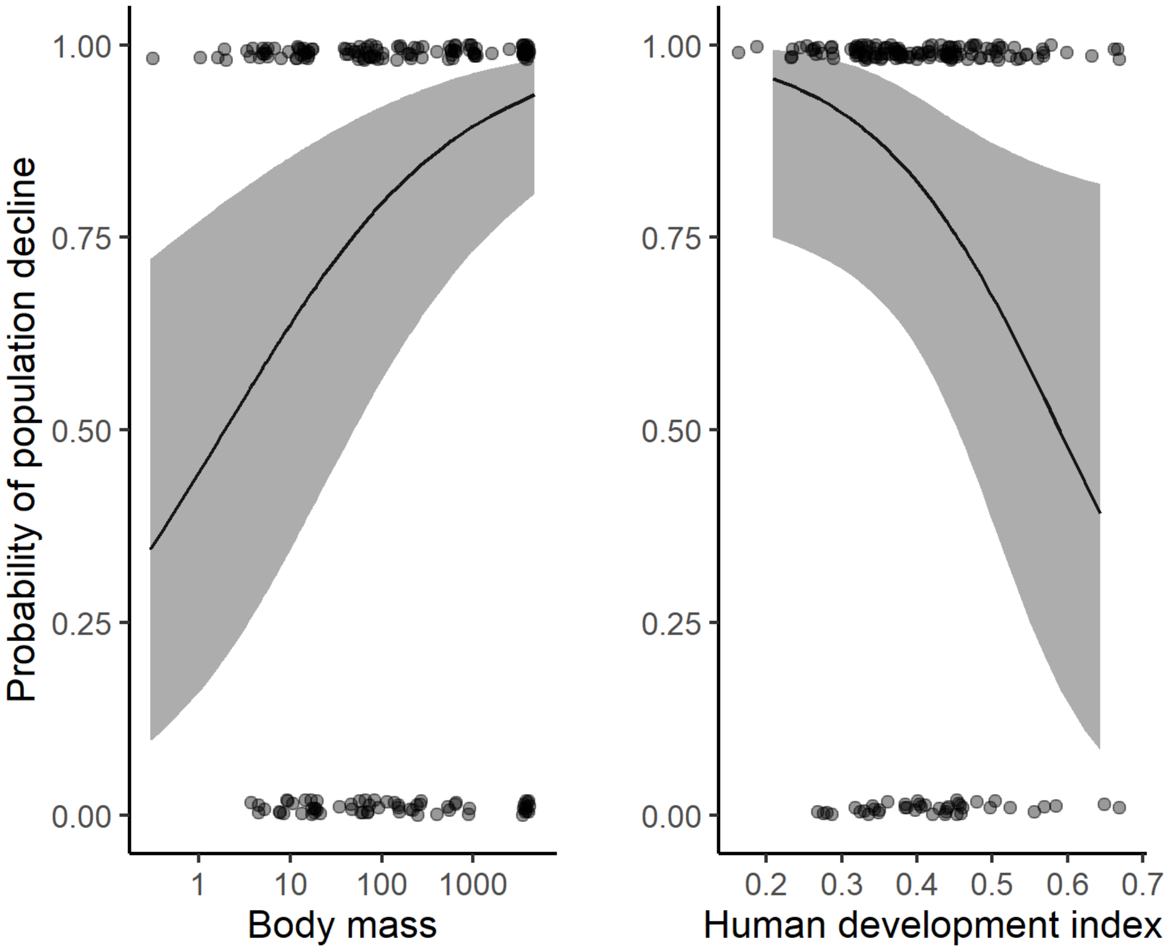
Probability of population decline
1.00
0.75
0.50
0.25
0.00
1
10
100
1000
Body mass
0.2
0.3
0.4
0.5
0.6
0.7
Human development index

FIGURE FIVE

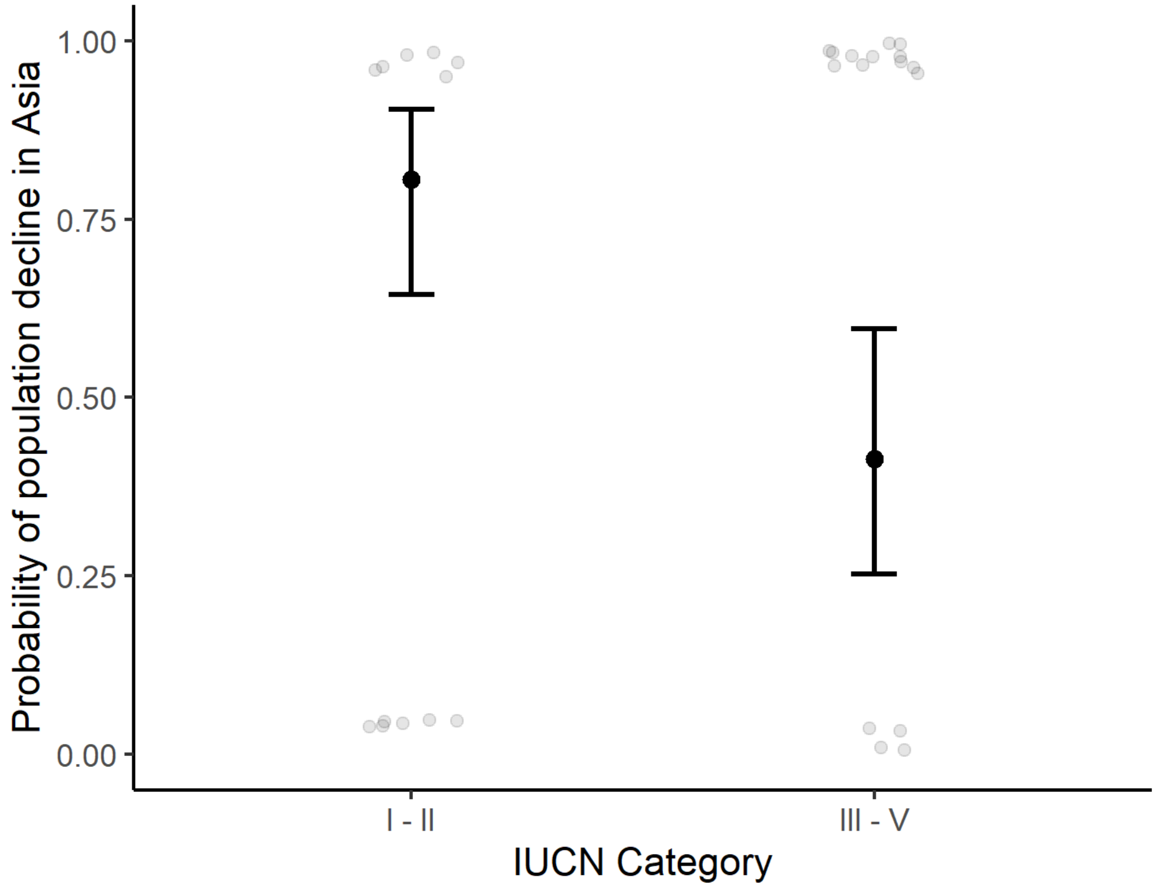

Probability of population decline in Asia
1.00
0.75
0.50
0.25
0.00
I - II
III - V
IUCN Category

Click here to access/download
Supporting Information
Meta data_mammal_database_100420.csv

Click here to access/download
Supporting Information
Search term and list of reviewed papers.docx

Click here to access/download
Supporting Information
mammals_database_100420.csv

Click here to access/download
Supporting Information
Supplementary Materials.docx

Revised Manuscript with Track Changes

~~Spatial distribution and research trend of illegal activities and the factors associated with wild mammal population declines in protected areas~~Global extent and drivers of ~~Socio-economic and Species traits determine~~ mammal population declines in protected areas under illegal hunting pressure

Short title: Global review of the impact of poaching on mammals ~~wildlife~~ in protected areas

Alfan A. Rija[1,2]*

[1]Department of Wildlife Management, Sokoine University of Agriculture, P.O. Box 3073, Chuo Kikuu, Morogoro, Tanzania

Email: al.rija10@gmail.com

[2]Rob Critchlow

[2]Department of Biology, University of York, Wentworth Way, YO10 5DD, United Kingdom

Email: Rob.critchlow@york.ac.uk

Chris D. Thomas[2]

[2]Department of Biology, University of York, Wentworth Way, YO10 5DD, United Kingdom

Email: chris.thomas@york.ac.uk

Colin M. Beale[2]

[2]Department of Biology, University of York, Wentworth Way, YO10 5DD, United Kingdom

Email: colin.beale@york.ac.uk

*Correspondence author: Dr Alfan A. Rija
Email: al.rija10@gmail.com
Tel: +255 753933886

Abstract

Illegal hunting~~activities~~ ~~is~~are ~~–~~is a persistent problem in many protected areas, but an overview of the extent of this problem and its impact on wildlife is lacking. We review 40~~35~~ years (1980-~~91914~~2020~~19~~19~~14~~) of research across the globe to examine the spatial distribution of research and socio-ecological factors influencing population decline within protected areas under illegal hunting ~~activities~~ pressure. From 81~~910192~~ papers reporting 988~~1048~~ species/site combinations~~, more than 350~~ ~~for~~ 298 mammal species ~~that~~~~comprising mammals, reptiles, birds, fishes and molluscs~~ were reported to have been ~~extracted~~ illegally hunted from 155~~46~~ protected areas across 48 countries ~~areas across four continents~~. Research in illegal hunting~~activities~~ has increased substantially during the review period but also shows strong protected area ~~taxonomic~~ and geographic biases towards strictly protected areas~~large wild mammals~~ and African continent respectively, suggesting persistent poaching pressures on wild mammals in African heavily protected areas. Population declines were most frequent ~~i) where there was commercial poaching as opposed to subsistence poaching alone, ii)~~ in countries with a low human development index particularly in strict protected areas and ~~iii)~~ for species with a body mass over 100 kg. ~~Habitat loss associated with greater land use change had an additional significant impact on population decline, particularly in the less-strict categories (IUCN III–VVI) of protected area across the continents.~~ Overall, these findings provide evidence that illegal hunting~~activities pressure~~ is ~~are~~ most likely to cause species declines of large-bodied animals in protected areas in resource-poor countries regardless of protected area conservation status (i.e. IUCN category). Given the mounting pressures of illegal hunting~~activities~~, increased investments in people's development and additional conservation efforts~~effort~~ such as improving anti-poaching strategies and conservation resources in terms of improving funding and personnel directed at this problem are~~is~~ a growing priority.

Commented [1]: this is now 88 just 'mammal' papers

Keywords: human development index, illegal harvest, land use change index, large mammals~~megafauna~~, population decline, protected areas

Introduction

Protected areas are the cornerstone of biodiversity conservation and have increased in number globally (covering at least 15% of global terrestrial land and 7% of ocean- [1, 2]~~Tittensor et al 2014; Geldmann et al. 2019)~~; to reach estimated conservation targets of protecting at least 17% of the global land area by 2020 [3, 4]~~(CBD, 2015, Visconti et al. 2019)~~. The effectiveness of protected areas is challenged by increasing pressures from management efficiency such as governance and resource deficiency [5-7]~~(Waldron et al.2013, Schleicher et al. 2017, Venter et al. 2018)~~, and anthropogenic pressures such as habitat loss and illegal hunting [8-10]~~(Geldmann et al. 2013; Benitez-Lopez et al. 2017, Jones et al 2018)~~. Consequently, many protected areas continue to experience wildlife declines [2, 9]~~(Benitez-Lopez et al. 2017, Geldmann et al. 2019)~~, suggesting that enlarging protected area network alone does not necessarily lead to conservation success when other efforts such as improved law enforcement, funding and regulation of land use change pressure outside are not considered [2]~~(Geldmann et al. 2019)~~. Despite these challenges to protected area conservation, broad scale patterns on illegal hunting in protected areas and the consequences for species conservation remain poorly assessed, yet could help improve the effectiveness of protected areas and improve biodiversity conservation [10]~~(Jones et al. 2018)~~.

Anthropogenic threats to wildlife within and outside protected areas have been studied extensively including logging [11-13](Brodie et al. 2014, Benitez-Lopez et al. 2019 and de Lima et al. 2018), hunting [14, 15](Ripple et al. 2016, Allan et al.2019) and encroachment through cropland conversion and livestock grazing [10, 12, 16](de Lima et al. 2018, Maxwell et al. 2016, Jones et al. 2018). These threats can have serious conservation and ecological implications. For example, recent assessments of wildlife abundance across tropical forests reported at least 40% decline in mammal distribution range due to hunting [17](Gallego-Zamorano et al. 2020). Also, mammal population declines by 80% and 30% have been reported across tropical forests caused by hunting and land use change respectively [9, 11](Benitez-Lopez et al. 2017, 2019) and potential loss of species interaction networks [18, 19](Ripple et al. 2016, Edwards et al. 2019). Further, bushmeat hunting is a growing conservation problem across the savanna biomes [20](van Velden et al.2018) and is a critical issue threatening several hundred thousand vertebrate species across the globe [18, 21](Ripple et al. 2016; 2019). Only few of those studies that investigate hunting impact account for protection status of an area affected by hunting and separate between legal and illegal hunting, which may be occurring together [9, 10](Benitez-Lopez et al. 2017, Jones et al. 2018). This may potentially confound efforts needed to target illegal hunting pressures particularly in protected areas managed through law enforcement. Thus, research targeting the effectiveness of species conservation in relation to illegal hunting pressure is currently needed as protected areas remain the major stronghold of biodiversity and as important key refuge areas [10, 22](Watson et al. 2014, Jones et al. 2018).

At a local, individual protected area level, the drivers of illegal hunting are often known. This includes subsistence hunting to ensure food security and offtake for commercial gains

[20, 23](Lindsey et al. 2013, van Velden et al. 2018). Species traits such as taste and size also influence hunting preference [9, 14, 24](Ndibalema & Songorwa 2008, Ripple et al. 2016, Benitez-Lopez et al. 2017) but have scarcely been assessed on a large scale [11](Benitez-Lopez et al. 2019). Increasing access to protected areas due to anthropogenic encroachment and land use change has also resulted in wildlife population declines, threatening protected areas effectiveness [2, 9](Benitez-Lopez et al. 2017, Geldmann et al. 2019).

Mammals contribute the highest proportion to total biomass across forest and savanna landscapes making them highly sought after by illegal hunters (Ripple et al. 2015, Wilkie et al. 2011, Lindsey et al. 2013). Loss of mammals due to illegal hunting pressure has been related to substantial loss of important functional characteristics such as seed dispersal and regeneration and ecological interactions, thus endangering many ecological services that they support (Galleti & Dirzo, 2013; Abernethy et al. 2013, Ripple et al. 2019) and threatening humanity (Nasi et al. 2011). Therefore, due to their functional importance and the mounting threats on them, mammals become an excellent taxon to investigate their fate to improve conservation strategies in protected areas. Fortunately, the distribution changes of mammals brought about by hunting are broadly known (Vinks et al. 2020; Gallego-Zamorano et al. 2020) – but rarely focus on protected areas which should provide a refuge for all biodiversity (Jones et al. 2018).

Relatively few studies have quantified the relative contribution of individual threats to the overall pattern of population change and decline in PAs (Geldmann et al. 2013). Such an assessment is required to identify strategies to improve PA effectiveness, such as where to

target additional resources and which actions are most effective at enforcing existing regulations (Bruner et al. 2001). Here, we investigate spatial patterns and impact of illegal hunting on wild mammal populations within protected areas using a comprehensive database collated from 40 years of published literature. We build models that integrate both the outcome of illegal hunting pressure on species and the socio-ecological parameters to derive patterns of impacts on broader regional and global scales. Specifically, our aims were to:

I. **Assess the extent and scale of research on illegal hunting of mammal populations in protected areas**
II. **Examine what factors are associated with mammal declines and their variations across species and protected areas and between geographic regions.**
III. **Identify current limitations in existing literature and propose recommendations for improving protected areas effectiveness in relation to illegal hunting of mammals.**

Improving the spatial coverage of protected area (PA) network is increasingly viewed as a global biodiversity conservation priority (1, 2). The global land area with legal protection for conservation has increased from 3.5% in 1985 (3) to 13% recently (4) and looks set to increase further as countries aim to fulfil the Aichi target of protecting 17% of terrestrial land by 2020 (5). Despite this increase and the investment made in protected areas (6), biodiversity loss is perceived to be continuing even in PAs(Butchart *et al.*, 2010; Mora & Sale, 2011). For example, Craigie, Baillie (7) reported continent-wide population declines of large mammals across several of Africa's protected areas. To allocate conservation resources efficiently, it is important to understand the scale and trends of illegal activities. Most studies, e.g. Mitchell (8), Wright, Zeballos (9), Leader-Williams, Albon (10) have focused on a single threat, on a single protected area type or region, and/or over short time periods(Jachmann, 2008a; Kimanzi *et al.*, 2014;

Plumptre *et al.*, 1997). These provide crucial PA-specific data, but information on illegal activities and their impact at broader spatial and temporal scales is sparse, making tackling illegal activities difficult. Here we review the site-specific literature to assess the global and regional patterns and impacts of illegal activities on species in protected areas and to provide information on the factors associated with population decline that may help improve the conservation of existing protected areas.

Anthropogenic threats reported from within PAs include hunting/poaching, logging, settlement, cultivation, livestock grazing etc. Each threat can reduce the ability of PAs to preserve biodiversity effectively (11)(Craigie *et al.*, 2010; Curran *et al.*, 2004; Pfeifer *et al.*, 2012). Moreover, the ability of PAs to protect species can be influenced by factors beyond their boundaries. For example, human-animal conflict has caused widespread mortality of carnivore species at and outside reserve borders, jeopardizing the persistence of populations inside protected areas, particularly for species with large home ranges that encompass both protected and unprotected land (12). At a larger scale, poaching of migratory species during periods when they are outside PAs has been reported as among the major threats imperiling the long-term conservation of species such as saiga (*Saiga tatarica*) and wildebeest (*Connochaetes taurinus*) antelopes, in Asia and East Africa respectively(13, 14). The ability of populations to sustain and recover from illegal activities within and surrounding PAs will depend on the type and level of activity, combined with the biological characteristics of the species affected by those activities. For example, selective poaching of males has been associated with the reproductive collapse and population decline of saiga (13)(Cardillo *et al.*, 2005; Sala *et al.*, 2000), and species with low rates of reproduction and growth may be particularly prone to population decline and extinction (15).

While the studies above provide useful accounts of the threats facing PAs, relatively few of them quantify the relative contribution of individual threats to the overall pattern of population change and decline in PAs (16). Such an assessment is required to identify strategies to improve PA performance, such as where to target additional resources and which actions are most effective at enforcing existing regulations (17). Here, we reviewed research published over 35 years (between 1980 and 20192014) on illegal huntingactivities in PAs to understand identify the global and regional patterns of threats and to assess their impacts on species in protected areas. We evaluated what factors determine the likelihood that illegal activities lead to the decline in the populations of targeted animals. In particularSpecifically, we assessed whether the different legal status in different among PAs affects their ability to prevent population declines, and whether attributes of the species (i.e. body mass) and socio-economic context of a country (i.e. human development and agricultural land use change statuses) account for differences in PA success.(Cardillo *et al.*, 2005; Gaston & Blackburn, 1996) Finally, we draw on these results to propose recommendations for reducing impacts of illegal activities in protected areas.

Material and mMethods

Data collecation

Literature searches were carried out in We searched Web of Science, Science Direct, Google Scholar and Scopus between March 2014 and 2015 and in March 2020. Restricting the search period to 1980-2020, the search terms included: 'for all papers since between 1950 and 2020 using the search terms: 'illegal activity' OR 'illegal activities' OR 'illegal hunting' OR poach* *AND 'protected area' OR region name (e.g. Europe, Asia, North/South America, Africa and Australia, New Zealand) OR reserve OR 'biodiversity outcome'. Further search was performed

incorporating poach / wildlife poaching AND protected areas OR reserve OR region name (as above). All online searches for the publications were conducted between on 1215th November March 2014 2020and 10th March 2015. Full details of search terms can be found in the supplementary material. Results wereWe screened the returns based on criteria (i-iv below) that to ensureed the results from each paper were related to both PAs and illegal mammal huntingextraction of biodiversity:;

i. Whether the research was done in a protected area and addressed issues of illegal hunting of mammalsextraction of biodiversity; animals, plants or both.

ii.i. Whether the research was on illegal activities by a human population and a mention was clear that the extraction is from the named protected area.

iii.ii. If the research showed impact on species population within the protected areabeing extracted, decline or not declined

iv.iii. Only used primary data papers not meta-analyses or reviews.

v.iv. For results that returned more than one sStudyies covering similar the same protected area sites and year of data collection these were examined for relevance and only one that satisfied all criteria (i-iii) was included in the analysis.

We found no publications that fully satisfied the review criteria for the papers published between 1950 and 1980. Where two or more papers were published for the same protected area during a similar period of data collection, only one that satisfied all the criteria was used for the analysis. From each paper we extracted information on PA location, threat types, study species, perceived impacts of illegal resource, reason for illegal use huntingofftake, and purpose of research and data collection method. , continent (i.e. Africa, Asia, Europe, South America) and geographic region within continent (i.e. east, central, south and west). We recorded population trend (i.e. decline, no decline or unstated) for each PA/species combination from each paper as reported by

the paper and the reasons mentioned for such outcomesoutcome if not directly related to illegal offtake/hunting(e.g. illegal hunting, loggingtimber extraction) to examine their effect on population status in the PA. For example, if a paper investigated protected areas X and Y and reported impacts of illegal huntingactivities on various species i, j, k…,E then eEach species / site combination and associated variables became one row in the dataset, including the impact scores (1= species decline or 0 = no decline or NA = no reported impact for that species) and any covariate information for PA or paper (author, year of study, PA name, research method use, species name, species body mass, etc. see Supplementary material- SM-dataXX for the full database). The method arriving at the reported population trend status was recorded but not analyzed because most studies did not show the data used to arrive at a species outcome (e.g. decline), thus was difficult to tease apart whether the species decline was causal or correlative, a common problem in many meta-analysis studies (Lorties, 201418).

Species body size is likely to influence mammal hunting risk (Ripple et al. 2016). WFurther, we extracted body mass data is associated for each species e extracted body mass from . For mammals, we used the mammal database PanTHERIA (www.pantheria.org) and from EltonTraits (Wilman et al. 2014)the current IUCN species red book data (19). We used published literature to used The body mass of closely-related species for two species (i.e. *Eulemur rufifrons* and *Gazella dorcas massaesyla*) that were not located in these mammal databases were extracted from published literature(see supplementary material SM1). Body masses for reptile, amphibian and fish species were either extracted from the original papers (if provided) or from credible online material (supplementary material SM1). For bird body mass we used Dunning (20). To identify the geographical location of the study site, we cross-referenced the papers with Tthe WCMC IUCN Protected Planet database was used to identify the geographic location (latitude/longitude), and to assess whether and legal status (IUCN category) of each PA

influences the outcome of species populations, we extracted the IUCN category of each PA (21). to identify the coordinates of the centroid of each PA. We also searched from this PA database for each name of the researched protected area and recorded its appropriate category under the current IUCN PA categories.To explore the effect of legal status on species decline we grouped the PAs into two levels of protection: strict PAs (IUCN category I & II) and less strict PAs (categories III-IV). PAs in IUCN categories V-VI were excluded from analysis because they are not specifically designated for biodiversity conservation (Santini 2015).

To assess whether population change reported was related to broad wider scale economic or social change, -we extracted country-level human development (HDI) and agricultural land use change (ALC) indices from the UNDP and World Bank databases (22, 23). ALC is an index of measure of the amount of land converted to agriculture and other human activities such as settlement. ALC is associated with We used this index because recent studies report habitat loss within protected areas due to is driven by crop farming that is conducted inside or through encroaching on PAs protected area borders(de Lima et al. 2018, Maxwell et al. 2016, Geldmann et al. 2019) , and . Also, ALC is also was considered appropriate was chosen as it is related to illegal hunting threat because illegal farmers encroaching on PAs/loggers inside often engage in illegal hunting of animals (Bennett & Gumal, 2001) threat being invest. -We calculated the ALC over a decade period encompassing the times when research for the reviewed papers were conducted as most papers did not report the exact dates of data collection. We used HDI as a predictor rather than measure of socio-economic change and governmental effectiveness corruption rather that governance s of governance (the two are correlated) because HDI is a more direct indicator of development. Further, to understand the effect of different legal status on species decline we grouped the PAs into two levels of protection: strict PAs (for PAs under

IUCN category I & II) and less strict PAs (categories III-VI). Furthermore, we categorized species into two broad groups of mammal and non-mammal for analysis to examine any differences between groups in the way they are threatened by illegal activities. We placed reptile, bird, fish and mollusc into one group: "non-mammal" as data were too sparse for each of these taxa to be tested individually in the model. There was no reported status for any of the plant species in the dataset; therefore, we excluded records of plants from the analysis.

Data analysis

Several papers reported results for many species, and/or information for multiple protected areas. We Here weWe usedreferred to each unique combination of species within an individual PA (i.e. species × PA) reported in a paper as a study as an individual data point, with therefore potentially several studies per paper (see data extraction in methods and supplementary materials). For modelling, we excluded filtered all records where the population outcome was unknown. Using PA location and publication year, Wwe assessed analyzed spatial and temporal patternstrends in research effort of the previous work on of illegal hunting in PAsactivities and identified existing gaps. To assess whether mammal population declines are associated with generic factors such as PA level of protection, type of poaching, species and a country's socio-economic status, we used generalized linear mixed models (GLMM) with a binomial error term and logit link function using the statistical software R (version 3.2 R Development Core Team, 2015).

To examine the effect of species traits and socio-economic variables on illegal mammal hunting in PAs,these factors, first, Wwe we used generalized linear mixed models (GLMM) with a binomial error term and logit link function using the statistical software R (version 3.6.3). -For

all models, we excluded records where the population outcome was unknown, and the response variable was whether a species had declined in that PA (1) or not (0). We built an initial global model incorporating sixseven fixed factors: human development index (HDI), decadal percentage agricultural land use change index (ALC), log species body mass (grouped as small, medium and large), poaching type (commercial, subsistence, or combineda mixture), PA level of protection status (i.e. IUCN categories classified as strict and less strict) and, continent (Africa/Asia/outh America/Central and South AmericaEurope) and species group (i.e. mammal and non-mammal). Records from Europe were excluded from all analysis due to a low number of records (n=3). Because different species could relate to the same PA and country as studies from other papers at different times, we accounted for this by including country, paper and PA as random effects in all models and fitted the data datasets using the 'glmer' function implemented in the R-package 'lme4'(24). We used a backwards stepwise removal of non-significant terms (with Chi-test) to evaluate the relative effect of each factor on the population decline. We obtained model confidence intervals around variables showing statistical significance in the minimum adequate model using the Wald-method (24). Models of all analysis subsets were assessed using Akaike information criterion (AIC) to select the most parsimonious model after the stepwise removal of terms. We calculated a measure of variance explained by the models using the squared correlation between the response variable and the predicted values. These pseudo R2 values should be interpreted with caution as it only shows the variance explained by the fixed effects in the final model. comapre Furthermore, in each model we evaluated whether the likelihood of finding an impact on a species' population was due to the PA level of protection and level of illegal hunting (subsistence, commercial or both) and species body mass (small, medium and large)group and geographic regions. We also examined whether log species body size (small, medium, large), country's human development index and agricultural land use change index were consistently correlated with the population decline.

The majority of illegal mammal hunting records were from Africa and Asia. We therefore built two additional models using the same structure as the global model, but restricted data to Africa and Asia respectively. Finally, because the savanna elephant Loxodonta africana, had a large percentage of records (19%) in the Africa dataset, reflecting the increasing concerns for elephant poaching and illegal ivory trade (25), we built two further models for Africa: one excluding savanna elephants and another only including savanna elephants.

African elephants (i.e. mainly the African savanna elephant *Loxodonta africana*, but with some data for African forest elephant, *Loxodonta cyclotis*; no data on Indian elephants were included in the dataset), hereafter referred to as 'elephant', had large numbers of studies in the dataset (contributing XX22 % of all records), probably reflecting the increasing concerns for its poaching (25). To check the influence of this species abundance in the data, thus the generality of our results, we cross-examined the data by repeatinged the analysis using two subsets of the data: once for elephants alone, and once for all mammalanimal species except African elephants. Similarly, because there were sufficient data to analyze some components separately we repeated all the analyses with and without the strict PAs (IUCN categories I&II) as well as on separate subsets of data including Africa and Asia continents to examine their impact on the whole data set and the relative effects of different predictors between the two continents.

Results

Extent and scale of research on illegal hunting of mammals in protected areas

Our searches found 2245 papers in total, 819 of which satisfied our selection criteria for inclusion in the review. Nno publications that fully satisfied the review criteria for the papers

published between 1950 and 1980.……..The reviewed papers were from 48 countries and four continents, Africa, South America, Asia and Europe and covered 155 protected areas (Figure 1).

Formatted: Font: Not Bold

Formatted: Font: Not Bold

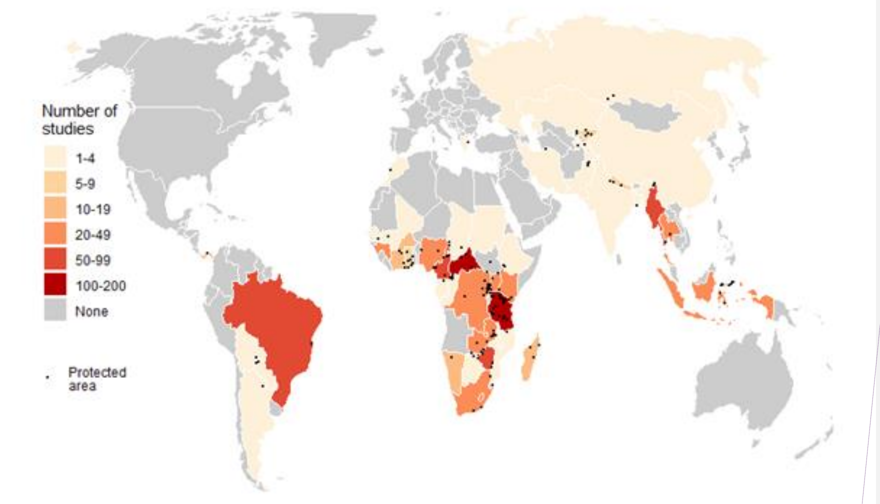

Figure 1. Spatial distribution of research on illegal hunting of mammals in 155 protected areas from 48 countries over four decades as collated in the literature. Black dots correspond to the centroid of a protected area where research for the reviewed papers was conducted.

There was an increasing trend in the research activities on illegal hunting in protected areas with the number of publications increasing two fold each decade since the 1980. Types of study methods have also increased, particularly studies that have included use of ranger patrol data and interview techniques These publications employed varying methods, from the use of patrol data, animal counts, market surveys to interviews or a mix of these methods (Figure 2). Two ninety eight thousand mammal species were reported to have been illegally hunted from the researched

Formatted: Font: Not Bold

protected areas during the four decades. animal counts, market survey to interviews or a mix of these (Figure 2).

Spatial distribution and trends in research on poaching in PAs

We identified XXX1598 papers that met the initial search criteria, of which 92 (Supplementary material SM2: reporting 1048 species x PA results = 'studies') met all the requirements and were used for analysis. The 8992 published papers researched 155146 protected areas from four continents, with the largest number of studies from Africa and Madagascar (XX819 studies), followed by Asia (XX162), South and Central America (XX66), and Europe (XX1) (Figure 1).

Formatted: Indent: Left: 0", First line: 0", Space After: 10 pt, Don't keep with next, Border: Top: (No border), Bottom: (No border), Left: (No border), Right: (No border), Between : (No border)

INSERT FIGURE ONE HERE]

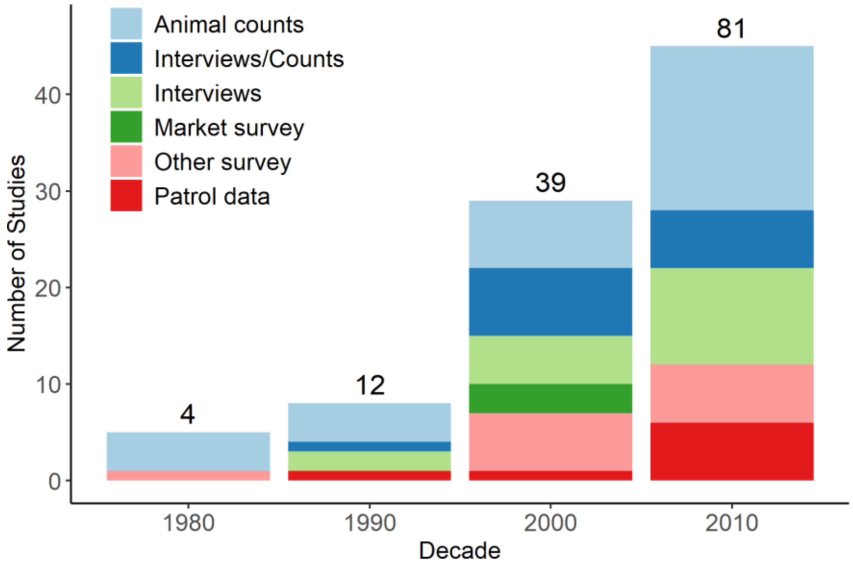

Figure 2. Number of studies per data collection method over 10-year periods. Numbers at top of bars indicate the cumulative number of publicationsNumber of studies per data collection method over 10-year periods. Numbers at top of bars indicate the cumulative number of publicationsat top of sbar for research in illegal hunting of mammals in 155 protected areas from 48 countries in Asia, Africa, South America and Europe.Figure 1. The locations of all PAs investigated (red dots correspond to centroids of protected areas). Numbers of studies (species x PA combinations) were: Africa and Madagascar (819), Asia (162), South and Central America (66), and Europe (1).

Most papers focused on single PA (i.e. local scale, n = 54 papers), or few PAs existing as one contiguous ecosystem and landscape (n = 39 papers). The majority (81%) of PAs studied were in the strictest IUCN categories (I-II). PAs All protected area types were investigated but the IUCN category II level of protection was researched the most. (57.35%, n = 65 papers; Figure 2). The There was no paper published between 1950 and 1979 that satisfied our search criteria: all relevant papers were published in 1980 onwards.

[INSERT FIGURE TWO HERE]

Figure 2. The number of studies (species x PA combinations) on illegal activities carried out in PAs with different levels of IUCN protection (I, greatest protection; VI, least). This is based on the 92 papers published between 1980 and 2014, encompassing four continents. There is a strong bias towards the PA category II. The IUCN PA categories are used to facilitate comparisons of

Formatted: Space Before: 6 pt, After: 6 pt, Border: Top: (No border), Bottom: (No border), Left: (No border), Right: (No border), Between : (No border)

different PA ‘entities’ in different countries (e.g. conservation area, forest reserve, game controlled area, game reserve, game sanctuary, marine reserve, national park and nature reserve).

Formatted: Space Before: 6 pt, After: 6 pt, Border: Top: (No border), Bottom: (No border), Left: (No border), Right: (No border), Between : (No border)

The research had varying purposes: investigating impacts (71 papers); conservation rationale (e.g. providing new methods for investigating illegal activities; 17 papers) and management/control of illegal activities (5 papers). Further, research has increased substantially during the last 35 years with a greaterwith greater number of published papers since 2005 (Figure S1); most of this increase was in Africa.

Furthermore, we found a temporal increase in the variety of research methods throughout the period (Figure 3). Interviews (n = 22 papers), animal counts (n = 28) and patrols (n = 9) were the most commonly used methods in the literature. Several research projects (n = 34) combined methods, while two used snare surveys. Animal count was the dominant research method during the first decade (1980-1990) and has increased in use since then. Use of interviewing and patrolling methods to investigate illegal activities in PAs has been used between 1990 and the present (i.e. 2014).

[INSERT FIGURE THREE HERE]

Figure 3. Methods used in the 92 publications considered. Recent studies show an increasing diversity of methodological approaches. ‘Other’ includes bone collection from poacher camps and sporadic field observations.

Patterns of species extraction and country socio-economic status

Three hundred and fifty-three species, comprising mammals (220), reptiles (18), birds (17), fishes (12), molluscs (2) and plants (84) were reportedly harvested illegally in the 146 protected areas (Table S1). These species were extracted for subsistence use (10.8%, N = 221), commercial use (19.9%) or both (60.6%). Almost nine percent of the studies did not report a reason of illegal resource extraction, and none of the plant studies included all the data that were required for this analysis. The countries where research was conducted showed varying levels of development (Figure 4) and land use change (Figure 5).

[INSERT FIGURE FOUR HERE]

Figure 4. Human development index (HDI) for the countries where research was conducted. HDI values shown are the mean scores for the total number of years that a country was researched.

[INSERT FIGURE FIVE HERE]

Figure 5. Agricultural land use (ALC) change index of a country where research was conducted. Negative change infers to loss of the natural habitats to agricultural activities and settlement. Mean ALC includes the total number of years a particular country PAs were researched during the last 35 years.

Socio-economic and species traits geographic factors influencing population decline in PAs threatened with illegal huntingactivities

In the global model, sSpecies body mass and PA strictness had a significant effect and species group (mammal or non-mammal) on the probabilityinfluenced probability of species decline in all PA. types across three continents (Asia, Africa, South America) .with The probability of a population decline in a protected area increased with body mass Sspecies with greater body mass especially mammals experiencing larger population decline (Figure 3, Table 1; model1) and populations in stricter PAs (IUCN categories I-II) had a lower probability of decline compared to less strict PAs (IUCN categories III-IV))(Figure 3, Table 1; model1)(Model 1 in Table 1, Figure 6a). When limiting analysis to examining Africa's only protected areas, we found that populations species were more likely to declined in countries with a lower HDI local human and for development and in species with greater body mass (Figure 4, Table 1; model 2). Accounting for the influence of studies on illegal elephant hunting, results limited to Africa and excluding Checking on the influence of over-represented species in our data (i.e. African elephants) showed that mammals with greater body mass were at greater risk of population decline in protected areas.and after removing it the results remained broadly similar, showing that population decline was still strongly in species with larger body mass (Table 1; model 3, FigureS11 without elephant). Restricting analysis to African elephants, showed that probability of population decline decreased with an increase in HDI (Table 1; model 4).

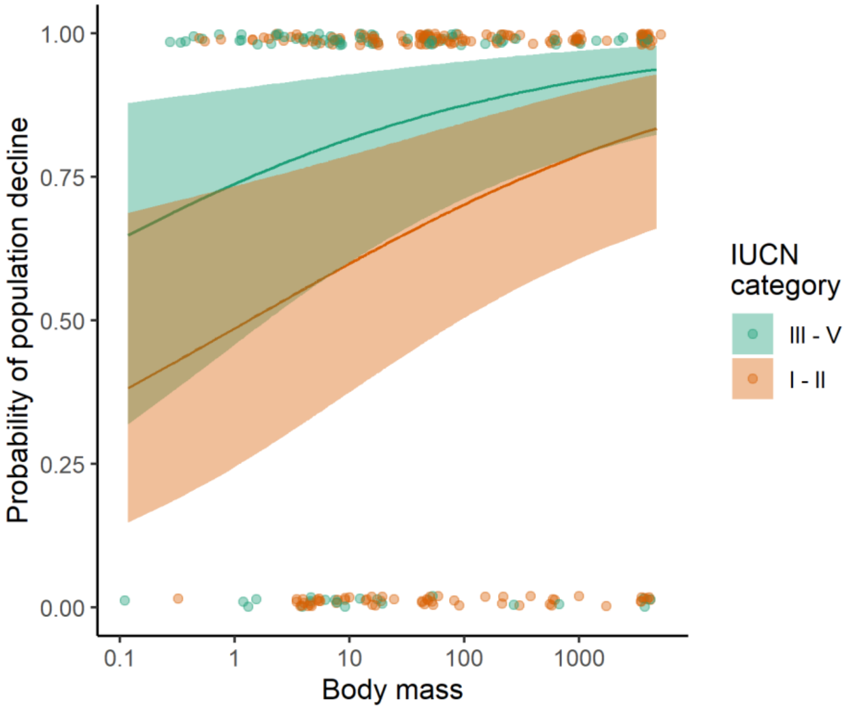

Figure 3. Effect of species body mass and protected area strictness onstrictnesstypes on the probability of decline of mammal species from global records.. Large bodied species had higher risk of decline when threatened with illegal hunting. There was no significant difference in the probability of population decline for mammals in different levels of protection. Shaded area shows 95% confidence intervals.CI around the estimates of effect size for the mammal and non-mammal species. Circles represent raw data points.

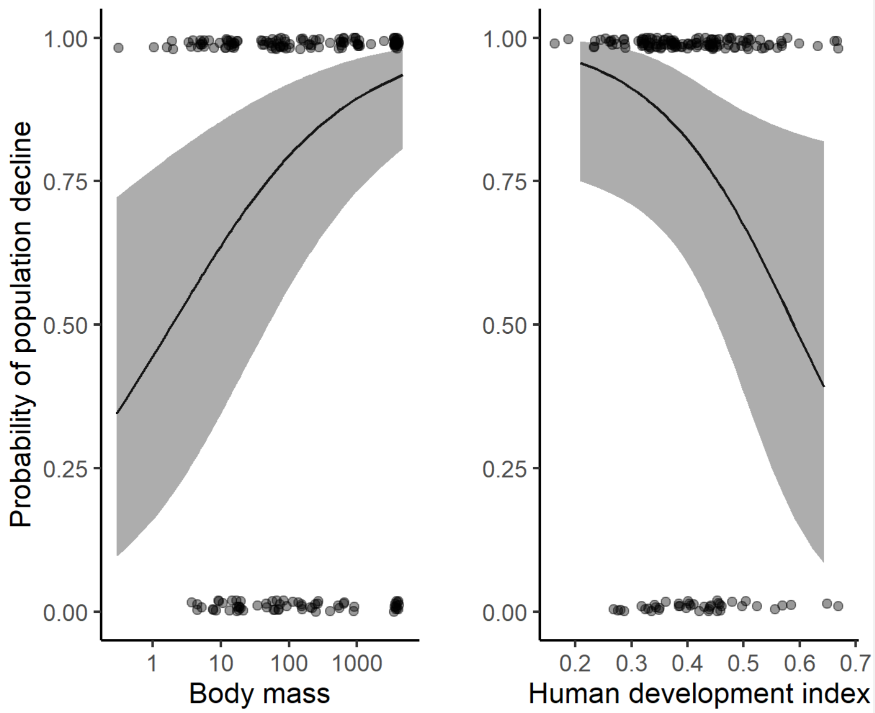

Figure 4. The probability of decline of mammals in Africa's protected areas threatened by poaching pressure showing population decline was strong in protected areas located in countries with low human development index and in species with larger body mass.

Results focussing on illegal mammals hunting in Asia showed that mammal populations in strictest PAs designated to protect biodiversity had a significantly greater probability of decline compared to mammals populations in less strict protected areas Interestingly, analysis restricted to Asia only revealed that the probability of decline was more strongly in mammals found in stricter (IUCN I-II) than less strict (IUCN III-VI) PAs (Figure 5, Table 1; model 5).

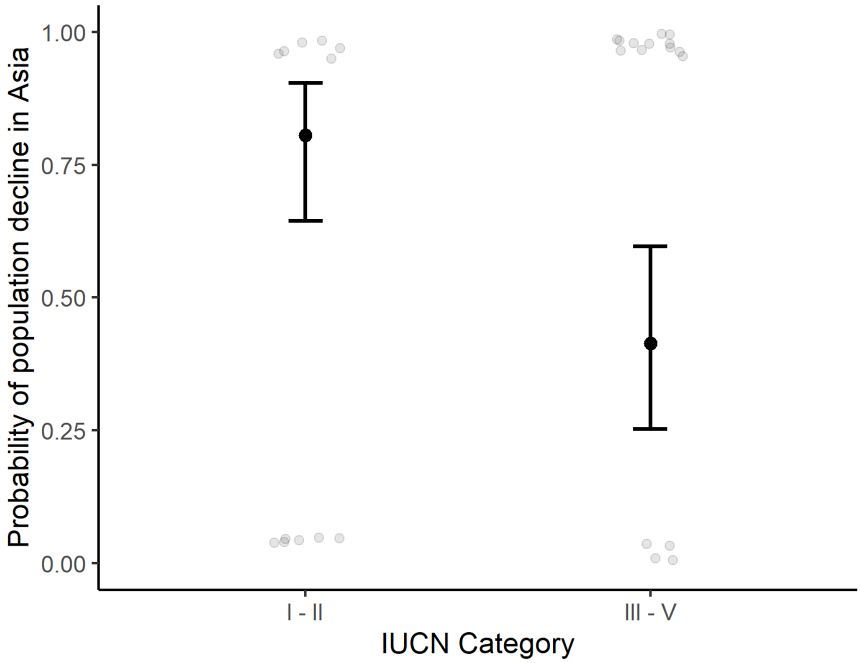

Figure 5. Probability of population decline for Asia's only protected areas indicating strong influence of protected area types. Higher decline of mammals was more likely in stricter than less-strict PAs.

Further, to separate the effect of potential legal hunting that could likely happen in less strict PAs to the validity of our results, we conducted a separate analysis involving stricter PAs (IUCN I-II)

only where hunting is strictly prohibited. The results did not differ with the general finding from all PA types combined: that any likely influence of potential legal hunting that could occur in the less strict PAs. Restricting the analysis to only strict protected areas (IUCN category I-II), the results showed that strict PAs in low human development index countries were associated with increased species decline, of mammal species with greater body mass.(FigureS2). by s There was an effect of the dominant species in our dataset. When removed the African elephants, we found that species declines were greater in PAs faced with commercial rather than subsistence poaching alone (Figure 6b); as before, mammals and species with greater body mass also exhibited the greatest declines in these models (Model 2 in Table 1).

Table 1. Results of the GLMM best models showing probability of species decline and the explanatory factors in PAs across four continents. Coefficient sign (+/-) indicates size of effect of covariables i.e. increase for plus and decrease for minus.

-	Type of model and effect size				-	-	-	-	-	-	-	-
-	Model1-all PAs/animal species			Model2-all species/PAs excl. elephants			Model3-strict PAs			Model4-less strict PAs		
Factor in final best model	Coefficients + SE	DF	p-value	Coefficients + SE	DF	p-value	Coefficients + SE	DF	p-value	Coefficients + SE	DF	p-value
Species body mass	0.225 ± 0.072	1	0.0013	0.243 ± 0.081	1	0.0018	0.379 ± 0.929	1	0.0001			
Human development index							-8.324 ± 3.526	1	0.0168			
Poaching level (subsist)				-0.221 ± 0.924	3	0.0474						
Poaching level (not given)				0.773 ± 1.496								
Poaching level (commercial)				1.879 ± 1.022								
Agric. land use change index										2.078 ± [illegible]	[illegible]	0.000 [illegible]
Species group (non-mammal)	-1.975 ± 0.910	1	0.0119	-1.792 ± 0.873	1	0.0188	-2.384± 1.231	1	[illegible]	[illegible]	[illegible]	[illegible]

Formatted: Space Before: 6 pt, After: 6 pt, Keep with next, Border: Top: (No border), Bottom: (No border), Left: (No border), Right: (No border), Between : (No border)

[INSER FIGURE THREESIX HERE]

Figure 36. Effect of species body mass (a) and level of poaching (b) on the probability of decline of mammal animal species (mammal-upper solid line, and non-mammal-lower dashed line) excluding the dominant species (African elephants) across all PAs (IUCN I-VI). Large bodied species had higher risk of decline when threatened with illegal hunting activities. There was no significant difference in the probability of population decline for mammals in different levels of protection. Shaded area shows 95% CI around the estimates of effect size for the mammal and non-mammal species.

Separate analysis on strict PAs according to protection level revealed variable results. Human development index (HDI), species body mass and species groups strongly influenced the probability that species would decline in strict PAs (Model 3 in Table 1, Figure 7). Strict PAs in low human development index countries were associated with increased species decline, of mammal species with greater body mass. In contrast, in less strict PAs (IUCN category III-VI), we found species decline was best explained by agricultural land use change (ALC) in the wider area; i.e. species in all PAs with greater habitat loss were more likely to have experienced population decline (Model 4 in Table 1).

[INSERT FIGURE SEVEN HERE]

Formatted: Left, Space Before: 0 pt, After: 0 pt, Border: Top: (No border), Bottom: (No border), Left: (No border), Right: (No border), Between : (No border)

Figure 67. HDI influence on population decline of animal species (upper solid line and lower dotted line for mammal and non-mammal respectively) within strict PAs (IUCN category I&II). Least developed countries had the highest probability of their PAs experiencing species decline. Shaded areas show 95% CI around the estimates of effect size of this factor.

The impact of illegal huntingactivities on African elephants was variable. Aacrosscross all PAs (IUCN I-VI), decline was highly associated with lower human development index high habitat loss; i.e. negative agricultural land use change index and with the geographic region of PA location (Model-All PAs in Table 2, Figure 8). This decline was greatest in central Africa, followed by the east and west; and least in southern Africa. In an analysis of strict PAs alone we found two factors (i.e. human development index and agricultural land use change index) associated with the increase in elephant decline (Model-strict PAs in Table 2). On the other hand, we found geographic region significantly associated with increased elephant decline within less strict PAs in central and east Africa (Model-less strict PAs in Table 2).

Table 1: Summary of GLMMsresults for the models testing to test the fixed effects of socio-economic factors and species traits on the probability of mammal decline in PAs. Values in brackets represent 95% confidence intervals.Models were tested for all data from three continents (Global model), a subset of data for Africa only, Africa without elephant data, Africa with only elephant data and Asia only. There were strong influences of the PA types, species body mass and human development index on the probability of mammal population decline within PAs.

Models	1	2	3	4	5

	Global	Africa only	Africa-ex.elephant	Africa- elephant only	Asia only
Human development index		-8.97*		-15.80**	
		(-16.59, -1.35)		(-26.33, -5.28)	
Body mass	0.21**	0.34***	0.37***		
	(0.07, 0.34)	(0.16, 0.53)	(0.17, 0.58)		
PA strictness	-1.11*				1.77**
	(-1.95, -0.27)				(0.66, 2.88)
Intercept	1.00	3.53	-0.08	8.25***	-0.35
	(-0.22, 2.21)	(-0.11, 7.17)	(-1.32, 1.16)	(3.36, 13.14)	(-1.09, 0.39)
Observations	523	374	302	72	65
Pseudo R2	0.37	0.47	0.43	0.68	0.16
Significance				*p<0.05; **p<0.01; ***p<0.001	

Table 2. Results from GLMM models with various factors explaining the probability of decline of African elephants across all PA types and separately between strict and less strict PAs.

-	Type of model and effect size				-	-	-	-	-
-	All PAs types (cat. I-VI)			Strict PAs (cat. I & II)			Less stric[illegible]		
Factor in final best model	Coefficients ± SE	DF	p-value	Coefficients ± SE	DF	p-value	Coefficients ± SE	DF	p-value
Human development index	-12.350 ± 4.207	1	0.0005	-19.459 ± 6.496	1	0.0004			
Agri. land use change index	-1.461 ± 0.699	1	0.0116	-1.173 ± 0.826	1	0.0371			
Africa zone (East)	0.723 ± 1.116	3	0.0100				0.406 ± 1.307	3	0.0282
Africa zone (South)	-2.186 ± 1.041						-2.773 ± 1.275		
Africa zone (West)	0.075 ± 2.749	-	-	-	-	-	0.981 ± 0.677		-

Formatted Table

[INSERT FIGURE EIGHT HERE]

Figure 8. The country's human development index (a) and agricultural land use change index (b) as best predictors of decline in the African elephant within in all PAs (category I-VI) across the African continent. High negative ALC values are associated with severe habitat loss and a high risk of population decline of African elephants. Gray area shows 95% CI around the estimates in the minimum adequate GLMM model.

Furthermore, the probability that illegal activities were associated with population declines also varied regionally across PAs in Africa, Asia and Latin America. Lower human development index of countries, greater species body mass and species group (i.e. mammal) were the strongest predictors of increased species decline in PAs across Africa (Model -Africa in Table 3). In contrast, species decline in Asia was strongly positively associated with PA strictness (Model-Asia in Table 3). On the combined data for Asia and Latin America, we found that increased probability of species decline in PAs across these continents was correlated with greater agricultural land use change (Model combined in Table 3).

Table 3. Results from GLMM models with influence of the various factors on the probability of species decline in PAs separately for Africa and Asia continents and on combined data for Asia and Latin America.

-	Type of model and effect size				-	-	-	-	-
-	Africa-all animals/PAs			Asia-all animals/PAs			Asia + L. America	-	-
Factor in final best model	Coefficients + SE	DF	p-value	Coefficients + SE	DF	p-value	Coefficients + SE	DF	p-value
Human development index	-8.657 ± 3.708	1	0.00386						
Body mass	0.338 ± 0.084	1	0.00022						
Species group (non-mammal)	-2.269 ± 1.095	1	0.01223						
PA strictness				1.974 ± 0.684	1	0.0237			
Agric. land use change index	-	-	-	-	-	-	-13.946 ± 4.741	1	0.0077

Discussion

Biodiversity in protected areas faces numerous anthropogenic threats (11). We analyzed data published since the 1980's to understand impacts of illegal huntingactivities on species population decline in protected areas. There was strong geographicstrongwere a strong protected area taxonomic and geographic biasbiases in research on illegal huntingactivities within protected areas dominated by records in Africa and Asiaduring the review period. Globally, we found that illegally hunted mammals in strict PAs (IUCN categories I-II) had a lower probability of population decline compared to less strict PAs (IUCN categories III-IV). In contrast, we found illegally hunted asian mammals in strict PAs had a greater probability of population decline than less strict PAs. Human development index had a strong influence on likelihood of mammal declines in Africa, while lspecies body mass

We found that population declines were more likely consequences of illegal activities huntinghuntnigofftake in countries with low human development index (HDI). Different groups of species declined at different rates, with arger bodied mammals were more mostly likely to show population decline globally and in Africa. As well as illegal huntingpoaching, we also found that the level of protection had little effect in protecting species against the negative impact of illegal hunting. Stricter PAs were mostly likely to experience mammal population decline than less strict PAs particularly in Asia. habitat loss had an additional impact on population decline of animal species in less strict PAs, particularly in countries experiencing greater agricultural land use changeFurther, species decline was also associated with the geographicwith geographic region of PA location being greater in Africa than Asia or Latin America.

Commented [2]: Create a test of different body size groups?

The identification of correlations between human development indices and illegal hunting in this study supports a widely held view, e.g. Bennett, Blencowe (26), Adams, Aveling (27), but one that is often based on limited data (28): that biodiversity decline is greater in relatively poor

regions. -Low human development scores could impact illegal huntingactivities in two ways: firstly, poor people may tend to exploit species illegally from PAs because they have limited alternatives (29). Secondly, poor countries have fewer resources to invest in PA conservation. Underfunding may result in increased illegal hunting, as well as -and other illegal activities such as agriculture.s crop farmingactivities in PAs due to insufficient law enforcement (30). This is supported by our model encompassing the most strictly protected areas alone, suggesting that more wealthy countries have which indicates high probability of species decline associated with low human development index countries (Table 1; model 1). Hilborn, Arcese (31) demonstrated that increased funding budgets for anti-poaching activities in the Serengeti National Ppark greatly reduced poaching pressures and ledlead to the recovery of the buffalo population. However, increasing conservation funding may not necessarily result ininto improved conservation particularly when social and political constraints exist. For example, social and political unrest may increase rates of illegal hunting and encroachment in protected areasactivities, reduce wildlife populations and thwart conservation efforts altogether (32, 33). Our results provide evidence that poverty, in as much as it is measured by the HDI, may have significant negative impacts on species due to accelerated illegal huntingpoaching, whether that be because of increased external pressures on PAs or decreased policing and protection within strict PAs. Further, at a global scale, illegal hunting activities increased species vulnerability to decline through increased habitat loss mostly in less strictly protected areas (Table 1, model 1). This could be because these PAs are often afforded minimal protection (34) and therefore this exposes them to intense illegal huntingactivities making them less reliable for the effective conservation of large and medium-size mammals (35). These findings also provide evidence that supports Craigie et al.'s (2010) and Ripple et al.'s (2019) assertions that illegal hunting and habitat loss are the major causes of continental-wide declines in megafauna in PAs across Africa

and other tropical regions (Craigie et al 2010, Ripple et al 2019). Our model that included only African elephants also highlights the importance of low human development index in relation to illegal ivory trade, and is consistent with recent analyses that this species is threatened with poaching and habitat loss across its habitat range in Africa (37, 38). Our study highlights the need to consider human development issues more seriously to ensure effective conservation of biodiversity within existing protected areas.

Large bodied species are likely highly susceptible to decline because they have slow growth rates and so overharvesting is likely to cause population decline (36). Low population growth rates in combination with multiple threats from illegal hunting ~~poaching~~ and diseases are known to have significant impact on population persistence (12, 15). By contrast, smaller mammals (with higher reproductive and growth rates) showed fewer declines and appeared to sustain harvest, though relatively few small species are the specific targets of poaching in ~~the~~ PAs. An alternative explanation to the large mammal decline observed in our data could be that large mammals, due to their relatively higher protein content compared to~~meat output over~~ smaller mammals they are mostly being targeted by illegal hunters for bushmeat sale~~particularly for meat sale~~. For example, a study in the Serengeti where illegal hunting is critical found that poachers prefer to hunt larger animals for their potential higher income returns (Ndibalema & Songorwa, 2008). ~~Our model that excluded elephants~~~~elephant suggested that commercial poaching has the greatest potential to cause population decline. Further, the propensity for elephants to decline in low human development index countries and geographic regions, and where habitat loss was taking place (Table 3), is consistent with recent analyses that this species is threatened with poaching and habitat loss across its habitat range in Africa (37, 38).~~

The pattern of species declines across the network of protected areas is worrying and suggests that PA policing (including access to appropriate conservation information) and resources need

to be improved. PA-specific information is important for understanding how illegal hunting~~activities~~ varies~~y~~ spatially and across time and there is a need to be able to predict future trends and thereby possible future management strategies e.g. Critchlow, Plumptre (39). Although at a global scale, mammals were less likely to decline by hunting in strict PAs, the opposite was true for Asian PAs ~~Furthermore, land use change poses additional risks of species decline in PAs across Asia and Latin America, relative to Africa, and its impact was more severe in less strict than strict PAs (Table S13).~~ This could be attributable to the habitat loss and poaching occurring inside these protected areas (40-41~~2~~), or the wider effects on animals that roam outside protected areas for parts of the year. These results are consistent with previous studies that have reported biodiversity decline and loss within PAs in these regions e.g. Geldmann, Barnes (16), Harrison (43), Laurance, Carolina Useche (44), Gallego-Zamorano et al. (2020). Our findings suggest that the effectiveness of Asian PAs~~land use change is a major threat that~~ requires urgent attention to improve biodiversity~~PA~~ conservation across in ~~Latin America and~~ continental Asia.

The geographical bias in the spatial distribution of research observed in these data is likely a consequence of interests among the researchers rather than being driven solely by the levels of illegal hunting~~activities~~ in particular PAs or countries. However, the temporal and spatial patterns of research observed in this study provide insight into the extent of the problem of illegal hunting~~activities~~ in PAs and therefore suggest that PAs are currently in need of new strategies to minimize impacts of illegal hunting pressure ~~activities~~ and to improve their conservation effectiveness (45). To date, research effort has concentrated on quantifying the extent and impact of illegal hunting~~activities~~ on focal species; in other words, documenting the problem. Far less information is available on which conservation management strategies (including human development and preventing illegal international trade, as well as within-PA

activities) are most effective at reducing illegal huntingactivitie pressures. Only XX5 out of 8892 publications reviewed here explicitly considered how the management of illegal huntingactivities might affect population declines. New research should focus on developing and testing new methods for reducing levels and impacts of illegal huntingactivities on wild mammal species in PAs.

Conclusion

Tackling illegal huntingactivities within protected areas remains a high conservation priority. Our results suggest that a combination of strategies may be required tohat simultaneously reduce the extent of illegalof frequency with which illegal hunting activities. W are activities isare attempted and reduce the likelihood that such attempts will be successful (from the perspective of the perpetrators). Regarding the former, we found that illegal huntingactivities in poor countries often leadslead to population declines within PAs, suggesting. These findings suggest that poverty alleviation may be an appropriate conservation strategy to reduce illegal huntingactivity pressures (26). The implication of this for local and national policies is that more effort needs to be invested to improve the social and economic status of the human populations. This needs to work in tandem with increasing the effectiveness of traditional conservation activities to prevent illegal huntingactivities; which may itself reduce the inclination of people to attempt future illegal hunting activities.. At the international level, these results may imply that PAs in low HDI countries may need more international support to curb pressures of illegal huntingactivities, particularly those driven by trans-boundary forces. Curbing external pressures to the PAs also needs improved efforts to prevent encroachment and other illegal activities such as crop farming within and squeezing of the PA borders. This will be urgent especially for Asia where stricter PAs are at greatest risk ofin dire consequences of losing their mammal populations. Sound strategies to stop elephant poaching and ivory trade, and trade in bushmeat,

as well as strengthening collaboration in conservation and research, are necessary to improve PA effectiveness in these countries.

Acknowledgements

We are grateful to the anonymous reviewers for the comments on earlier drafts of this manuscript. AAR wasis a commonwealth scholar funded by the U.K. government.

References

1. Chape S, Harrison J, Spalding M, Lysenko I. Measuring the extent and effectiveness of protected areas as an indicator for meeting global biodiversity targets. Philosophical Transactions of the Royal Society of London B: Biological Sciences. 2005;360(1454):443-55.

2. UNEP-WCMC. State of the World's Protected Areas 2007: An Annual Review Of Global Conservation Progress. UNEP-WCMC, Cambridge. 2008.

3. Zimmerer KS, Galt RE, Buck MV. Globalization and Multi-spatial Trends in the Coverage of Protected-Area Conservation (1980–2000). AMBIO: A Journal of the Human Environment. 2004;33(8):520-9.

4. Soutullo A, De Castro M, Urios V. Linking political and scientifically derived targets for global biodiversity conservation: implications for the expansion of the global network of protected areas. Diversity and Distributions. 2008;14(4):604-13.

5. CBD. Convention on Biological Diversity. Aichi Biodiversity Targets. Available at https://www.cbd.int/sp/default.shtml. Accessed May 2015. 2015.

6. Balmford A, Green RE, Jenkins M. Measuring the changing state of nature. Trends in Ecology & Evolution. 2003;18(7):326-30.

7. Craigie ID, Baillie JEM, Balmford A, Carbone C, Collen B, Green RE, et al. Large mammal population declines in Africa's protected areas. Biological Conservation. 2010;143(9):2221-8.

8. Mitchell D. Uganda Elephants near Extinction. Environmental Conservation. 1980;7(3):212-.

9. Wright SJ, Zeballos H, Domínguez I, Gallardo MM, Moreno MC, Ibáñez R. Poachers Alter Mammal Abundance, Seed Dispersal, and Seed Predation in a Neotropical Forest. Conservation Biology. 2000;14(1):227-39.

10. Leader-Williams N, Albon SD, Berry PSM. Illegal exploitation of black rhinoceros and elephant populations - patterns of decline, law-enforcement and patrol effort in Luangwa valley, Zambia. Journal of Applied Ecology. 1990;27(3):1055-87.

11. Butchart SHM, Walpole M, Collen B, van Strien A, Scharlemann JPW, Almond REA, et al. Global Biodiversity: Indicators of Recent Declines. Science. 2010;328(5982):1164-8.

12. Woodroffe R, Ginsberg JR. Edge Effects and the Extinction of Populations Inside Protected Areas. Science. 1998;280(5372):2126-8.

13. Milner-Gulland EJ, Bukreeva OM, Coulson T, Lushchekina AA, Kholodova MV, Bekenov AB, et al. Conservation: Reproductive collapse in saiga antelope harems. Nature. 2003;422(6928):135-.

14. Thirgood S, Mosser A, Tham S, Hopcraft G, Mwangomo E, Mlengeya T, et al. Can parks protect migratory ungulates? The case of the Serengeti wildebeest. Animal Conservation. 2004;7(2):113-20.

15. Cardillo M, Mace GM, Jones KE, Bielby J, Bininda-Emonds ORP, Sechrest W, et al. Multiple Causes of High Extinction Risk in Large Mammal Species. Science. 2005;309(5738):1239-41.

16. Geldmann J, Barnes M, Coad L, Craigie ID, Hockings M, Burgess ND. Effectiveness of terrestrial protected areas in reducing habitat loss and population declines. Biological Conservation. 2013;161(0):230-8.

17. Bruner AG, Gullison RE, Rice RE, da Fonseca GAB. Effectiveness of Parks in Protecting Tropical Biodiversity. Science. 2001;291(5501):125-8.

18. Taylor G, Scharlemann JPW, Rowcliffe M, Kümpel N, Harfoot MBJ, Fa JE, et al. Synthesising bushmeat research effort in West and Central Africa: A new regional database. Biological Conservation. 2015;181(0):199-205.

19. IUCN. The IUCN Red List of Threatened Species. Available at http://www.iucnredlist.org/. Accessed in April 2015. 2015.

20. Dunning JB. CRC handbook of avian body masses. Second ed: CRC press; 2007.

21. WCMCPP. WCMC IUCN Protected Planet Database. Available at http://www.protectedplanet.net/. Accessed between Feb-March 2015. 2015.

22. WB. The World Bank Datasets. Available at http://data.worldbank.org/indicator/AG.LND.AGRI.ZS. Accessed in March 2015. 2015.

23. UNDP. Human Development Reports 2014. Available at http://hdr.undp.org/en/content/human-development-index-hdi. Accessed in February 2015. 2015.

24. Bolker BM, Brooks ME, Clark CJ, Geange SW, Poulsen JR, Stevens MHH, et al. Generalized linear mixed models: a practical guide for ecology and evolution. Trends in Ecology & Evolution. 2009;24(3):127-35.

25. Wasser SK, Brown L, Mailand C, Mondol S, Clark W, Laurie C, et al. Genetic assignment of large seizures of elephant ivory reveals Africa's major poaching hotspots. Science. 2015;349(6243):84-7.

26. Bennett EL, Blencowe E, Brandon K, Brown D, Burn RW, Cowlishaw GUY, et al. Hunting for Consensus: Reconciling Bushmeat Harvest, Conservation, and Development Policy in West and Central Africa. Conservation Biology. 2007;21(3):884-7.

27. Adams WM, Aveling R, Brockington D, Dickson B, Elliott J, Hutton J, et al. Biodiversity Conservation and the Eradication of Poverty. Science. 2004;306(5699):1146-9.

28. Nellemann C, Henriksen R, Raxter P, Ash N, Mrema E. The Environmental Crime Crisis – Threats to Sustainable Development from Illegal Exploitation and Trade in Wildlife and Forest Resources. A UNEP Rapid Response Assessment. United Nations Environment Programme and GRID-Arendal, Nairobi and Arendal. 2014.

29. Brashares JS, Golden CD, Weinbaum KZ, Barrett CB, Okello GV. Economic and geographic drivers of wildlife consumption in rural Africa. Proceedings of the National Academy of Sciences. 2011;108(34):13931-6.

30. Keane A, Jones JPG, Edwards-Jones G, Milner-Gulland EJ. The sleeping policeman: understanding issues of enforcement and compliance in conservation. Animal Conservation. 2008;11(2):75-82.

31. Hilborn R, Arcese P, Borner M, Hando J, Hopcraft G, Loibooki M, et al. Effective enforcement in a conservation area. Science. 2006;314(5803):1266-.

32. de Merode E, Inogwabini B-I, Telo J, Panziama G. Status of elephant populations in Garamba National Park, Democratic Republic of Congo, late 2005. Pachyderm. 2007(42):52-7.

33. Bouche P, Mange RNM, Tankalet F, Zowoya F, Lejeune P, Vermeulen C. Game over! Wildlife collapse in northern Central African Republic. Environmental Monitoring and Assessment. 2012;184(11):7001-11.

34. Joppa LN, Pfaff A. Global protected area impacts. Proceedings of the Royal Society B: Biological Sciences. 2011;278(1712):1633-8.

35. Caro TM. Densities of mammals in partially protected areas: the Katavi ecosystem of western Tanzania. Journal of Applied Ecology. 1999;36(2):205-17.

36. Purvis A, Gittleman JL, Cowlishaw G, Mace GM. Predicting Extinction Risk in Declining Species. Proceedings: Biological Sciences. 2000;267(1456):1947-52.

37. Maisels F, Strindberg S, Blake S, Wittemyer G, Hart J, Williamson EA, et al. Devastating Decline of Forest Elephants in Central Africa. PLoS ONE. 2013;8(3):e59469.

38. Wittemyer G, Northrup JM, Blanc J, Douglas-Hamilton I, Omondi P, Burnham KP. Illegal killing for ivory drives global decline in African elephants. Proceedings of the National Academy of Sciences of the United States of America. 2014;111(36):13117-21.

39. Critchlow R, Plumptre AJ, Driciru M, Rwetsiba A, Stokes EJ, Tumwesigye C, et al. Spatiotemporal trends of illegal activities from ranger-collected data in a Ugandan national park. Conservation Biology. 2015;29(5):n/a-n/a.

40. Curran LM, Trigg SN, McDonald AK, Astiani D, Hardiono YM, Siregar P, et al. Lowland Forest Loss in Protected Areas of Indonesian Borneo. Science. 2004;303(5660):1000-3.

41. Corlett RT. The Impact of Hunting on the Mammalian Fauna of Tropical Asian Forests. Biotropica. 2007;39(3):292-303.

42. Leisher C, Touval J, Hess S, Boucher T, Reymondin L. Land and Forest Degradation inside Protected Areas in Latin America. Diversity. 2013;5(4):779.

43. Harrison RD. Emptying the Forest: Hunting and the Extirpation of Wildlife from Tropical Nature Reserves. BioScience. 2011;61(11):919-24.

44. Laurance WF, Carolina Useche D, Rendeiro J, Kalka M, Bradshaw CJA, Sloan SP, et al. Averting biodiversity collapse in tropical forest protected areas. Nature. 2012;489(7415):290-4.

45. Watson. JEM, Darling ES, Venter O, Maron M, Walston J, Possingham HP, et al. Bolder science needed now for protected areas. Conservation Biology. 2016;30(2):243-8.

SUPPORTING INFORMATION CAPTIONS

Table S1. The reasons recorded by the studies to explain the status of biodiversity reported in the reviewed papers. Plus (+) sign indicates the reason was mentioned together with the poaching in the PA. Studies (43.2%, n = 453) that sum up to the totals did not indicate the status of species being investigated and are not shown here.

~~Figure S1. Increasing publication trend for illegal activities in PAs during the last 35 years.~~

~~Table S2. 353 species extracted and threatened with illegal activities in 146 PAs across four continents as published in the last 35 years (1980-2014)~~

~~SM1: List of Additional Sources of data for Species body mass~~

~~SM2: Bibliography of the 92 research papers used in this analysis~~

1. Tittensor, D.P., et al., *A mid-term analysis of progress toward international biodiversity targets.* Science, 2014. **346**(6206): p. 241-244.
2. Geldmann, J., et al., *A global-level assessment of the effectiveness of protected areas at resisting anthropogenic pressures.* Proceedings of the National Academy of Sciences, 2019. **116**(46): p. 23209-23215.
3. Visconti, P., et al., *Protected area targets post-2020.* Science, 2019. **364**(6437): p. 239-241.
4. CBD, *Convention on Biological Diversity. Aichi Biodiversity Targets. Available at https://www.cbd.int/sp/default.shtml. Accessed May 2015.* 2015.
5. Waldron, A., et al., *Targeting global conservation funding to limit immediate biodiversity declines.* Proceedings of the National Academy of Sciences, 2013. **110**(29): p. 12144-12148.
6. Schleicher, J., et al., *Conservation performance of different conservation governance regimes in the Peruvian Amazon.* Scientific reports, 2017. **7**(1): p. 1-10.
7. Venter, O., et al., *Bias in protected‐area location and its effects on long‐term aspirations of biodiversity conventions.* Conservation Biology, 2018. **32**(1): p. 127-134.

8. Geldmann, J., et al., *Effectiveness of terrestrial protected areas in reducing habitat loss and population declines.* Biological Conservation, 2013. **161**: p. 230-238.
9. Benítez-López, A., et al., *The impact of hunting on tropical mammal and bird populations.* Science, 2017. **356**(6334): p. 180-183.
10. Jones, K.R., et al., *One-third of global protected land is under intense human pressure.* Science, 2018. **360**(6390): p. 788-791.
11. Benitez-Lopez, A., et al., *Intact but empty forests? Patterns of hunting-induced mammal defaunation in the tropics.* PLoS biology, 2019. **17**(5).
12. de Lima, D.O., M.L. Lorini, and M.V. Vieira, *Conservation of grasslands and savannas: A meta-analysis on mammalian responses to anthropogenic disturbance.* Journal for Nature Conservation, 2018. **45**: p. 72-78.
13. Brodie, J.F., et al., *Correlation and persistence of hunting and logging impacts on tropical rainforest mammals.* Conservation Biology, 2015. **29**(1): p. 110-121.
14. Ripple, W.J., et al., *Bushmeat hunting and extinction risk to the world's mammals.* Royal Society open science, 2016. **3**(10): p. 160498.
15. Allan, J.R., et al., *Hotspots of human impact on threatened terrestrial vertebrates.* PLoS Biol, 2019. **17**(3): p. e3000158.
16. Maxwell, S.L., et al., *Biodiversity: The ravages of guns, nets and bulldozers.* Nature News, 2016. **536**(7615): p. 143.
17. Gallego- Zamorano, J., et al., *Combined effects of land use and hunting on distributions of tropical mammals.* Conservation Biology, 2020.
18. Ripple, W.J., et al., *Bushmeat hunting and extinction risk to the world's mammals.* Royal Society Open Science, 2016. **3**(10).
19. Edwards, D.P., et al., *Conservation of tropical forests in the anthropocene.* Current Biology, 2019. **29**(19): p. R1008-R1020.
20. van Velden, J., K. Wilson, and D. Biggs, *The evidence for the bushmeat crisis in African savannas: a systematic quantitative literature review.* Biological Conservation, 2018. **221**: p. 345-356.
21. Ripple, W.J., et al., *Are we eating the world's megafauna to extinction?* Conservation Letters, 2019. **12**(3): p. e12627.
22. Watson, J.E.M., et al., *The performance and potential of protected areas.* Nature, 2014. **515**(7525): p. 67-73.
23. Lindsey, P.A., et al., *The bushmeat trade in African savannas: Impacts, drivers, and possible solutions.* Biological Conservation, 2013. **160**: p. 80-96.
24. Ndibalema, V.G. and A.N. Songorwa, *Illegal meat hunting in serengeti: dynamics in consumption and preferences.* African Journal of Ecology, 2008. **46**(3): p. 311-319.

Response to reviewers' comments

Spatial distribution and research trend of illegal activities and the factors associated with wild mammal population declines in protected areas

Response to Editor

'Both the reviewers have appreciated your idea and found it intriguing and worth publishing the potential results of this research. There are several aspects, however, that must be carefully addressed before I can consider your paper for publication.

In general, the current draft of the manuscript is confusing, and it is not presented in an attractive way so. The manuscript has to be fully reviewed by a native English speaker. The vocabulary needs a revision (see reviewer #2) to be consistent with the literature and through the text. Both reviewers point out the need to complete the review of literature, including the scientific papers published in the last years (from 2016 to date).

I agree with the concerns of the reviewer #2 on the statistical analysis. Please, pay attention to provide a full reply to the comments about this issue. Moreover, I did not understand your description of the results about figures 6 and 7: I think that these trends are not significant, because the CIs are very large and they include the variations described by the regression lines.'

Response: Our literature review has been updated to include studies upto 2020. We have rewritten much of the manuscript and clarified and added more details to our methods section. The title has changed to reflect the focus on mammals in protected areas. All models are new and be have focussed our modelling to 5 questions focussing on 1) global mammal declines in protected areas, 2) mammal declines in Africa, 3) mammal declines in Africa excluding elephants, 4) mammal declines in Africa - elephants only and 5) mammal declines in Asia. There are two 'elephant' models included because much of Africa data consists of elephant poaching studies. All the results we have presented are new and more focussed to out aims which we have explicitly set out in the introduction.

Response to reviewer 1

<u>Abstract</u>
Line 34 - It should be 1980-2014?
Response: This has now been corrected and our analysis now includes data upto 2020 (line 33)

<u>Introduction</u>
Line 75 - change "hunting" to "hunting/poaching"
Response: We now use the term 'illegal hunting' throughout the manuscript instead of hunting, poaching or illegal activities.

Line 76 - Insert "threat" in the "each threat can reduce..."
Response: We have now rewritten the introduction, this original line has been removed.

Line 95 - conjugate verbs in the past tense. E.g. change "review" to "reviewed".
Response: We have now used past tense throughout the manuscript

Line 96 - 1980-2014?
Response: This has now been corrected to include data up to 2020

Line 96 - Why only until 2014? A literature review paper should be updated. It has been 6 years and certainly many studies have been published across the globe and should be included in the analysis before the MS is considered for publication
Response: Published studies up to 2020 have now been included in the analysis

Line 98 - change "evaluate" to "evaluated".
Response: We have now rewritten the introduction, this original line has been removed. Past tense is now used where necessary

Line 99 - We assessed
Response: We have now rewritten the introduction, this original line has been removed. Past tense is now used where necessary

Line 106 - "Material and methods"
Response: This has now been corrected, changed to 'Material and methods (line 119)

Line 107 - Data collection?
Response: This has now been corrected, changed to 'data collection' (line 120)

Overall, I recommend that MS be reviewed by an English native prior to publication.
Response: Thanks, the lead author is not a native English speaker, but the current MS has now been reviewed and commented on by native English speakers to correct tense, grammar and spelling.

Line 109 - What is the reason for being from 1950? Authors must define why this temporal cut. Also, in the abstract and introduction is written from 1980-2014?
Response: Search was intended to capture all studies since 1950 to date, however no relevant paper was retrieved until 1980. 1950 was chosen as an arbitrary year, but we have now updated the MS to state why this is a review from 1980-2020.

Lines 112-113 - Again, a review study of the scientific literature must be current. Therefore, it is important that the authors include data by 2019 at least.
Response: Published studies up to 2020 have now been included in the analysis

Lines 147-150 - The MS title is said to be a study of mammalian populations. It may be better to modify for vertebrates since the authors have used other taxonomic groups.
Response: The current MS currently focuses on mammals

Lines 165-167 - Again, if other taxonomic groups were analyzed, it is important to modify the title of the study.
Response: As above, we have clarified that the study now only focuses on mammals

Line 173 - "referred"
Response: We have now rewritten the introduction, this original line has been removed. Past tense is now used where necessary

Lines 227-228 - Importantly, the period included in the search was 1950-2014 and not 1980-2014. The fact that no studies were found until 1979 is a result.

Response: This has been corrected and search period is reported appropriately (Line 122)

Lines 288-289 - ??? **Re-written and Corrected**
Line 293 - ??? **Re-written and Corrected**
Line 333 - ??? **Re-written and Corrected**
Line 339 - ??? **Re-written and Corrected**
Line 360- ??? **Re-written and Corrected**
Line 364 - ??? **Re-written and Corrected**
Response:The results section has been fully rewritten as a result of the peer review. We have now focussed our analysis on mammals, and have fewer GLMM models focussing on 1) global data, 2) Africa and 3) Asia

Line 430 - ???
Response: (Re-written and Corrected)

The MS presents important information to understand how impacts on protected areas are distributed across the globe. And no doubt it is of great relevance to conservation. However, it is necessary to update the data before publication! A literature review study should be as current as possible at the time of publication. That is, authors must at least include data from papers by 2019.

Response: Thanks. We have rewritten much of the MS, updated our literature search and compiled new models to focus our analysis on mammals.

Response to reviewer 2

The researchers of this paper made a review of the illegal activities affecting populations of different species inside protected areas (PAs). This is a very relevant topic because it is increasingly shown that protected areas are not enough to stop the biodiversity crisis we observe worldwide. Especially, in the last years, it has been shown that overexploitation and land-use change are the main drivers of biodiversity loss. However, the paper does not include any reference to recent studies (the most recent reference is from 2016) and therefore I think they missed some relevant and new insights on the topic. I believe that the manuscript and their database has potential for publication, yet, the manuscript was confusing in many parts, difficult to follow, and not presented in an attractive way so, in my opinion, it requires a profound revision from the authors to clarify several aspects and improve the scientific basis of it.

In general, I think that more attention needs to be paid to the vocabulary and the overall aim of the paper. In the introduction (and title) they talk about illegal activities, however, in the methods they explain that they only included illegal extraction and no other illegal activity as such for the analysis (criteria ii). I would frame the paper differently because otherwise, the reader expects a different outcome from it. Moreover, the methods were really short and some decisions that they made were difficult to understand, e.g., why put all non-mammals together? The dataset although relatively large is too patchily distributed and it should be refined (filtered) before being analysed. I suggest to restrict the analysis to Africa as most of the data (~80%) is from there. In contrast to the methods, the results were quite extensive with some non-informative figures that could be merged or eliminated, tables that could be clearer, and paragraphs that also need clarification (see comments). Overall, the discussion was easier to follow than other parts of the paper, however, as I had difficulties following the general setup and I think that some of the results cannot be fully trusted due to low data points, most of my specific comments regard the methods.

Response: Many thanks for the reference suggestions. We have incorporated the latest literature and rewritten and much of the manuscript to focus on mammal and PA effectiveness. We have clarified our aims and questions (lines 111-116) and ensured that the data analysis uses the most suitable database subsets; 1) a global, 2) three models focussing on Africa and 3) a model focussing on Asia. The Database used is supplied.

Title

The main title is misleading as it talks about illegal activities, while the paper is about poaching only. Therefore, the short title is much better as it focusses on the poaching impact. Please, consider changing the title

Response: This has now been changed to reflect the updated analysis and focus of the manuscript on mammals. It now reads "Global extent and drivers of mammal population declines in protected areas under illegal hunting pressure"

Introduction

Lines 66-71: Please revise recent literature on large-scale patterns on the efficiency of PAs (e.g. Geldmann et al. 2015, 2019) and wilderness areas (Di Marco et al. 2019) to protect biodiversity. Also check the effect of hunting and land use on biodiversity worldwide (Brodie et al. 2015; Ripple et al. 2016; Benítez-López et al. 2017, 2019; Gallego- Zamorano et al. 2020) because in your citations, you only focus on local assessments but there has been an effort on the last years to assess overall impacts of these pressures. This also applies to other parts of the text e.g. Lines 91-93 and discussion.

Response: Many thanks for these suggestions. Our introduction (and discussion) have been fully rewritten to include up-to-date literature and more relevant citations. We believe our manuscript stands apart from recent similar analysis (e.g. Benítez-López et al. 2017, 2019) because our interest is in the population impact within PAs and therefore a broad scale assessment of PA effectiveness using specific PA population trend data.

Line 96: Both here and in the abstract the years (I supposed) are wrong, please correct.
Response: This has now been corrected and literature search updated to include all articles up to end of 2019 and early 2020 (line 33)

Methods

Lines 115: Until now, you talked only about illegal activities in general and mentioned illegal extraction (poaching) as one of them. Here, you restricted your search to only studies that included illegal extraction (criteria i and ii), which is fine but mislead the overall aim of the paper.
Response: The current manuscript focuses on illegal hunting. Our database is also restricted to mammals.

Line 127-129: What if several studies satisfied all the criteria, how did you select the paper?
Response: It was not possible for a paper to satisfy all criteria. For example a paper can be both a review and primary data paper. We have clarified the criteria used in the data selection (lines 127-133)

Line 134: But logging was not included in the analysis right?
Response: Correct, our updated analysis focuses on mammals for which the data are most numerous. Reference to 'logging' has been removed

Line 139-140: I would like to know all the information that you put in the database and see the full database that you used for the analysis. Please, remove the "etc" and name all the variables, do not be short in words.
Response: We have now clarified and specified the data collated in the database (lines 140). A full dataset with all the variables included in this analysis is attached as supplementary material.

Lines 140-143: Do you use meta-analytical tools for the analysis or not? If yes, which type of weighting method did you use for each study (sample size, variance)? How can I believe that your analyses are robust? Reference 18 is not suitable for this statement.
Response: We assessed performance of all model analysis using Akaike Information Criterion (AIC) and selected the most parsimonious model after the stepwise removal of terms (Lines 189-191). Ref 18 replaced by a more appropriate citation (Line 144).

Line 149: The fish data is missing in SM1, is all the body mass coming from primary sources?
Response: All non-mammal taxa have now been excluded from the current analyses.

Line 155-162: As you focus on population changes of species inside PAs, could you use spatial analysis to calculate for example the agricultural land-use change inside each of the PAs? I propose this because PAs, in general, are not big and species suffer more from local-scale changes (e.g. deforestation or hunting in the area) than from country-level changes. Some areas of a country can be heavily degraded while others are intact, therefore a more detailed and spatially-explicit analysis will benefit the paper in my opinion.
Response: We think using a country-level agricultural land use change data was appropriate given that HDI was also a country-level index. Also evidence from literature suggests increased pressures associated with land use demand in the wider matrix often squeezes protected areas border and degrate inside PAs through agriculture (Lines 160-162).

Lines 164-165: If the purpose of your study is to assess the effectiveness of PAs against illegal activities, specifically illegal extraction, please consider to re-do the levels of PAs and stick to PAs that are designated at conserving biodiversity. Categories I- IV, are designated for that, with I-II being stricter. Categories V- VI allow sustainable use (see Santini et al. 2016; Oldekop et al. 2016).
Response: Our search criteria filtered PAs that allow legal hunting and in our initial analysis we conducted separate analyses involving both stricter and less strict PAs to decouple the potential confounding effect of potential legal hunting in less strict PAs. We agree that the analysis should be restricted to protected areas designated at biodiversity conservation. Therefore, our new analysis has removed all data points in IUCN categories V and VI. The spatial distribution (figure 1) and temporal trends (figure 2) do still include records from all data points.

Lines 167-170: This can mislead the results because reptiles, birds, fishes and mollusc have very different ecological requirements and their extraction is very different. If the data was poor, then I would simply not use it. Same applies to the spatial data, I would not include the European study nor (maybe) the American ones.
Response: We have now restricted the data analysis to mammals only and removed the European data. As this is a global analysis, we have still included the Central and South America data (many more data points from the updated literature search) in the global model, but we have also focussed on the area with most data in separate analysis, specifically Africa and Asia.

Lines 171-209: In this paragraph, I would specify more explicitly that the response variable of your models is species decline (1) or not (0). This is mentioned in the previous section but as an example, so here I was a bit confused about which response variable you used.
Response: We have now made it explicit in the text that the response variable in all models is species decline in a PA (1) or not (0). (line 178)

Line 183: Please be consistent with the vocabulary. Here, you use poaching type, but in line 194 you use hunting level (same applies to the whole paper). Poaching refers to illegal hunting, but the level of hunting is something different, e.g., intense vs not intense hunting. I would not use poaching and hunting interchangeably as hunting can be legal.
Response: We now use ’illegal hunting’ throughout the text

Line 188: Which datasets? Do you have more than one?
Response: This has been corrected. Our term ‘datasets’ represent different subsets of the illegal hunting database used to answer or specific questions. (line 195)

Lines 195-197: How did you do this? Please, expand the explanation.
Response: We have now re-written these lines to clarify that all models included these variables prior to model selection. (lines 178-9)

Line 202-204: How about using cross-validation instead of a subset data specifically for elephants? What is sufficient data in this case?
Response: This has been corrected. Cross validation of GLMMs would not be appropriate in this analysis. We therefore use separate GLMMs on subsets of data to answer our specific questions which now focus on 1) global data, 2) Africa (with and without elephant data) and 3) Asia. We have excluded analysis focussing in PA strictness as it's not appropriate, but sufficient data would be models that include at least 5 blocks of random effects (in this case our random effects were study, country and protected) (lines 178-200)

Line 204-206: So if I understood correctly you have three sets of data, in all sets, you started from a global model and did backwards removal to assess the generality of the effects, is this correct? Did you use any model selection criteria (AIC or BIC) to assess if the model with all the significant variables was the best or not? Moreover, as I said, I would stick only to the continents with enough data (Africa and Asia) the rest are quite scarce (maybe even only Africa as it accounts for 80% of your data). Figure 1 shows how this is a problem with very few points in Europe and America.
Response:We have now clarified our model selection process, which did originally include AIC model selection criteria. As noted earlier, we have focussed our data analysis to areas where most of the data are 1) globally, 2) africa and 3) asia. FIgure 1 still includes all illegal mammal hunting records in the database, but some data (e.g. from Europe) were not used for analysis.

<u>Results</u>
In my opinion, there are too many figures (8 in total) and some of them are not very informative, please, consider to remove some of them or combine them. For instance, Figure 2 could be replaced with text.
Response: Agreed. We have limited our descriptive results to spatial and temporal trends. Our original questions have been refined so that we now use fewer models in the analysis.

Lines 246-252: These two paragraphs could be merged in one, Figure 3 already shows the increase in studies so no need for Figure S1. Moreover, is there a reason for the different colors in Figure 3?
Response: We have replaced all figures in the main text and supplementary data to highlight our more focussed analysis.

Line 263-264: These numbers also show a strong biased towards mammals with very low numbers for the rest of the groups, therefore, I would suggest to completely remove them from the analysis and not include them as “non-mammals”.
Response: Agreed, our new analysis now focuses on mammals throughout the manuscript

Line 286-292: This paragraph and Table 1 are really confusing. You mix all different models and is really difficult to follow. Why Figure 6b is the model without African elephants? And did you only removed African elephants or all elephants as mentioned in line 200? Again, please be consistent with terminology. Moreover, I don’t understand Model 1, how is it possible that none of the poaching types (not levels) is significant when including the elephants? Aren’t elephants very influenced by poaching (line 202)?
Response: Our analysis has now been refined to 5 GLMMs. We still include a model with and without savanna elephants (*Loxodonta africana*) but have clarified why these subsets of the database have been used, i.e., due to a high number of records due to to the research interest in elephants and ivory (line 178-200)

Line 302: I thought Figure 6a is for Model 1 (line 288-289) but in the caption, you put that is for the model without elephants, can you clarify it?
Line 315: I think you confuse the terminology here is not best explained but significantly affected by agricultural change. Could you provide the explained variance by each of the models, i.e. marginal and condition R2?
Response: It is not possible to calculate traditional R^2 values for GLMMs due to the influence of the random effects. We have however computed pseudo R^2 for each model (Table 1) based on the squared correlation between the response variable and the predicted values. These pseudo R^2 should be interpreted with caution as it only shows the variance explained by the fixed effects in the final model. We have stated this in the text also (lines 191-194)

Line 356: Did you actually removed Europe? How about Central America? Why did you combine Latin America+Asia?
Response: As noted above, we have now refined the data analysis to exclude Europe and focus on 1) global, 2) africa and 3) Asia.

Line 360: So the stricter the more decline?
Response: We have refined the analysis to focus on particular subsets of the database, but have still included a model on data in Asia. We have clarified this result, and yes, mammal populations in stricter PAs (IUCN categories I-II) had a greater probability of decline compared to less strict PAs (IUCN categories III-IV). (lines 252-254)

<u>Discussion</u>
Please, do not talk about illegal activities in general through all the discussion as you only included illegal extraction (poaching) in the analysis, focus on that.
Response: Yes, as with the rest of the improved MS, we have focussed the discussion on illegal hunting rather than refer to illegal activities.

Lines 376-377: Could you maybe create different body size groups to test this?
Response: Because we were not clear in the original MS about the analysis, we feel this is not necessary. Body mass was included in all updated models (except model 4, elephant only) as a continuous variable. Splitting the data into groups does produce the same general result i.e, population decline as a result of illegal hunting increase with body size.

Line 378: You did not calculate habitat loss as such, even less at the PA scale. Is there any animal in your list that have affinity for agricultural areas? If yes, then the increase in agriculture for them is not habitat loss.
Response: Agriculture activities around borders and inside PAs degrade habitats which then affect mammals. Yes, we agree that the effect of habitat degradation, thus loss, may be variable among species due to species-specific responses but still its impact is often considerable for most species. Thus we still believe that a country-level agricultural land use change will have an impact on the preserved areas which are potentially suitable for agriculture. We have provided text to clarify this (line 159-160)

Suggested references
Response: We have considered these literature in the re-written introduction of the MS.

Benítez-López A, Alkemade R, Schipper AM, Ingram DJ, Verweij PA, Eikelboom JAJ, Huijbregts MAJ. 2017. The impact of hunting on tropical mammal and bird populations. Science 356:180–183. Available from: http://science.sciencemag.org/content/sci/356/6334/180.full.pdf.
Benítez-López A, Santini L, Schipper AM, Busana M, Huijbregts MAJ. 2019. Intact but empty forests? Patterns of hunting-induced mammal defaunation in the tropics. PLOS Biology 17:e3000247. Available from: https://doi.org/10.1371/journal.pbio.3000247.
Brodie JF, Giordano AJ, Zipkin EF, Bernard H, Mohd-Azlan J, Ambu L. 2015. Correlation and persistence of hunting and logging impacts on tropical rainforest mammals. Conservation Biology 29:110–121. John Wiley & Sons, Ltd. Available from: https://doi.org/10.1111/cobi.12389.
Di Marco M, Ferrier S, Harwood TD, Hoskins AJ, Watson JEM. 2019. Wilderness areas halve the extinction risk of terrestrial biodiversity. Nature 573:582–585. Available from: https://doi.org/10.1038/s41586-019-1567-7.
Gallego- Zamorano J, Benítez- López A, Santini L, Hilbers JP, Huijbregts MAJ, Schipper AM. 2020. Combined effects of land use and hunting on distributions of tropical mammals. Conservation Biology:cobi.13459. Available from: https://onlinelibrary.wiley.com/doi/abs/10.1111/cobi.13459.
Geldmann J et al. 2015. Changes in protected area management effectiveness over time: A global analysis. Biological Conservation 191:692–699. Available from: http://www.sciencedirect.com/science/article/pii/S0006320715300793.
Geldmann J, Manica A, Burgess ND, Coad L, Balmford A. 2019. A global-level assessment of the effectiveness of protected areas at resisting anthropogenic pressures. Proceedings of the National Academy of Sciences:201908221. Available from: http://www.pnas.org/content/early/2019/10/22/1908221116.abstract.
Oldekop JA, Holmes G, Harris WE, Evans KL. 2016. A global assessment of the social and conservation outcomes of protected areas. Conservation Biology 30:133–141. John Wiley & Sons, Ltd. Available from: https://doi.org/10.1111/cobi.12568.
Ripple WJ et al. 2016. Bushmeat hunting and extinction risk to the world's mammals. Royal Society open science 3:160498. The Royal Society. Available from: https://www.ncbi.nlm.nih.gov/pubmed/27853564.
Santini L, Saura S, Rondinini C. 2016. Connectivity of the global network of protected areas. Diversity and Distributions 22:199–211. Available from: http://doi.wiley.com/10.1111/ddi.12390.
