# Supplementary material for: Global extent and drivers of mammal population declines in protected areas under illegal hunting pressure
Source: PLoS One. 2020 Aug 21;15(8):e0227163. doi: 10.1371/journal.pone.0227163 (PMC7446782; doi:10.1371/journal.pone.0227163)
Supplement: Attachment — Submitted filename: Response to reviewers - Wild mammal declines in PAs.docx [file pone.0227163.s003.docx]

**Response to reviewers’ comments**

**Spatial distribution and research trend of illegal activities and the factors associated with wild mammal population declines in protected areas**

## Response to Editor

‘Both the reviewers have appreciated your idea and found it intriguing and worth publishing the potential results of this research. There are several aspects, however, that must be carefully addressed before I can consider your paper for publication.

In general, the current draft of the manuscript is confusing, and it is not presented in an attractive way so. The manuscript has to be fully reviewed by a native English speaker. The vocabulary needs a revision (see reviewer #2) to be consistent with the literature and through the text. Both reviewers point out the need to complete the review of literature, including the scientific papers published in the last years (from 2016 to date).

I agree with the concerns of the reviewer #2 on the statistical analysis. Please, pay attention to provide a full reply to the comments about this issue. Moreover, I did not understand your description of the results about figures 6 and 7: I think that these trends are not significant, because the CIs are very large and they include the variations described by the regression lines.’

**Response: Our literature review has been updated to include studies upto 2020. We have rewritten much of the manuscript and clarified and added more details to our methods section. The title has changed to reflect the focus on mammals in protected areas. All models are new and be have focussed our modelling to 5 questions focussing on 1) global mammal declines in protected areas, 2) mammal declines in Africa, 3) mammal declines in Africa excluding elephants, 4) mammal declines in Africa - elephants only and 5) mammal declines in Asia. There are two ‘elephant’ models included because much of Africa data consists of elephant poaching studies. All the results we have presented are new and more focussed to out aims which we have explicitly set out in the introduction.**

## Response to reviewer 1

Abstract

Line 34 - It should be 1980-2014?

**Response: This has now been corrected and our analysis now includes data upto 2020 (line 33)**

Introduction

Line 75 - change "hunting" to "hunting/poaching"

**Response: We now use the term ‘illegal hunting’ throughout the manuscript instead of hunting, poaching or illegal activities.**

Line 76 - Insert "threat" in the "each threat can reduce..."

**Response: We have now rewritten the introduction, this original line has been removed.**

Line 95 - conjugate verbs in the past tense. E.g. change "review" to "reviewed".

**Response: We have now used past tense throughout the manuscript**

Line 96 - 1980-2014?

**Response: This has now been corrected to include data up to 2020**

Line 96 - Why only until 2014? A literature review paper should be updated. It has been 6 years and certainly many studies have been published across the globe and should be included in the analysis before the MS is considered for publication

**Response: Published studies up to 2020 have now been included in the analysis**

Line 98 - change "evaluate" to "evaluated".

**Response: We have now rewritten the introduction, this original line has been removed. Past tense is now used where necessary**

Line 99 - We assessed

**Response: We have now rewritten the introduction, this original line has been removed. Past tense is now used where necessary**

Line 106 - "Material and methods"

**Response: This has now been corrected, changed to ‘Material and methods (line 119)**

Line 107 - Data collection?

**Response: This has now been corrected, changed to ‘data collection’ (line 120)**

Overall, I recommend that MS be reviewed by an English native prior to publication.

**Response: Thanks, the lead author is not a native English speaker, but the current MS has now been reviewed and commented on by native English speakers to correct tense, grammar and spelling.**

Line 109 - What is the reason for being from 1950? Authors must define why this temporal cut. Also, in the abstract and introduction is written from 1980-2014?

**Response: Search was intended to capture all studies since 1950 to date, however no relevant paper was retrieved until 1980. 1950 was chosen as an arbitrary year, but we have now updated the MS to state why this is a review from 1980-2020.**

Lines 112-113 - Again, a review study of the scientific literature must be current. Therefore, it is important that the authors include data by 2019 at least.

**Response: Published studies up to 2020 have now been included in the analysis**

Lines 147-150 - The MS title is said to be a study of mammalian populations. It may be better to modify for vertebrates since the authors have used other taxonomic groups.

**Response: The current MS currently focuses on mammals**

Lines 165-167 - Again, if other taxonomic groups were analyzed, it is important to modify the title of the study.

**Response: As above, we have clarified that the study now only focuses on mammals**

Line 173 - "referred"

**Response: We have now rewritten the introduction, this original line has been removed. Past tense is now used where necessary**

Lines 227-228 - Importantly, the period included in the search was 1950-2014 and not 1980-2014. The fact that no studies were found until 1979 is a result.

**Response: This has been corrected and search period is reported appropriately (Line 122)**

Lines 288-289 - ??? **Re-written and Corrected**

Line 293 - ??? **Re-written and Corrected**

Line 333 - ??? **Re-written and Corrected**

Line 339 - ??? **Re-written and Corrected**

Line 360- ??? **Re-written and Corrected**

Line 364 - ??? **Re-written and Corrected**

**Response:The results section has been fully rewritten as a result of the peer review. We have now focussed our analysis on mammals, and have fewer GLMM models focussing on 1) global data, 2) Africa and 3) Asia**

Line 430 - ???

**Response: (Re-written and Corrected)**

The MS presents important information to understand how impacts on protected areas are distributed across the globe. And no doubt it is of great relevance to conservation. However, it is necessary to update the data before publication! A literature review study should be as current as possible at the time of publication. That is, authors must at least include data from papers by 2019.

**Response: Thanks. We have rewritten much of the MS, updated our literature search and compiled new models to focus our analysis on mammals.**

## Response to reviewer 2

The researchers of this paper made a review of the illegal activities affecting populations of different species inside protected areas (PAs). This is a very relevant topic because it is increasingly shown that protected areas are not enough to stop the biodiversity crisis we observe worldwide. Especially, in the last years, it has been shown that overexploitation and land-use change are the main drivers of biodiversity loss. However, the paper does not include any reference to recent studies (the most recent reference is from 2016) and therefore I think they missed some relevant and new insights on the topic. I believe that the manuscript and their database has potential for publication, yet, the manuscript was confusing in many parts, difficult to follow, and not presented in an attractive way so, in my opinion, it requires a profound revision from the authors to clarify several aspects and improve the scientific basis of it.

In general, I think that more attention needs to be paid to the vocabulary and the overall aim of the paper. In the introduction (and title) they talk about illegal activities, however, in the methods they explain that they only included illegal extraction and no other illegal activity as such for the analysis (criteria ii). I would frame the paper differently because otherwise, the reader expects a different outcome from it. Moreover, the methods were really short and some decisions that they made were difficult to understand, e.g., why put all non-mammals together? The dataset although relatively large is too patchily distributed and it should be refined (filtered) before being analysed. I suggest to restrict the analysis to Africa as most of the data (~80%) is from there. In contrast to the methods, the results were quite extensive with some non-informative figures that could be merged or eliminated, tables that could be clearer, and paragraphs that also need clarification (see comments). Overall, the discussion was easier to follow than other parts of the paper, however, as I had difficulties following the general setup and I think that some of the results cannot be fully trusted due to low data points, most of my specific comments regard the methods.

**Response: Many thanks for the reference suggestions. We have incorporated the latest literature and rewritten and much of the manuscript to focus on mammal and PA effectiveness. We have clarified our aims and questions (lines 111-116) and ensured that the data analysis uses the most suitable database subsets; 1) a global, 2) three models focussing on Africa and 3) a model focussing on Asia. The Database used is supplied.**

Title

The main title is misleading as it talks about illegal activities, while the paper is about poaching only. Therefore, the short title is much better as it focusses on the poaching impact. Please, consider changing the title

**Response: This has now been changed to reflect the updated analysis and focus of the manuscript on mammals. It now reads “Global extent and drivers of mammal population declines in protected areas under illegal hunting pressure”**

Introduction

Lines 66-71: Please revise recent literature on large-scale patterns on the efficiency of PAs (e.g. Geldmann et al. 2015, 2019) and wilderness areas (Di Marco et al. 2019) to protect biodiversity. Also check the effect of hunting and land use on biodiversity worldwide (Brodie et al. 2015; Ripple et al. 2016; Benítez-López et al. 2017, 2019; Gallego‐Zamorano et al. 2020) because in your citations, you only focus on local assessments but there has been an effort on the last years to assess overall impacts of these pressures. This also applies to other parts of the text e.g. Lines 91-93 and discussion.

**Response: Many thanks for these suggestions. Our introduction (and discussion) have been fully rewritten to include up-to-date literature and more relevant citations. We believe our manuscript stands apart from recent similar analysis (e.g. Benítez-López et al. 2017, 2019) because our interest is in the population impact within PAs and therefore a broad scale assessment of PA effectiveness using specific PA population trend data.**

Line 96: Both here and in the abstract the years (I supposed) are wrong, please correct.

**Response: This has now been corrected and literature search updated to include all articles up to end of 2019 and early 2020 (line 33)**

Methods

Lines 115: Until now, you talked only about illegal activities in general and mentioned illegal extraction (poaching) as one of them. Here, you restricted your search to only studies that included illegal extraction (criteria i and ii), which is fine but mislead the overall aim of the paper.

**Response: The current manuscript focuses on illegal hunting. Our database is also restricted to mammals.**

Line 127-129: What if several studies satisfied all the criteria, how did you select the paper?

**Response: It was not possible for a paper to satisfy all criteria. For example a paper can be both a review and primary data paper. We have clarified the criteria used in the data selection (lines 127-133)**

Line 134: But logging was not included in the analysis right?

**Response: Correct, our updated analysis focuses on mammals for which the data are most numerous. Reference to ‘logging’ has been removed**

Line 139-140: I would like to know all the information that you put in the database and see the full database that you used for the analysis. Please, remove the “etc” and name all the variables, do not be short in words.

**Response: We have now clarified and specified the data collated in the database (lines 140).**

**A full dataset with all the variables included in this analysis is attached as supplementary material.**

Lines 140-143: Do you use meta-analytical tools for the analysis or not? If yes, which type of weighting method did you use for each study (sample size, variance)? How can I believe that your analyses are robust? Reference 18 is not suitable for this statement.

**Response: We assessed performance of all model analysis using Akaike Information Criterion (AIC) and selected the most parsimonious model after the stepwise removal of terms (Lines 189-191). Ref 18 replaced by a more appropriate citation (Line 144).**

Line 149: The fish data is missing in SM1, is all the body mass coming from primary sources?

**Response: All non-mammal taxa have now been excluded from the current analyses.**

Line 155-162: As you focus on population changes of species inside PAs, could you use spatial analysis to calculate for example the agricultural land-use change inside each of the PAs? I propose this because PAs, in general, are not big and species suffer more from local-scale changes (e.g. deforestation or hunting in the area) than from country-level changes. Some areas of a country can be heavily degraded while others are intact, therefore a more detailed and spatially-explicit analysis will benefit the paper in my opinion.

**Response: We think using a country-level agricultural land use change data was appropriate given that HDI was also a country-level index. Also evidence from literature suggests increased pressures associated with land use demand in the wider matrix often squeezes protected areas border and degrate inside PAs through agriculture (Lines 160-162).**

Lines 164-165: If the purpose of your study is to assess the effectiveness of PAs against illegal activities, specifically illegal extraction, please consider to re-do the levels of PAs and stick to PAs that are designated at conserving biodiversity. Categories I‐IV, are designated for that, with I-II being stricter. Categories V‐VI allow sustainable use (see Santini et al. 2016; Oldekop et al. 2016).

**Response: Our search criteria filtered PAs that allow legal hunting and in our initial analysis we conducted separate analyses involving both stricter and less strict PAs to decouple the potential confounding effect of potential legal hunting in less strict PAs. We agree that the analysis should be restricted to protected areas designated at biodiversity conservation. Therefore, our new analysis has removed all data points in IUCN categories V and VI. The spatial distribution (figure 1) and temporal trends (figure 2) do still include records from all data points.**

Lines 167-170: This can mislead the results because reptiles, birds, fishes and mollusc have very different ecological requirements and their extraction is very different. If the data was poor, then I would simply not use it. Same applies to the spatial data, I would not include the European study nor (maybe) the American ones.

**Response: We have now restricted the data analysis to mammals only and removed the European data. As this is a global analysis, we have still included the Central and South America data (many more data points from the updated literature search) in the global model, but we have also focussed on the area with most data in separate analysis, specifically Africa and Asia.**

Lines 171-209: In this paragraph, I would specify more explicitly that the response variable of your models is species decline (1) or not (0). This is mentioned in the previous section but as an example, so here I was a bit confused about which response variable you used.

**Response: We have now made it explicit in the text that the response variable in all models is species decline in a PA (1) or not (0). (line 178)**

Line 183: Please be consistent with the vocabulary. Here, you use poaching type, but in line 194 you use hunting level (same applies to the whole paper). Poaching refers to illegal hunting, but the level of hunting is something different, e.g., intense vs not intense hunting. I would not use poaching and hunting interchangeably as hunting can be legal.

**Response: We now use ’illegal hunting’ throughout the text**

Line 188: Which datasets? Do you have more than one?

**Response: This has been corrected. Our term ‘datasets’ represent different subsets of the illegal hunting database used to answer or specific questions. (line 195)**

Lines 195-197: How did you do this? Please, expand the explanation.

**Response: We have now re-written these lines to clarify that all models included these variables prior to model selection. (lines 178-9)**

Line 202-204: How about using cross-validation instead of a subset data specifically for elephants? What is sufficient data in this case?

**Response: This has been corrected. Cross validation of GLMMs would not be appropriate in this analysis. We therefore use separate GLMMs on subsets of data to answer our specific questions which now focus on 1) global data, 2) Africa (with and without elephant data) and 3) Asia. We have excluded analysis focussing in PA strictness as it’s not appropriate, but sufficient data would be models that include at least 5 blocks of random effects (in this case our random effects were study, country and protected) (lines 178-200)**

Line 204-206: So if I understood correctly you have three sets of data, in all sets, you started from a global model and did backwards removal to assess the generality of the effects, is this correct? Did you use any model selection criteria (AIC or BIC) to assess if the model with all the significant variables was the best or not? Moreover, as I said, I would stick only to the continents with enough data (Africa and Asia) the rest are quite scarce (maybe even only Africa as it accounts for 80% of your data). Figure 1 shows how this is a problem with very few points in Europe and America.

**Response:We have now clarified our model selection process, which did originally include AIC model selection criteria. As noted earlier, we have focussed our data analysis to areas where most of the data are 1) globally, 2) africa and 3) asia. FIgure 1 still includes all illegal mammal hunting records in the database, but some data (e.g. from Europe) were not used for analysis.**

Results

In my opinion, there are too many figures (8 in total) and some of them are not very informative, please, consider to remove some of them or combine them. For instance, Figure 2 could be replaced with text.

**Response: Agreed. We have limited our descriptive results to spatial and temporal trends. Our original questions have been refined so that we now use fewer models in the analysis.**

Lines 246-252: These two paragraphs could be merged in one, Figure 3 already shows the increase in studies so no need for Figure S1. Moreover, is there a reason for the different colors in Figure 3?

**Response: We have replaced all figures in the main text and supplementary data to highlight our more focussed analysis.**

Line 263-264: These numbers also show a strong biased towards mammals with very low numbers for the rest of the groups, therefore, I would suggest to completely remove them from the analysis and not include them as “non-mammals”.

**Response: Agreed, our new analysis now focuses on mammals throughout the manuscript**

Line 286-292: This paragraph and Table 1 are really confusing. You mix all different models and is really difficult to follow. Why Figure 6b is the model without African elephants? And did you only removed African elephants or all elephants as mentioned in line 200? Again, please be consistent with terminology. Moreover, I don’t understand Model 1, how is it possible that none of the poaching types (not levels) is significant when including the elephants? Aren’t elephants very influenced by poaching (line 202)?

**Response: Our analysis has now been refined to 5 GLMMs. We still include a model with and without savanna elephants (*Loxodonta africana*) but have clarified why these subsets of the database have been used, i.e., due to a high number of records due to to the research interest in elephants and ivory (line 178-200)**

Line 302: I thought Figure 6a is for Model 1 (line 288-289) but in the caption, you put that is for the model without elephants, can you clarify it?

Line 315: I think you confuse the terminology here is not best explained but significantly affected by agricultural change. Could you provide the explained variance by each of the models, i.e. marginal and condition R2?

**Response: It is not possible to calculate traditional *R^2^* values for GLMMs due to the influence of the random effects. We have however computed pseudo *R^2^* for each model (Table 1) based on the squared correlation between the response variable and the predicted values. These pseudo *R^2^* should be interpreted with caution as it only shows the variance explained by the fixed effects in the final model. We have stated this in the text also (lines 191-194)**

Line 356: Did you actually removed Europe? How about Central America? Why did you combine Latin America+Asia?

**Response: As noted above, we have now refined the data analysis to exclude Europe and focus on 1) global, 2) africa and 3) Asia.**

Line 360: So the stricter the more decline?

**Response: We have refined the analysis to focus on particular subsets of the database, but have still included a model on data in Asia. We have clarified this result, and yes, mammal populations in stricter PAs (IUCN categories I-II) had a greater probability of decline compared to less strict PAs (IUCN categories III-IV). (lines 252-254)**

Discussion

Please, do not talk about illegal activities in general through all the discussion as you only included illegal extraction (poaching) in the analysis, focus on that.

**Response: Yes, as with the rest of the improved MS, we have focussed the discussion on illegal hunting rather than refer to illegal activities.**

Lines 376-377: Could you maybe create different body size groups to test this?

**Response: Because we were not clear in the original MS about the analysis, we feel this is not necessary. Body mass was included in all updated models (except model 4, elephant only) as a continuous variable. Splitting the data into groups does produce the same general result i.e, population decline as a result of illegal hunting increase with body size.**

Line 378: You did not calculate habitat loss as such, even less at the PA scale. Is there any animal in your list that have affinity for agricultural areas? If yes, then the increase in agriculture for them is not habitat loss.

**Response: Agriculture activities around borders and inside PAs degrade habitats which then affect mammals. Yes, we agree that the effect of habitat degradation, thus loss, may be variable among species due to species-specific responses but still its impact is often considerable for most species. Thus we still believe that a country-level agricultural land use change will have an impact on the preserved areas which are potentially suitable for agriculture. We have provided text to clarify this (line 159-160)**

Suggested references

**Response: We have considered these literature in the re-written introduction of the MS.**

Benítez-López A, Alkemade R, Schipper AM, Ingram DJ, Verweij PA, Eikelboom JAJ, Huijbregts MAJ. 2017. The impact of hunting on tropical mammal and bird populations. Science 356:180–183. Available from: <http://science.sciencemag.org/content/sci/356/6334/180.full.pdf>.

Benítez-López A, Santini L, Schipper AM, Busana M, Huijbregts MAJ. 2019. Intact but empty forests? Patterns of hunting-induced mammal defaunation in the tropics. PLOS Biology 17:e3000247. Available from: <https://doi.org/10.1371/journal.pbio.3000247>.

Brodie JF, Giordano AJ, Zipkin EF, Bernard H, Mohd-Azlan J, Ambu L. 2015. Correlation and persistence of hunting and logging impacts on tropical rainforest mammals. Conservation Biology 29:110–121. John Wiley & Sons, Ltd. Available from: <https://doi.org/10.1111/cobi.12389>.

Di Marco M, Ferrier S, Harwood TD, Hoskins AJ, Watson JEM. 2019. Wilderness areas halve the extinction risk of terrestrial biodiversity. Nature 573:582–585. Available from: <https://doi.org/10.1038/s41586-019-1567-7>.

Gallego‐Zamorano J, Benítez‐López A, Santini L, Hilbers JP, Huijbregts MAJ, Schipper AM. 2020. Combined effects of land use and hunting on distributions of tropical mammals. Conservation Biology:cobi.13459. Available from: <https://onlinelibrary.wiley.com/doi/abs/10.1111/cobi.13459>.

Geldmann J et al. 2015. Changes in protected area management effectiveness over time: A global analysis. Biological Conservation 191:692–699. Available from: <http://www.sciencedirect.com/science/article/pii/S0006320715300793>.

Geldmann J, Manica A, Burgess ND, Coad L, Balmford A. 2019. A global-level assessment of the effectiveness of protected areas at resisting anthropogenic pressures. Proceedings of the National Academy of Sciences:201908221. Available from: <http://www.pnas.org/content/early/2019/10/22/1908221116.abstract>.

Oldekop JA, Holmes G, Harris WE, Evans KL. 2016. A global assessment of the social and conservation outcomes of protected areas. Conservation Biology 30:133–141. John Wiley & Sons, Ltd. Available from: <https://doi.org/10.1111/cobi.12568>.

Ripple WJ et al. 2016. Bushmeat hunting and extinction risk to the world’s mammals. Royal Society open science 3:160498. The Royal Society. Available from: <https://www.ncbi.nlm.nih.gov/pubmed/27853564>.

Santini L, Saura S, Rondinini C. 2016. Connectivity of the global network of protected areas. Diversity and Distributions 22:199–211. Available from: <http://doi.wiley.com/10.1111/ddi.12390>.
