# Supplementary material for: Global extent and drivers of mammal population declines in protected areas under illegal hunting pressure
Source: PLoS One. 2020 Aug 21;15(8):e0227163. doi: 10.1371/journal.pone.0227163 (PMC7446782; doi:10.1371/journal.pone.0227163)
Supplement: Attachment — Submitted filename: Review_PONE-D-19-34271_R1.docx [file pone.0227163.s005.docx]

**Review: PONE-D-19-34271**

The authors have done a thorough and impressive job of responding to my (and the other referee's) comments. The manuscript is now much more attractive, with beautiful and useful figures, and it is easy to read. Moreover, the manuscript's narrative is now much better aligned with the methods and results and, consequently, makes a compelling case (in my view at least). Congratulations on the big effort and nice result. However, I feel that some of the results need some more clarification and that the discussion needs to be expanded in some parts to give more insights on some of their nice results. Therefore, I have a few minor comments to offer at this point that I think need to be addressed before acceptance.

**Minor comments**

**Title**

The title is now more suitable for the research done.

**Introduction**

Line 64: Do you mean: “Despite the challenges of protecting area…”? Can you clarify the sentence?

Line 69-72: The references here need to be corrected. Benitez-Lopez et al. 2017 and 2019 focused on hunting only not on logging, Allan et al 2019 is a general view with many impacts not only hunting. Gallego-Zamorano et al 2020 combined both land-use change (e.g. cropland and pasture expansion) and hunting impacts on the distribution of species without using abundances. Please check that the citations are correct within the context of the text.

Line 85: change “a” for “the” and the comma for “and”: At the local and individual protected area level. Or simply remove “At a local”.

Line 85-90: This paragraph is a bit short and does not add much to the rest of the introduction. Please consider to mix it with the previous one.

**Methods**

The methods are much clearer now and robust.

Lines 134-137: You are missing some full stops; I guess due to the track changes tool. “data collection method. We recorded”; “hunting (e.g. timber extraction). Each species”

Line 157: human development “index” (HDI)

Line 183-184: I am curious to know if there were different studies for the same species in the same PA. Did you check if the inclusion of species as random effect improved the models? How did you assign the random effects structure (e.g. model selection for the random effects)?

Lines 191-194: So this is the “marginal R^2^”, could you provide also the “conditional R^2^” which represents the variance explained by both the fixed and random factors.

Lines 195-200: This is clear now, and makes sense.

**Results**

Congratulations on the improvement of the figures, they are much more attractive now and clear.

Fig.1: As an idea you could also include the shape of the protected areas so you do not only show the centroid but the full shape of the area.

Fig.2: Interesting that there are only market surveys in the 2000s period. Do you have an explanation for that? At this point, the reader gets to this figure and wonders which are the differences for the different categories of methods, e.g. interviews vs interviews/count? I suggest to include a small definition of each category in the methods (maybe as a table) so it is clearer for the reader.

Line 226: How many observations (species/site combinations), species, PAs, were included in the final model at the end? I also wonder which was the final structure of each model after the model selection? What happened with poaching type, ALC, and continent for example? I think this part needs a bit more explanation to understand properly all the models.

Table 1. I realized that you put the observations here but I would add some more numbers in the paragraph. Also in the table please add all the estimates even if they were not significant, don’t hide results.

Fig.4: I am curious about the trend if you combine both graphs, i.e. in the X with body mass and colored by HDI. Did you try in the models any interaction like this one?

Fig.5: This is one of the most interesting results, see my comments in the discussion.

**Discussion**

Line 290: remove the full stop in the middle of the sentence.

Lines 300-302: Why habitat loss? ALC was not in the best model right?

Lines 303-305: Is it low HDI related to ivory trade? Can you expand on this?

Line 309: You can add the citations of Benitez-Lopez et al 2019 and Gallego-Zamorano et al 2020.

Lines 309-318: I think diseases or protein content are not the main cause of the reduction of large mammals, poaching and wildlife trade are the ones causing their decline. They are hunted not only for meat consumption but for their body parts, that together with the reduction of habitat is creating their decline. Diseases and protein content are simply additions to it. See for example Ripple at al 2019.

Lines 325-326: Missing a full stop. Moreover, I am not convinced by the reason here. You could test what is the effect of poaching type in Asia to see why in stricter PAs there is more decline. This is one of the most interesting results and I miss some more insights on it. If they are stricter there should be also less declines and less habitat loss and poaching but you are showing the opposite, why? Could it be because more precious animals are in stricter PAs and the wildlife trade market in Asia is bigger than in other areas? I am thinking about pangolins, sun bears, orangutans…etc.

**Supplementary material**

Meta-data: Could you expand the explanation of the meta-data, including all the levels of the different categories in different columns?

FigS1: Is not necessary anymore I think

Table S2: Needs to be updated to remove other taxons than mammals

SM1: Needs to be updated to remove other taxons than mammals
