# Supplementary material for: Global extent and drivers of mammal population declines in protected areas under illegal hunting pressure
Source: PLoS One. 2020 Aug 21;15(8):e0227163. doi: 10.1371/journal.pone.0227163 (PMC7446782; doi:10.1371/journal.pone.0227163)
Supplement: Attachment — Submitted filename: Response to reviewers.docx [file pone.0227163.s006.docx]

**Response to reviewers’ comments - revision 2 - PONE-D-19-34271**

**Spatial distribution and research trend of illegal activities and the factors associated with wild mammal population declines in protected areas**

**Response to Editor**

I appreciated the impressive job of responding to comments and increasing the quality of the manuscript. Both reviewers agree to point out this result, even if the reviewer #1 suggests to add some articles in your review.

Although I noted the big efforts to improve the writing of the manuscript, I agree with reviewer #1 to request you a further revision in the use of specific terms and acronyms.

I feel that the method section needs a more detailed description of the statistical analysis, particularly of the methods used to estimate the explanatory variables. Finally, I would like to see a broader description of the results in order to better highlight the findings of the manuscript and to make them easier to understand for readers.

**Response: We have provided details why each of the articles suggested by reviewer #1 should not be included in this review in our response to Reviewer 1’s comments - none of these articles meet the selection criteria during the literature review process.**

**The methods section has been expanded to provide more details on the analysis, particularly why certain explanatory variables were used (e.g. Human development index and Corruption index were highly correlated, but HDI data were available across the whole study period). WE have also expanded on the details of each model structure.**

**We have expanded the descriptive results section to give a better overview of the scale of studies, types of conservation areas and purpose of the research**

**Acronyms and terms have also been checked throughout and are now used appropriately.**

**NB: [The map used in Figure 1 was sourced from Natural Earth, which is an open access map source].**

**Response to reviewer 1**

The second version of the MS submitted by the authors presented a text that was difficult to understand, which complicated decision making. The study is important, however, it needs a careful editing of the text to improve reading. There is a lack of standardization in writing and abbreviations, terms, etc. Furthermore, I believe that the review conducted by the authors is incomplete and biased, because several studies conducted in Brazil that showed the impact of illegal hunting on mammals have not been mentioned.

My biggest concern is the writing of the MS and the absence of some important references.

**Response: We have now checked through the abbreviations and acronyms throughout the manuscript and have changed them appropriately, including: PA, ALC and HDI.**

See my comments in the PDF file.

Abstract

**Lines 37-40 edited to improve readability**

**Lines 42-45 edited to improve readability**

**Line 45 - “mounting” changed to “growing**

**Keywords have now been edited (Line 49-50)**

Introduction

Line 55: Authors should abbreviate to PAs and use in the next sentences.

**Response: ‘PAs’ is now used to abbreviate protected areas throughout the text (Line 54 onward)**

Lines 72-75

**Response: Example has been added to improve the sentence and writing**

Also, in the tropical forest, declines by 80% and 30% have been reported for mammalian populations caused by hunting and land use change [9, 11], consequently leading to a potential loss of species interaction networks [18, 19].

**Response: Thanks for the suggestion, this has now been changed (Lines 72-74)**

L75: The authors should define the terms related to the types of hunting, or cite a study that defines the different types of hunting.

**Response: A definition of bushmeat hunting is now provided with an appropriate citation (Line 74)**

L77: Same problem previously mentioned about the sentence writing.

**Response: This sentence has now been changed to improve clarity (Line 76-7)**

L78-79: How so? How can illegal and legal hunting occur simultaneously?

**Response: In PA categories that allow controlled legal hunting such as some IUCN Category IV (i.e. Game Reserve, e.g. Selous Game Reserve in East Africa) are often faced with illegal hunting due to limited or weak law enforcement patrols. (Line 77-78)**

L83: Same writing problem. There is no proper use of commas in the sentence. This is important in writing so that reading is not accelerated.

**Response: Commas have now been inserted as appropriate (Line 86)**

L85: That sentence didn't make sense to me. Please rephrase!

**Response: This sentence has been rephrased to improve clarity (Line 79)**

Line 87: Taste = meat flavor

**Response: ‘Taste’ has been maintained in the text because it is a term commonly used in illegal bushmeat consumption literature (e.g. Ripple et al 2016; Ndibalema and Songorwa 2008) (Line 81-82)**

L104: Suddenly an abbreviation appears that was not mentioned in any previous moment.

**Response: PAs abbreviation has now been used throughout the text (Line 54 onwards)**

L109-110: we aimed to:

**Response: This phrase has been adopted (Line 106)**

Materials and Methods

L121: References that authors should cite to add in the global review:

**Response: All suggested papers do not meet criteria for inclusion in the review as detailed under each suggested paper below**

- Ferreguetti, Á. C., Pereira-Ribeiro, J., Prevedello, J. A., Tomás, W. M., Rocha, C. F. D., & Bergallo, H. G. (2018). One step ahead to predict potential poaching hotspots: Modeling occupancy and detectability of poachers in a neotropical rainforest. Biological conservation, 227, 133-140.

**Response: This paper studies spatial distribution of poaching and the habitat/environmental covariates associated with it. It does not quantify poaching impact on the target wildlife population, thus, it does not meet criterion (II) for inclusion in the review**

- Ferreguetti, Á. C., Tomás, W. M., & Bergallo, H. G. (2015). Density, occupancy, and activity pattern of two sympatric deer (Mazama) in the Atlantic Forest, Brazil. Journal of Mammalogy, 96(6), 1245-1254.

**Response: This study compared abundance and produced evidence for niche partitioning between two sympatric deer. It does not investigate or quantify poaching or its impact on these species populations to meet criteria (I & II) for inclusion in the review**

- Ferreguetti, A. C., Tomas, W. M., & Bergallo, H. G. (2016). Density and niche segregation of two armadillo species (Xenarthra: Dasypodidae) in the Vale Natural Reserve, Brazil. Mammalian Biology, 81(2), 138-145.

**Response: This paper investigates density and niche separation of two species with poaching as covariate that influence detectability but not impact on population size of armadillos. This does not meet criterion (II) for inclusion in the review.**

- Ferreguetti, Á. C., Tomás, W. M., & Bergallo, H. G. (2017). Density, occupancy, and detectability of lowland tapirs, Tapirus terrestris, in Vale Natural Reserve, southeastern Brazil. Journal of Mammalogy, 98(1), 114-123.

**Response: This study compared abundance and produced evidence for niche partitioning between two sympatric deer. It does not investigate or quantify poaching or its impact on these species populations to meet criteria (I & II) for inclusion in the review**

- Ferreguetti, A. C., Tomas, W. M., & Bergallo, H. G. (2019). Patch occupancy and activity pattern of the spotted paca (Cuniculus paca Linnaeus, 1766) in a protected area of the Atlantic Forest, Brazil. Mammalia, 83(4), 363-371.

**Response: This paper talks about habitat use and resource partitioning in the spotted paca. It is not on poaching or on protected areas. Thus does not meet criteria I & II set for inclusion in the review**

- Ferreguetti, A. C., Davis, C. L., Tomas, W. M., & Bergallo, H. G. (2018). Using activity and occupancy to evaluate niche partitioning: the case of two peccary species in the Atlantic Rainforest, Brazil. Embrapa Pantanal-Artigo em periódico indexado (ALICE).

**Response: This paper talks about habitat use and resource partitioning in peccary species. It is not on poaching or on protected areas. Thus does not meet criteria I & II for inclusion in the review**

- Ferreguetti, A. C., Rocha, C. D., & Bergallo, H. G. (2019). Poaching in non–volant mammals in the Neotropical region: the importance of a metric to assess its impacts. Animal Biodiversity and Conservation, 42(2), 203-211.

**Response: This paper is a review work that analyses literature on poaching published for the Neotropical region. It does not meet criterion (III) for inclusion in the global review**

**Just if case it might be relevant here, we would like to note that COPE guidelines on the ethics of publication state that reviewers should “refrain from suggesting that authors include citations to your (or an associate’s) work merely to increase citation counts or to enhance the visibility of your or your associate’s work; suggestions must be based on valid academic or technological reasons.” It seems these reasons are lacking for the suggested papers.**

**L134:** insert comma

**Response: Comma inserted as suggested (Line 129)**

**L137:** insert period

**Response: Full stop inserted as suggested (Line 133)**

**L147-8:** Which ones?

**Response: Citations inserted showing sources of the BM for the two species (Line 148)**

**L156-**8: Rephrase this sentence.

**Response: Sentence rephrased to improve clarity (Line 156-7)**

**L158:** We should not start sentences with abbreviations!

**Response: ALC is written in full at start of sentence (Line 160)**

Data Analysis

**L174-6**: Any specific package?

**Response: This is now stated - we used the package ‘lme4’. (Line 179)**

**L180:** Suddenly, the authors start using the term poaching. Please adopt a standard in the text.

**Response: This has now been edited -’illegal hunting’ has been adopted throughout the manuscript**

**L185-6**: This should be informed above!

**Response: details of random effects selection are now provided in the previous sections of the methods text (Line 185-7)**

Results

**L205**: replace satisfied with qualified or met

**Response: met is adopted (Line 209)**

**L232**: Greater?

**Response: ‘Greater’ is maintained as higher or low is not appropriate when referring to body mass (Line 248)**

Discussion

**L274:** Delete this sentence

**Response: This sentence has been deleted. Thus, the paragraph starts with the proceeding sentence (Line 283)**

**L286**: change Greater to higher

**Response: Suggestion adopted (Line 294)**

**L297**: provide(d)?

**Response: Past tense adopted (Line 342)**

Conclusion

**L347**: suggest(ed)?

**Response: Past tense adopted (Line 366)**

**Response to reviewer 2**

The authors have done a thorough and impressive job of responding to my (and the other referee's) comments. The manuscript is now much more attractive, with beautiful and useful figures, and it is easy to read. Moreover, the manuscript's narrative is now much better aligned with the methods and results and, consequently, makes a compelling case (in my view at least). Congratulations on the big effort and nice result. However, I feel that some of the results need some more clarification and that the discussion needs to be expanded in some parts to give more insights on some of their nice results. Therefore, I have a few minor comments to offer at this point that I think need to be addressed before acceptance.

**Response: Thank you. We’ve expanded on and clarified the results section as suggested (e.g. the results table). We have also clarified parts of the discussion, particularly why understanding poverty is important because of the relationship with illegal activities and the ivory trade. We have also re-written our discussion points on 1) why larger bodies species might be at greater threat of population declines from illegal harvesting with examples and 2) why the most strictly protected areas in Asia show larger mammal population declines compared to other protected areas.**

Title

The title is now more suitable for the research done.

Introduction

Line 64: Do you mean: “Despite the challenges of protecting area…”? Can you clarify the sentence?

**Response: This has now been clarified to “challenges to protecting biodiversity” (line 62-3)**

Line 69-72: The references here need to be corrected. Benitez-Lopez et al. 2017 and 2019 focused on hunting only not on logging, Allan et al 2019 is a general view with many impacts not only hunting. Gallego-Zamorano et al 2020 combined both land-use change (e.g. cropland and pasture expansion) and hunting impacts on the distribution of species without using abundances. Please check that the citations are correct within the context of the text.

**Response: Theses citations have now been corrected**

Line 85: change “a” for “the” and the comma for “and”: At the local and individual protected area level. Or simply remove “At a local”.

**Response: “At a local” has now been removed from this sentence**

Line 85-90: This paragraph is a bit short and does not add much to the rest of the introduction. Please consider to mix it with the previous one.

**Response: Agreed! This has now been incorporated with the previous paragraph (lines 79)**

Methods

The methods are much clearer now and robust.

Lines 134-137: You are missing some full stops; I guess due to the track changes tool. “data collection method. We recorded”; “hunting (e.g. timber extraction). Each species”

**Response: This has now been corrected (lines 132)**

Line 157: human development “index” (HDI)

**Response: “index has now been added” (lines 157)**

Line 183-184: I am curious to know if there were different studies for the same species in the same PA. Did you check if the inclusion of species as random effect improved the models? How did you assign the random effects structure (e.g. model selection for the random effects)?

**Response:**  **Although there is some duplication of species, most species are represented by a single study, and almost all appear only once in a given PA. So, because of this, we did not include species and site PA as random effects as including them would very likely result in model overfitting.**

Lines 191-194: So this is the “marginal R2”, could you provide also the “conditional R2” which represents the variance explained by both the fixed and random factors.

**Response: Conditional R2 value have now been added to Table 2, and a sentence in the methods highlighting the R package used to calculate these values (MuMIn) (Lines 194-6, 278)**

Lines 195-200: This is clear now, and makes sense.

Results

Congratulations on the improvement of the figures, they are much more attractive now and clear.

Fig.1: As an idea you could also include the shape of the protected areas so you do not only show the centroid but the full shape of the area.

**Response: We created a global map with the shape of PA as suggested, but have kept the centroid map as many PAs are relatively small and the ‘shape’ map does not add a great amount of detail to this global figure.**

Fig.2: Interesting that there are only market surveys in the 2000s period. Do you have an explanation for that? At this point, the reader gets to this figure and wonders which are the differences for the different categories of methods, e.g. interviews vs interviews/count? I suggest to include a small definition of each category in the methods (maybe as a table) so it is clearer for the reader.

**Response: A table (Table 1) with description of the method categories used in the manuscript and as reported in Fig 2 is provided.**

Line 226: How many observations (species/site combinations), species, PAs, were included in the final model at the end? I also wonder which was the final structure of each model after the model selection? What happened with poaching type, ALC, and continent for example? I think this part needs a bit more explanation to understand properly all the models.

**Response: Number of observations have now been added to the text in the results section ‘Socio-economic and species traits influencing population decline in PAs’ (Lines 241-269)**

**The final model structure is as Table 2 - i.e. only the significant fixed effects left in the model. Poaching type, ALC and continent were never significant variables or were in the best fitting models. We have included additional text to clarify structure of the final models - lines 278**

Table 1. I realized that you put the observations here but I would add some more numbers in the paragraph. Also in the table please add all the estimates even if they were not significant, don’t hide results.

**Response: The final models only included the significant fixed effects. This is now clarified on lines 278**

Fig.4: I am curious about the trend if you combine both graphs, i.e. in the X with body mass and colored by HDI. Did you try in the models any interaction like this one?

**Response: We have not attempted this interaction because we do not think there is an ecological reason for a relationship between mammal body mass and human development index.**

Fig.5: This is one of the most interesting results, see my comments in the discussion.

**Response: This result surprised us too, and we hope the discussion has been improved from the additional comments.**

Discussion

Line 290: remove the full stop in the middle of the sentence.

**Response: This sentence has now been edited (lines 298)**

Lines 300-302: Why habitat loss? ALC was not in the best model right?

**Response: Yes - this part of the sentence was unclear, but was referring to previous studies highlighting the importance of habitat change. We have removed ‘habitat loss’ to be clearer (lines 305-7).**

Lines 303-305: Is it low HDI related to ivory trade? Can you expand on this?

**Response: We have now included details on the link between poverty and the ivory trade and elephant population declines (lines 309-16). As a broad measure of economic deprivation and poverty we used HDI as this index is available globally. Poverty has been associated with elephant population declines and the ivory trade, but has used different measures (poverty density and infant mortality rate e.g. Hauenstein et al. 2019)**

Line 309: You can add the citations of Benitez-Lopez et al 2019 and Gallego-Zamorano et al 2020.

**Response: Thank you - these references have now been included in this sentence (Line 322)**

Lines 309-318: I think diseases or protein content are not the main cause of the reduction of large mammals, poaching and wildlife trade are the ones causing their decline. They are hunted not only for meat consumption but for their body parts, that together with the reduction of habitat is creating their decline. Diseases and protein content are simply additions to it. See for example Ripple at al 2019.

**Response: We have provided statements to explain body parts for traditional medicine and the links between HDI and human activities that lead to destruction of the wildlife habitats (lines 338-347)**

Lines 325-326: Missing a full stop. Moreover, I am not convinced by the reason here. You could test what is the effect of poaching type in Asia to see why in stricter PAs there is more decline. This is one of the most interesting results and I miss some more insights on it. If they are stricter there should be also less declines and less habitat loss and poaching but you are showing the opposite, why? Could it be because more precious animals are in stricter PAs and the wildlife trade market in Asia is bigger than in other areas? I am thinking about pangolins, sun bears, orangutans…etc.

**Response: Poaching level (i.e. ‘type’) was included in the full model, but it was not a significant variable during our model selection process. We have discussed this result more fully and provided three reasons to support our results on the wildlife declines in stricter PAs (lines340-7).**

Supplementary material

Meta-data: Could you expand the explanation of the meta-data, including all the levels of the different categories in different columns?

**Response: Yes, more details about all the levels of the different categories are now provided**

FigS1: Is not necessary anymore I think

**Response: Agreed - this has now been removed**

Table S2: Needs to be updated to remove other taxons than mammals

**Response: Table S2 is now renamed as Supplementary Material_ PART IV, included data for mammals**

SM1: Needs to be updated to remove other taxons than mammals

**Response: SM1 no longer needed- now deleted**
